# Janus-like Metallic Nanocrystals with Tunable Composition and Morphology Developed to Optimize their Catalytic and Photocatalytic Performance in the Reduction of 4‑NP and Organic Dyes

**DOI:** 10.1021/acs.langmuir.6c03367

**Published:** 2026-08-12

**Authors:** Chin-Chieh Yeh, Yu-Hsiang Chang, Su-Wen Hsu

**Affiliations:** Department of Chemical Engineering, 34912 National Cheng Kung University, Taiwan No. 1 University Road, East Dist, Tainan City 70101, Taiwan (R.O.C)

## Abstract

Highly heterogeneous Janus-type nanocrystals with tunable morphology and composition can be obtained through a “two-step continuous seed-mediated” method. These Janus-like nanocrystals, composed of active materials (Au and Pt) and plasmonic materials (Ag and Au), exhibit significantly higher catalytic performance in 4-NP and organic dye reduction reactions, approximately 1.5 to 5 times higher, compared to bimetallic nanocrystals with one-half of the Janus-like nanocrystal structure (prepared using conventional seed-mediated methods). This can be attributed to the greater number of active sites (surface area/volume ratio) and higher activity sites (synergistic effect) of the Janus-like nanocrystals. Furthermore, Janus-like nanocrystals, composed of superior plasmonic materials, can further enhance their catalytic performance by converting photon energy into chemical energy under irradiation with light of a specific wavelength (generating plasmon-induced hot electrons around the nanocrystals). Compared with the catalytic and photocatalytic performance of bimetallic nanocrystals Au–Ag and Pt–Ag reported in the literature, these Janus-like nanocrystals, with their highly heterogeneous morphology and composition, can degrade 4 to 10^2^ times more 4-NP molecules per unit weight of nanocrystals as catalysts or photocatalysts. These results demonstrate that by adjusting the morphology and composition of nanocrystals, specific chemical reactions can be accelerated, making the design of multifunctional heterogeneous nanocrystals through a “multistep continuous seed-mediated” method valuable.

## Graphical abstract

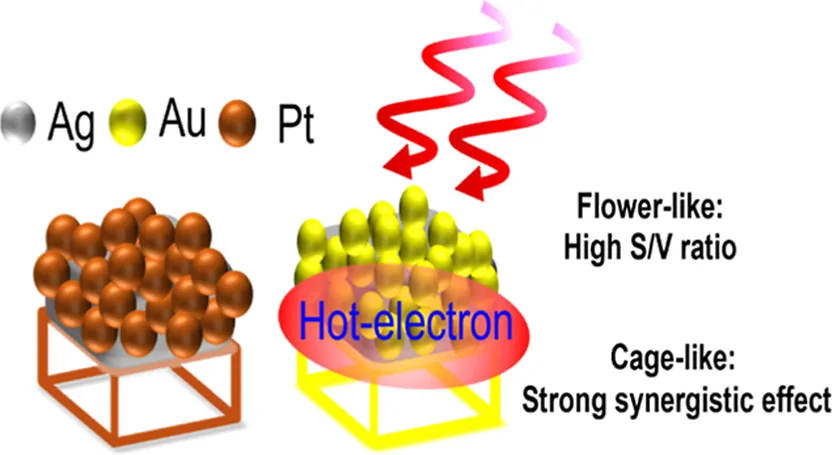

## 1 Introduction

Due to human activities, synthetic organic dyes are widely used in industrial and medical fields, such as textiles, paints, cosmetics, plastics, food colorings, and disinfectants, causing increasingly serious environmental water pollution. Converting these toxic substances in water into low-toxicity, recyclable products has become a research hotspot.^1–4^ Inorganic catalysts are widely used to treat organic dyes in aqueous solutions. Compared to single-component bulk materials, heterogeneous inorganic nanocrystals exhibit superior catalytic performance in various chemical reactions. This superior catalytic performance stems from the large number of active sites (a huge surface area/volume ratio) and the unique chemical reactions (synergistic effects) between different components on the nanocrystal surface.^5–11^

To synthesize heterogeneous nanocrystals with desired composition and morphology for use as catalysts in specific chemical reactions, several synthetic methods have been reported, such as photolithography or etching (top-down techniques) and colloidal and sol–gel methods (bottom-up techniques).^12–16^ The advantage of colloidal synthesis for preparing heterogeneous nanocrystals lies in the ability to control their composition and morphology by altering the precursors and chemical environment. However, colloidal synthesis still faces challenges in preparing multicomponent nanocrystals, including uneven component distribution and nanocrystal aggregation. To address the issue of uneven component distribution in nanocrystals, some studies have introduced seed-mediated methods in colloidal solutions, but nanocrystal aggregation still occurs.^17,18^ To address the aggregation problem of nanocrystals, our research group recently improved the seed-mediated synthesis method, immobilizing seeds on a polymer matrix to overcome the attractive forces between nanocrystals during synthesis. By controlling the chemical reaction mechanism, this synthesis method can be used to prepare bimetallic or metal–semiconductor nanocrystals with tunable composition and morphology, such as Au–Ag, Pt–Ag, and AgS–Ag nanocrystals, and uniformly disperse them on a polymer matrix.^9,10,19^ This seed-mediated method on polymer matrix surfaces can be further improved to “two-step continuous seed-mediated” synthesis of highly heterogeneous nanocrystals, namely Janus-like nanocrystals, which have two different morphologies and/or two different compositions on a single nanocrystal.^11^

Here, a two-step continuous seed-mediated synthesis method was used to synthesize six Janus-like nanocrystals composed of noble metals (Ag and Au) with excellent plasmonic properties or highly reactive metals (Pt and Au). The morphology of these nanocrystals was controlled by precisely modulating the chemical reaction mechanism, as shown in Figure 1A. The different morphologies of these Janus-like nanocrystals should endow them with different catalytic properties. Cage-like nanocrystals have a synergistic effect, while flower-like nanocrystals have a larger number of active sites (higher specific surface area).^10^ In addition, the different compositions of Janus-like nanocrystals should also endow them with different photocatalytic properties. Au–Ag nanocrystals have better plasmon resonance characteristics than Pt–Ag nanocrystals, as shown in Figure 1B. These Janus-like nanocrystals were used as catalysts and photocatalysts for the reduction of 4-nitrophenol (4-NP, organic dye precursor) and organic dyes in aqueous solutions, such as 4-nitrophenol (4-NP), methyl orange (MO), Congo red (CR), and rhodamine 6G (R6G) (common industrial molecular contaminants in water). Their catalytic performance was compared with that of bimetallic nanocrystals prepared via a “one-step seed-mediated” synthesis as catalysts and photocatalysts. Furthermore, the effects of the morphology and composition of the Janus-like nanocrystals on their catalytic and photocatalytic performance in the reduction of 4-NP and organic dyes were investigated.

**Figure 1:**
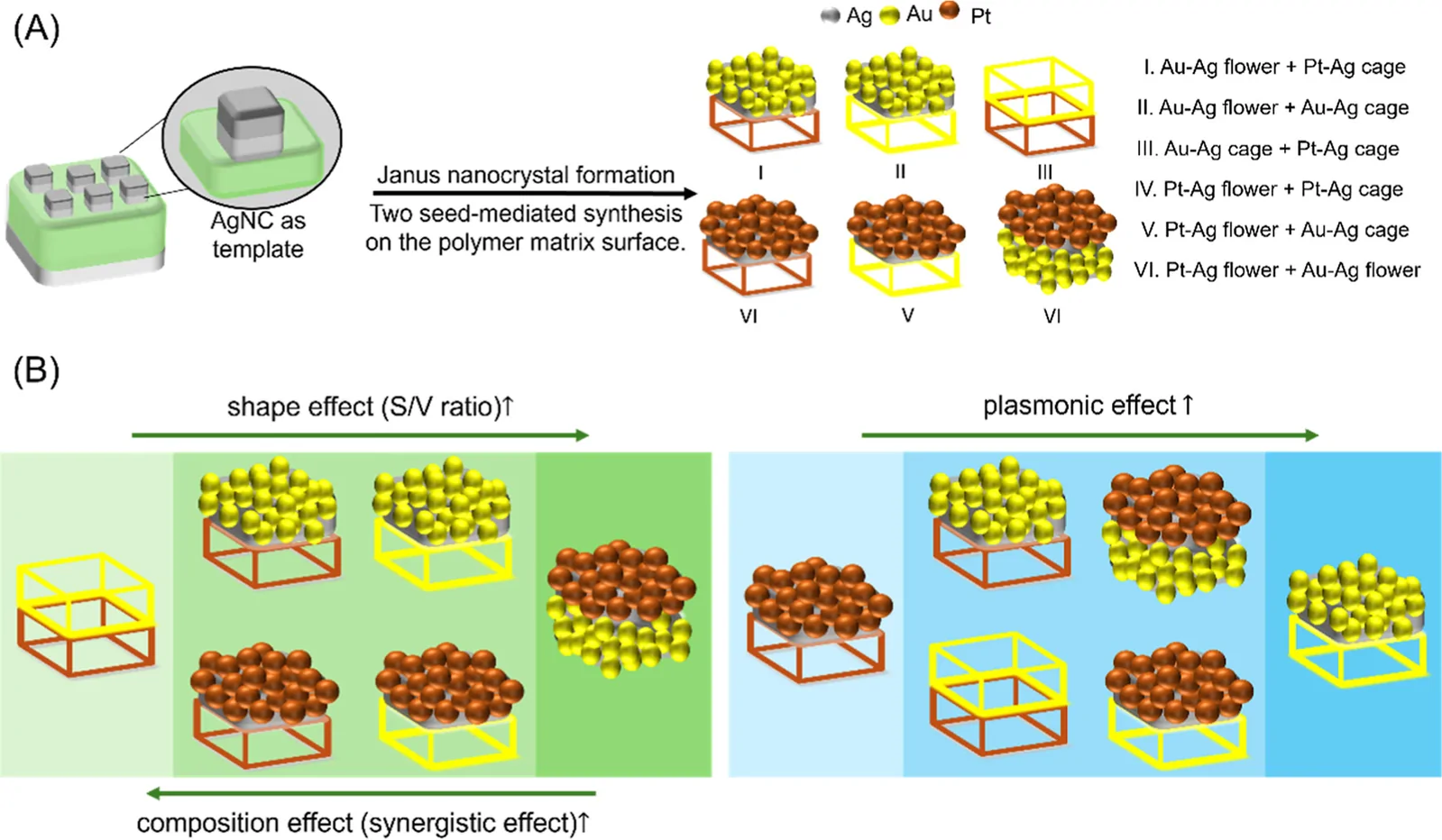
(A) Schematic illustrates the synthesis of Janus-like metallic nanocrystals with tunable composition and morphology via a “two-step continuous seed-mediated” synthesis method on a polymer matrix surface.^11^ Six different Janus-like metallic nanocrystals were prepared for catalytic and photocatalytic applications in the reduction of 4-NP and organic dyes: I: Au–Ag flower + Pt–Ag cage; II: Au–Ag flower + Au–Ag cage; III. Au–Ag cage + Pt–Ag cage; IV: Pt–Ag flower + Pt–Ag cage; V: Pt–Ag flower + Au–Ag cage; and VI: Au–Ag flower + Pt–Ag flower. (B) Based on the material properties of Janus-like metallic nanocrystals, three different parameters can be used to study their catalytic and photocatalytic performance in the reduction of 4-NP and organic dyes: shape effect (surface area/volume (S/V) ratio), composition effect (synergistic effect), and plasmonic effect.

## 2 Results and Discussion

Three metallic materials (silver, gold, and platinum) and two different morphologies (cage-like and flower-like) of nanocrystals were selected for constructing Janus-like nanocrystals. Ag and Au nanocrystals exhibit excellent plasmonic properties, making them suitable for photocatalytic applications, while Pt and Au nanocrystals demonstrate excellent chemical reactivity, making them suitable for catalytic applications. Since the cage-like structure is generated through galvanic replacement, it consists of an alloy of two metals; while the flower-like structure is generated by forming nanocrystals in an aqueous solution and then depositing them on seed crystals, it consists of two different metal nanocrystals.^10^ These two different morphologies of nanocrystals can produce two different effects to enhance catalytic performance: cage-like structures have a higher synergistic effect, while flower-like structures have a higher surface area-to-volume ratio.

Compared to spherical counterparts, Ag nanocubes (AgNCs) exhibit superior plasmonic properties due to their anisotropic shape and are used as seeds for the formation of Janus-like nanocrystals in “two-step continuous seed-mediated” synthetic methods. This method for synthesizing Janus-like nanocrystals includes the following steps: (1) the first seed-mediated reaction: an AgNC array was dip-coated onto a polystyrene (PS) matrix, and then the AgNCs were partially embedded into the PS matrix under heat treatment. By controlling the chemical reaction mechanism through a seed-mediated reaction, bimetallic nanocrystals with controllable composition and morphology were obtained. The four different bimetallic nanocrystals formed in this step (Au–Ag flower-like, Au–Ag cage-like, Pt–Ag flower-like, and Pt–Ag cage-like) will be used as catalysts for the reduction of 4-NP and organic dyes, and their performance will be compared with that of Janus-like nanocrystals. Four bimetallic nanocrystals (Au–Ag cage-like, Au–Ag flower-like, Pt–Ag cage-like, and Pt–Ag flower-like) were characterized using extinction spectroscopy, scanning electron microscope (SEM), transmission electron microscope (TEM), and energy-dispersive X-ray spectroscopy (EDX) mapping to analyze their optical properties, morphology, and composition, as shown in Supporting Information Figures S1–S4, respectively. (2) The bimetallic nanocrystals were reversely transferred onto a second polymer matrix (polydimethylsiloxane (PDMS)): A PDMS matrix was deposited on a bimetallic nanocrystal-PS substrate, and then a PDMS-bimetallic nanocrystal-PS sandwich structure was formed by heat treatment. Subsequently, the PS layer was selectively removed from the sandwich structure by immersion in acetone (acetone is a good solvent for PS but a poor solvent for PDMS). The unreacted part of the “seed” was exposed, and the bimetallic nanocrystal part was embedded in PDMS. (3) The second seed-mediated reaction: the unreacted part of the “seed” on the PDMS surface can form a third component on nanocrystals or different shapes on them to form Janus-like nanocrystals.^11^ Six different Janus-like nanocrystals were prepared using this method: I: Au–Ag flower-like structure + Pt–Ag cage-like structure; II: Au–Ag flower-like structure + Au–Ag cage-like structure; III: Au–Ag cage-like structure + Pt–Ag cage-like structure; IV: Pt–Ag flower-like structure + Pt–Ag cage-like structure; V: Pt–Ag flower-like structure + Au–Ag cage-like structure; and VI: Au–Ag flower-like structure + Pt–Ag flower-like structure. These Janus-like nanocrystals were characterized using extinction spectroscopy, TEM, and EDX mapping to analyze their optical properties, morphology, and composition, as shown in Supporting Information Figures S5–S10, respectively.

To maintain the same morphology for Au–Ag cage-like nanocrystals and Pt–Ag cage-like nanocrystals (approximately 10 nm within the cage-like framework), Au–Ag cage-like nanocrystals with an atomic ratio of Au/Ag = 14:86 and Pt–Ag cage-like nanocrystals with an atomic ratio of Pt/Ag = 20:80 were observed, as shown in Supporting Information Figures S1 and S3. This is due to the different galvanic replacement reactions between Pt^2+^ ions and Ag atoms and between Au^3+^ ions and Ag atoms: one Pt^2+^ ion can replace two Ag atoms on the seed surface, while one Au^3+^ ion can replace three Ag atoms on the seed surface. When the Au content in the Au–Ag cage increases, the rate of decrease in its framework size is faster than the rate of increase in the Pt content in the Pt–Ag cage. To compare the effect of pure morphology on the catalytic performance of 4-NP and organic dye reduction, it is necessary to obtain flower-like nanocrystals with a composition similar to that of cage-like nanocrystals, as shown in Supporting Information Table S1. Using these four bimetallic nanocrystals, six Janus-like nanocrystals with different compositions and morphologies were synthesized via a second seed-mediated synthesis method. The concentration ratio of Au/Ag and Pt/Ag in the nanocrystals generated in the second seed-mediated synthesis was controlled within a similar range to that of the products generated in the first seed-mediated synthesis. This can be achieved by precisely controlling the same reaction conditions in both seed-mediated synthesis processes, such as precursor concentration, seed embedding depth in the polymer matrix, and reaction time. The EDX analysis data of these bimetallic and Janus-like nanocrystals (as shown in Supporting Information Figures S1–S10) can be used to calculate the Au/Ag and Pt/Ag concentration ratios in the nanocrystals generated through the second seed-mediated synthesis, as shown in Supporting Information Table S1.

These Janus-like nanocrystals with different compositions and morphologies can be used as catalysts for the reduction of organic dyes. This study investigates the influence of their composition and morphology on catalytic performance. The catalytic performance of Janus-like nanocrystals as a catalyst for the reduction of 4-nitrophenol (4NP, a precursor of commonly used organic dyes in industrial applications) in the presence of sodium borohydride (NaBH_4_) was examined. The catalytic performance of Janus-like nanocrystals for the reduction of 4NP was compared with that of bimetallic nanocrystals with similar morphology and composition in one-half of the Janus-like nanocrystal structure. In the presence of sodium borohydride, 4-nitrophenol (4-NP) undergoes dehydrogenation to generate 4-NP ions, which are then reduced to 4-aminophenol (4AP) on the catalyst surface. The absorption peaks of 4-NP ions and 4-AP in the visible light region are located at ∼400 nm and ∼300 nm, respectively.^10^ The change in the absorption peak of the 4-NP solution at ∼400 nm in the UV–vis spectrum can be used to determine the change in 4-NP concentration in the presence of the catalyst. By measuring the weight of the nanocrystals after removing solvent (ethanol) through heat treatment, the colloidal solution concentration (mg/mL) of bimetallic nanocrystals and Janus-like nanocrystals in ethanol can be determined, as shown in Supporting Information Table S2. The catalytic performance of Janus nanocrystals and bimetallic nanocrystals at the same concentration was compared to demonstrate the enhancing effect of nanocrystal morphology and composition on catalytic performance.

The catalytic properties of Au–Ag flower + Pt–Ag cage nanocrystals at different concentrations were observed by UV–vis spectroscopy, specifically by detecting the change in 4-NP ion concentration in aqueous solution over reaction time, as shown in Supporting Information Figure S19. The relationship between absorption peak intensity (corresponding to the concentration change *I*/*I*_0_, where *I* is the concentration of 4-NP during the reaction and *I*_0_ is the initial concentration of 4-NP) and reaction time conforms to the predictions of a first-order kinetic reaction mechanism ( $I/I_{0}\propto e^{t/t_{0}}$ , t: reaction time and *t*_0_ = 1/reaction constant (K)), as shown in Supporting Information Figure S20A and Table S7. The kinetic reaction mechanisms of these four bimetallic nanocrystals and six Janus-like nanocrystals used as 4-NP reduction catalysts all belong to first-order reaction kinetics. The 4-NP reduction rate (reaction constant K) of Au–Ag flower + Pt–Ag cage nanocrystals as a catalyst increases linearly with the increase of catalyst concentration in the 4-NP aqueous solution, as shown in the blue curve in Figures 2A and S20B. By using Au–Ag flower nanocrystals and Pt–Ag cage nanocrystals as catalysts, a similar relationship between the 4-NP reduction rate and the catalyst concentration can also be measured. The results of using Au–Ag flower nanocrystals as a 4-NP catalyst are shown by the black curve in Figures 2A, S13, and S14 and Table S4. The results of using Pt–Ag cage nanocrystals as a 4-NP catalyst are shown by the red curve in Figure 2A, S15, and S16 and Table S5. Using Au–Ag flower + Pt–Ag cage nanocrystals as 4-NP reduction catalysts, their catalytic performance (K value) is better than the sum of the catalytic performance of Au–Ag flower nanocrystals and Pt–Ag cage nanocrystals as catalysts, and this enhancement trend increases with the increase of 4-NP reduction catalyst concentration. This may be attributed to (1) the percentage of active substances (Au and Pt) in Janus-like nanocrystals being higher than that in bimetallic nanocrystals; (2) the overall structure of Janus-like nanocrystals can provide a higher surface area/volume ratio (morphological effect) and/or synergistic effect (component effect) to improve catalytic performance, while only half of the nanocrystals in bimetallic nanocrystals have one of these two effects to improve catalytic performance; and (3) at the interface between the two structures of the Janus-like nanocrystal, a new synergistic effect formed by Ag, Au, and Pt atoms is generated, which may further enhance the catalytic performance of the Janus-like nanocrystal. The reduction reaction of 4-NP may mainly occur at the interface of the two structures in the nanocrystal, resulting in a significant increase in the reduction rate with the increase of the number of interfaces (catalyst concentration).

**Figure 2:**
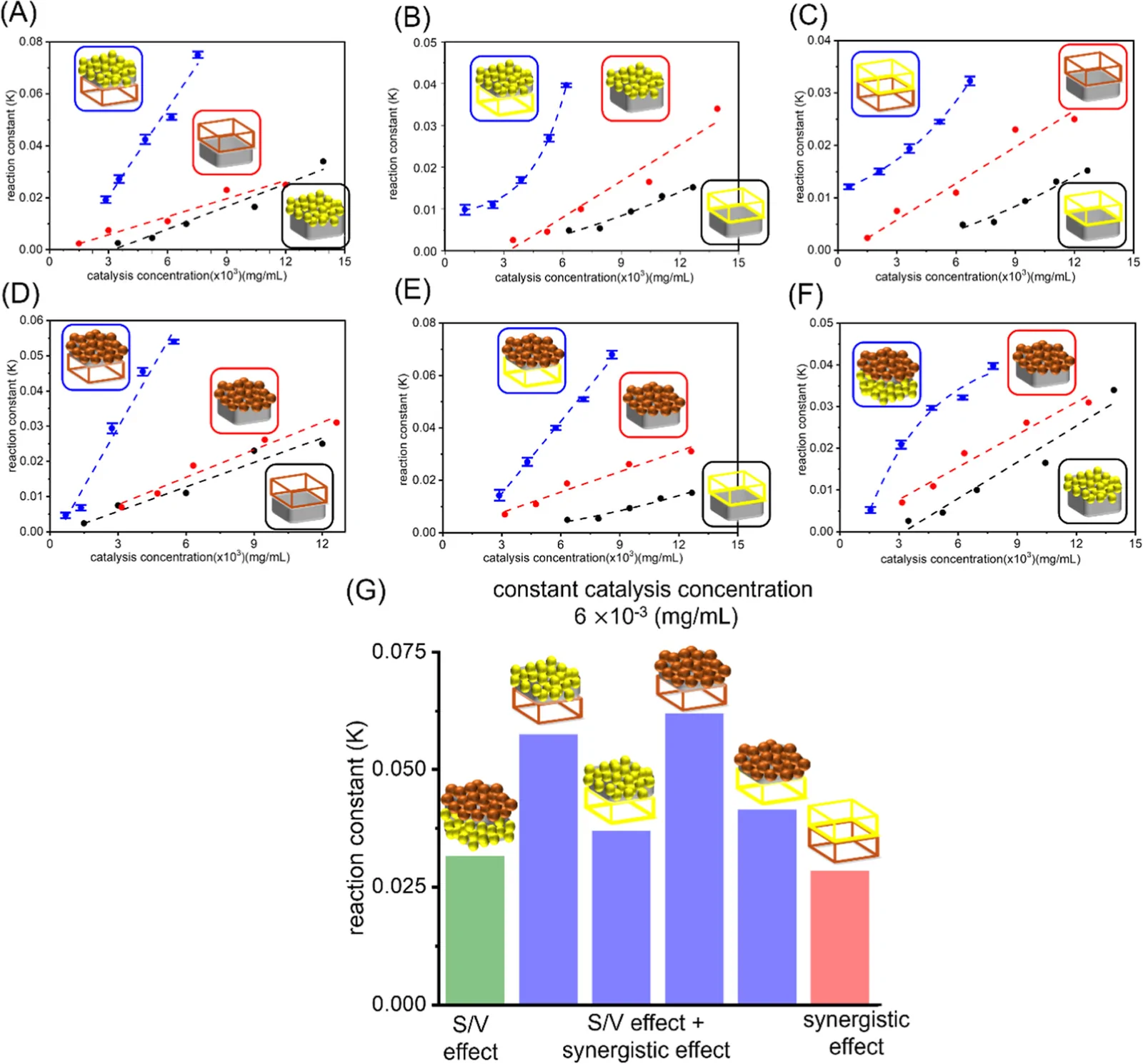
Catalytic performance of Janus-like metallic nanocrystals (two-step continuous seed-mediated products) and corresponding bimetallic nanocrystals (one-step seed-mediated product) on the reduction of 4-nitrophenol (4-NP) was compared. The reaction constant (K) of the 4-NP reduction reaction varies with catalyst concentration: (A) blue curve: Au–Ag flower + Pt–Ag cage nanocrystal; red curve: Pt–Ag cage nanocrystal; and black curve: Au–Ag flower nanocrystal. (B) Blue curve: Au–Ag flower + Au–Ag cage nanocrystal; red curve: Au–Ag flower nanocrystal; and black curve: Au–Ag cage nanocrystal. (C) Blue curve: Au–Ag cage + Pt–Ag cage nanocrystal; red curve: Pt–Ag cage nanocrystal; black curve: Au–Ag cage nanocrystal. (D) Blue curve: Pt–Ag flower + Pt–Ag cage nanocrystal; red curve: Pt–Ag flower nanocrystal; and black curve: Pt–Ag cage nanocrystal. (E) Blue curve: Pt–Ag flower + Au–Ag cage nanocrystal; red curve: Pt–Ag flower nanocrystal; and black curve: Au–Ag cage nanocrystal. (F) Blue curve: Au–Ag flower + Pt–Ag flower nanocrystal; red curve: Pt–Ag flower nanocrystal; and black curve: Au–Ag flower nanocrystal. (G) Catalytic performance of six different Janus-like metallic nanocrystals for the reduction of 4-NP at the same catalyst concentration. The shape effect (S/V ratio) and composition effect (synergistic effect) of Janus-like metallic nanocrystals can be used to enhance catalytic performance. The error bars were obtained from the 4-NP reduction reaction constants over 4 cycles.

These Janus-like nanocrystals composed of Au, Ag, and Pt, as 4-NP reduction catalysts, exhibit superior catalytic performance compared to the sum of the catalytic performance of bimetallic nanocrystals with similar morphology and composition in one-half of the Janus-like nanocrystal structure. This superior performance has also been observed in other Janus-like nanocrystal systems. A comparison of the catalytic performance of Au–Ag cage + Pt–Ag cage nanocrystals as catalysts with that of Au–Ag cage and Pt–Ag cage nanocrystals as catalysts is shown in Figures 2C and S11, S12 and Table S3 (Au–Ag cage), Figures S15 and S16, Table S5 (Pt–Ag cage), and Figures S23 and S24, Table S9 (Au–Ag cage + Pt–Ag cage). However, the enhancement effect of this trend with increasing 4-NP reduction catalyst concentration is similar to that of the Au–Ag flower + Pt–Ag cage system but not as pronounced. This may be because the cage-like structure has fewer active sites than the flower-like structure, resulting in a smaller increase in the number of cage–cage interfaces compared to the flower–cage interface as the number of Janus-like nanocrystals increases. As the catalyst concentration increases, the enhancement of the catalytic performance increases slightly. This may be because the cage-like nanocrystals are lighter than the flower-like nanocrystals and AgNC, resulting in a greater increase in the number of these Janus-like nanocrystals with increasing catalyst concentration (based on the catalyst weight) compared to the increase in the number of bimetallic nanocrystals.

The catalytic performance of other catalyst systems, Pt–Ag-flower + Au–Ag-cage nanocrystals, was compared to that of Pt–Ag-flower and Au–Ag-cage nanocrystals as catalysts. The results are shown in Figures 2E, S17, and S18, Table S6 (Pt–Ag flower), Figures S11 and S12, Table S3 (Au–Ag cage), Figures S27 and S28, and Table S11 (Pt–Ag flower + Au–Ag cage). The catalytic performance of this Janus-like nanocrystal is significantly improved with increasing catalyst concentration compared to the bimetallic nanocrystal, and its catalytic performance is similar to that of the Au–Ag flower + Pt–Ag cage nanocrystal system. This is because these two Janus nanocrystals exhibit similar synergistic effects among Ag, Au, and Pt atoms at the interfaces of the two structures.

Even though Au–Ag flower nanocrystals + Pt–Ag flower nanocrystals are also composed of Ag, Au, and Pt, the synergistic effect between Au, Ag, and Pt at the interface of the two structures is weak because the flower-like nanocrystals are formed by the deposition of smaller Au or Pt nanocrystals generated in aqueous solution onto the seed crystal surface. This results in a significant improvement in the catalytic performance of these Janus-like nanocrystals compared to bimetallic nanocrystals, which is mainly due to the increased surface area per volume ratio of the nanocrystals. However, this improvement in catalytic performance slightly decreases with increasing catalyst concentration, as shown in Figures 2F, S13, and S14, Table S4 (Au–Ag flower), Figures S17 and S18, Table S6 (Pt–Ag flower), Figures S29 and 30, and Table S12 (Au–Ag flower + Pt–Ag flower). As the catalyst concentration increases, the enhancement of catalytic performance decreases slightly. This may be because the flower-like nanocrystals are heavier than the cage-like nanocrystals and AgNC, resulting in a smaller increase in the number of these Janus-like nanocrystals with increasing catalyst concentration (based on the catalyst weight) compared to the increase in the number of bimetallic nanocrystals.

Au–Ag flower + Au–Ag cage nanocrystals, composed of two metals, exhibit superior catalytic performance as a 4-NP reduction catalyst compared to the sum of the performance of Au–Ag cage and Au–Ag flower nanocrystals, as shown in Figure 2B, S11, and S12, Table S3 (Au–Ag cage), Figures S13 and S14, Table S4 (Au–Ag flower), Figures S21 and S22, and Table S8 (Au–Ag cage + Au–Ag flower). This can be attributed to the dual-function nature of these nanocrystals: (1) a high content of active material and (2) both morphological and synergistic effects. The catalytic performance enhancement effect is significantly enhanced with the increase of the Janus-like nanocrystal concentration. This is due to 4-NP reduction preferentially occurring in the Au–Ag flower portion of the Janus-like nanocrystals. This can be seen from Figure 2B, where the catalytic performance of Au–Ag flower nanocrystals (red curve) is higher than that of Au–Ag cage nanocrystals (black curve). When the Janus-like nanocrystal concentration increases, more 4-NP molecules can be reduced on the flower-like structure of the nanocrystal, resulting in significantly enhanced catalytic performance. However, the catalytic performance of Au–Ag cage + Au–Ag flower nanocrystals composed of two metals (as shown by the blue curve in Figure 2B) is lower than that of Janus-like nanocrystals with a similar structure but composed of three metals, namely, Au–Ag flower + Pt–Ag cage nanocrystals (as shown by the blue curve in Figure 2A) and Pt–Ag flower + Au–Ag cage nanocrystals (as shown by the blue curve in Figure 2E). This may be due to the inability to form a synergistic effect among Ag, Au, and Pt atoms at the interface of the two structures in this Janus-like nanocrystal.

Compared with Pt–Ag flower nanocrystals and Pt–Ag cage nanocrystals as catalysts, Janus-like nanocrystals composed of Pt and Ag (Pt–Ag flower + Pt–Ag cage nanocrystal) also exhibited similar catalytic performance enhancement behavior as nanocrystals composed of Au and Ag (Au–Ag flower + Au–Ag cage nanocrystal), as shown in Figures 2D, S17, and S18, Table S6 (Pt–Ag flower), Figures S15 and S16, Table S5 (Pt–Ag cage), Figures S25 and S26, and Table S10 (Pt–Ag flower + Pt–Ag cage). However, the enhanced catalytic performance of these Janus-shaped nanocrystals increases linearly with increasing nanocrystal concentration, which is not similar to the results observed in the Au–Ag flower + Au–Ag cage system. This can be attributed to the fact that 4-NP molecules have similar reducing power on the surfaces of Pt–Ag flower-like structures and Pt–Ag cage-like structures in Janus-like nanocrystals, as shown in Figure 2D, where the catalytic performance of Pt–Ag flower nanocrystals (red curve) is close to that of Pt–Ag cage nanocrystals (black curve).

To compare the catalytic performance of six Janus-like nanocrystals for 4-NP reduction, 4-NP reduction needs to be performed at the same nanocrystal concentration (6 × 10^–3^(mg/mL)). Figure 2G shows the reaction constants for the 4-NP reduction reaction using different Janus-like nanocrystals as catalysts. Based on these results, the following conclusions can be drawn: (1) Janus-like nanocrystals, exhibiting both morphological effects (flower-like nanocrystals) and synergistic effects (cage-like nanocrystals), demonstrate superior catalytic performance compared to those exhibiting only morphological or synergistic effects. (2) The comparison of structurally similar but compositionally different Janus-like nanocrystals indicates that Pt-containing nanocrystals exhibit superior catalytic performance compared to Au-containing nanocrystals. This is because the Pt content in the nanocrystals is higher than the Au content. (To maintain similar framework sizes between Au–Ag and Pt–Ag cages).

These results indicate that Janus-like nanocrystals with highly heterogeneous morphology and composition exhibit superior 4-NP reduction performance compared to relatively low-heterogeneous bimetallic nanocrystals. Under the same reaction conditions, the reaction rate (reaction constant (K)) increased by 1.5 to more than 5 times. This can be attributed to (1) the composition of these Janus-like nanocrystals with highly active materials, whose enhanced catalytic performance stems from the intrinsic properties of the materials themselves and the extrinsic properties (synergistic effect) resulting from the interaction between different metal atoms; and (2) the higher morphological heterogeneity of Janus-like nanocrystals, which provides more active sites for catalytic reactions. Compared with the catalytic and photocatalytic performance of bimetallic nanocrystals Au–Ag and Pt–Ag reported in the literature, Janus-like nanocrystals, with their highly heterogeneous morphology and composition, can degrade 4 to more than 10^2^ times more 4-NP molecules per unit weight of nanocrystal within a similar reaction time when used as catalysts or photocatalysts, as shown in Supporting Information Table S13.^20–24^

Nanocrystals composed of superior plasmonic materials can interact with radiation at specific wavelengths, generating high-energy electrons (hot electrons) that oscillate around the nanocrystal. These hot electrons can convert the photon energy into chemical energy to accelerate chemical reactions. Therefore, Janus-like nanocrystals composed of Ag and/or Au (which possess excellent plasmonic properties) are well-suited for use as photocatalysts in various chemical reactions such as the reduction of organic dyes.

Here, three Janus-like nanocrystals (Pt–Ag flower + Pt–Ag cage, Au–Ag flower + Au–Ag cage, and Au–Ag flower + Pt–Ag flower) were used to study the effect of nanocrystal composition on the photocatalytic performance of 4-NP reduction. The photocatalytic performance of these Janus-like nanocrystals on the reduction of 4-NP was tested at two different irradiation wavelengths: (1) one close to the plasmonic resonance peak (on plasmon mode, plasmon-induced hot electrons + photothermal effects); and (2) the other far from the plasmonic resonance peak (off-plasmon mode, mainly from photothermal effects). Compared to catalytic performance without irradiation, the photocatalytic performance enhancement factor ( $EF=\frac{K\;under\;irradation-Kwithout\;irradaition}{Kwithout\;irradaition}$ ) of Janus-type nanocrystals was used to represent the effectiveness of plasmonic effects in enhancing the reduction of 4-NP.

For the Pt–Ag flower + Pt–Ag cage photocatalyst, two irradiation wavelengths were selected based on the extinction spectrum in the Supporting Information Figure S8A: 590 nm (on-plasmon mode) and 465 nm (off-plasmon mode) to study the photocatalytic performance of 4-NP reduction. The mechanism by which this Janus-like nanocrystal acts as a photocatalyst or catalyst remains unchanged, both belonging to first-order kinetic reactions, as shown in Supporting Information Figures S35 and S36 and Table S16 (reaction in on-plasmon mode); Figures S37 and S38 and Table S17 (reaction in off-plasmon mode). Figure 3A records the change of the reaction constant (K) under irradiated and nonirradiated conditions when the concentration of Janus-like nanocrystals changes. The results show that the enhancement factor (EF) of photocatalytic performance is about 0.16 under off-plasmon-mode (465 nm) irradiation and about 0.24 under plasmon-mode (590 nm) irradiation. This low EF is due to the intrinsic inability of Pt to provide a strong plasmonic response, resulting in a significant decrease in extinction peak intensity compared to AgNC, as shown in the Supporting Information Figure S8A.

**Figure 3:**
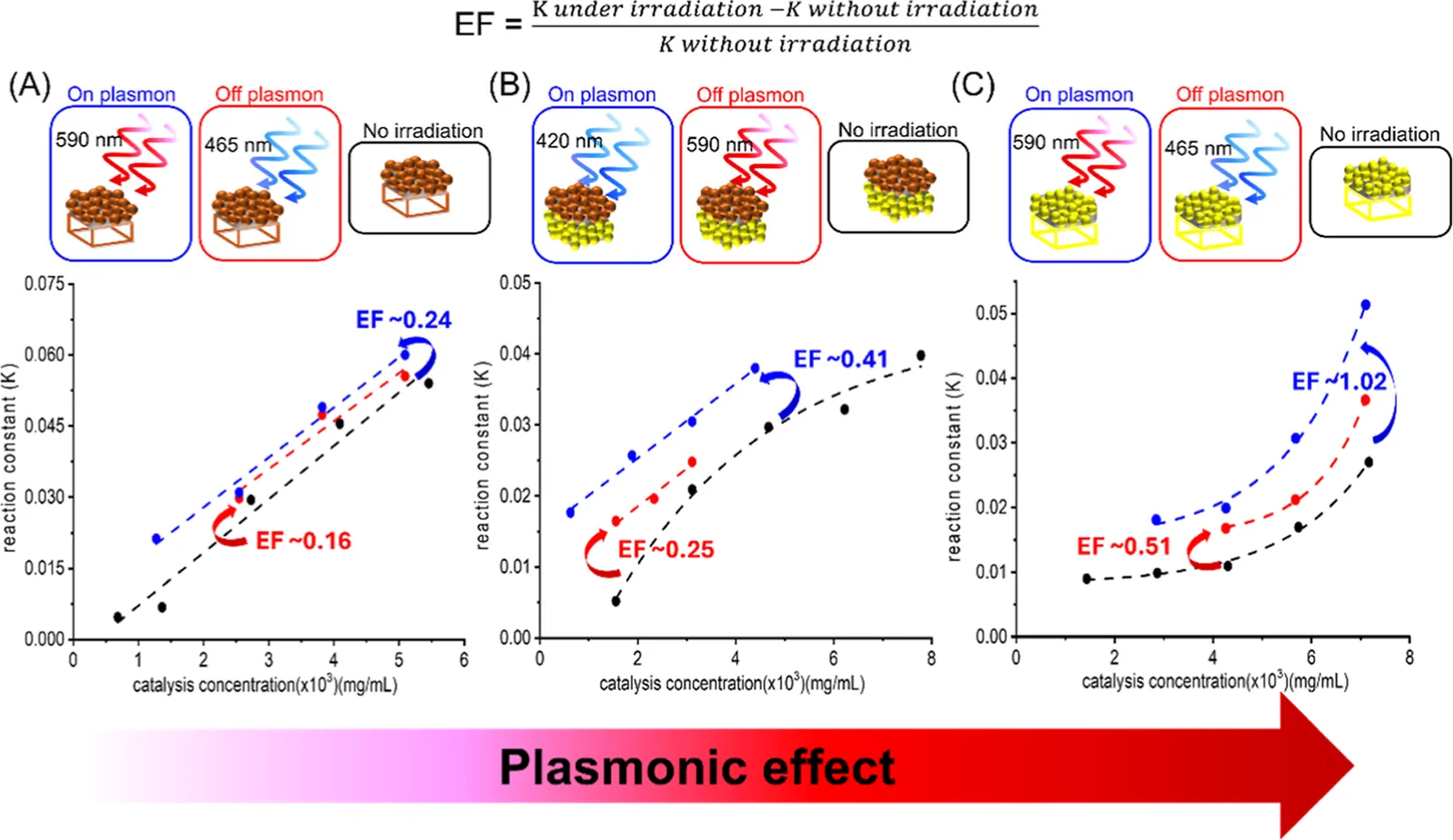
Janus-like metallic nanocrystals were used as photocatalysts for the 4-NP reduction reaction, and their photocatalytic performance under different wavelengths of light irradiation was investigated. The photocatalytic performance can be represented by the enhancement factor (EF) of the reaction constant (*K*) under irradiation and without irradiation. The enhancement factor of *K* = (*K* under irradiation – *K* without irradiation)/*K* without irradiation. (A) Pt–Ag flower + Pt–Ag cage nanocrystal: black curve: without irradiation; red curve: irradiation at off-plasmonic mode wavelength (465 nm); and blue curve: irradiation at on-plasmonic mode wavelength (590 nm). (B) Pt–Ag flower + Au–Ag flower nanocrystal: black curve: without irradiation; red curve: irradiation at off-plasmonic mode wavelength (590 nm); and blue curve: irradiation at on-plasmonic mode wavelength (420 nm). (C) Au–Ag flower + Au–Ag cage nanocrystal: black curve: without irradiation; red curve: irradiation at off-plasmonic mode wavelength (465 nm); and blue curve: irradiation at on-plasmonic mode wavelength (590 nm).

Using Janus-like nanocrystals (Au–Ag flower + Au–Ag cage nanocrystals), composed of excellent plasmonic materials Ag and Au, as photocatalysts for 4-NP reduction can significantly enhance the plasmonic effect. Based on the extinction spectrum of Au–Ag flower + Au–Ag cage nanocrystals shown in Supporting Information Figure S6A, irradiation wavelengths of 590 nm (on-plasmon mode) close to the plasmonic peak and 465 nm (off-plasmon mode) far from the plasmonic peak were selected for the photocatalytic reduction reaction. Compared to the catalytic performance without irradiation, the photocatalytic performance enhancement factor of this Janus-like nanocrystal is significantly better than that of the Janus-like nanocrystal composed of Pt and Ag systems, as shown in Figure 3C. When irradiated in on-plasmon excitation mode (590 nm), the enhancement factor (EF) is approximately 1.02; when irradiated in off-plasmon excitation mode (465 nm), the enhancement factor (EF) is approximately 0.51, as shown in Supporting Information Figures S31 and S32 and Table S14 (reaction in on-plasmon mode) and Figures S33 and S34 and Table S15 (reaction in off-plasmon mode).

The photocatalytic performance enhanced by the plasmonic effect of Janus-like nanocrystals composed of Ag, Au, and Pt (Au–Ag flower + Pt–Ag flower nanocrystals) should be higher than that of Janus-like nanocrystals composed of Pt and Ag but lower than that of Janus-like nanocrystals composed of Au and Ag. By examining the extinction spectrum of Au–Ag flower + Pt–Ag flower nanocrystals, as shown in the Supporting Information Figure S10A, the appropriate irradiation wavelength for studying the photocatalytic performance of this nanocrystal can be selected. The wavelength close to the plasmon mode is 420 nm (on-plasmon mode), and the wavelength far from the plasmon mode is 590 nm (off-plasmon mode). As shown in Figure 3B, the photocatalytic performance enhancement factor (EF) of this nanocrystal in on-plasmon mode is approximately 0.41, and the photocatalytic performance enhancement factor (EF) in off-plasmon mode is approximately 0.25, as shown in Supporting Information Figures S39 and S40 and Table S18 (reaction in on-plasmon mode); Figures S41 and S42 and Table S19 (reaction in off-plasmon mode).

These results confirm that introducing a plasmonic effect into the catalytic process can further enhance the catalytic performance of the Janus-like nanocrystals. Compared to nonirradiated catalytic performance, the enhancement factor of photocatalytic performance strongly depends on the composition of the Janus-like nanocrystals. The higher the content of plasmonic material in the nanocrystals, the higher the enhancement factor of the photocatalytic performance.

When inorganic nanocrystals are used as catalysts, in addition to their catalytic performance, the stability of the catalyst during the chemical reaction process also plays an important role and, therefore, needs to be considered. Here, Au–Ag flower and Au–Ag cage nanocrystals were selected to investigate the stability of Janus-like nanocrystals as catalysts and photocatalysts. These Janus-like nanocrystals exhibited the best photocatalytic performance, indicating that a vigorous chemical reaction occurred on the nanocrystal surface. Furthermore, the photocatalytic reaction proceeded under more stringent conditions than reactions under no-light conditions (which are more likely to cause erosion of the nanocrystal surface): the conversion of photon energy to electron energy in photocatalytic reactions makes them more prone to corrosion of the nanocrystal surface. This Janus-like nanocrystal was used as a photocatalyst for the reduction of 4-NPs under irradiation at a wavelength of 590 nm (on-plasmon-resonance mode of this nanocrystal). This photoreduction reaction was repeated four times, and the photocatalytic performance and morphology of the nanocrystals were analyzed to characterize their stability as photocatalysts. The photocatalytic performance was obtained by analyzing the changes in the 4-NP absorption intensity in the UV–vis spectrum during the reaction, as shown in Figures 4A and S43. The results showed that after four cycles of reaction, the photocatalytic performance (reaction constant) decreased by only 14%, and the chemical reaction mechanism remained unchanged (belonging to first-order kinetics), as shown in Figure 4B and Table S20. Before the reaction and after four cycles of photoreduction, the TEM images of this Janus-like nanocrystal showed that its morphology remained unchanged, as shown in Figure 4C. EDX analysis of Janus nanocrystals before 4-NP reduction (as shown in Supporting Information S6B) and after four 4-NP reductions (as shown in Supporting Information S45) showed that the composition of Janus nanocrystals remained almost unchanged. These results indicate that the stability of Janus-like nanocrystals as photocatalysts is sufficient to meet the requirements for their repeated use.

**Figure 4:**
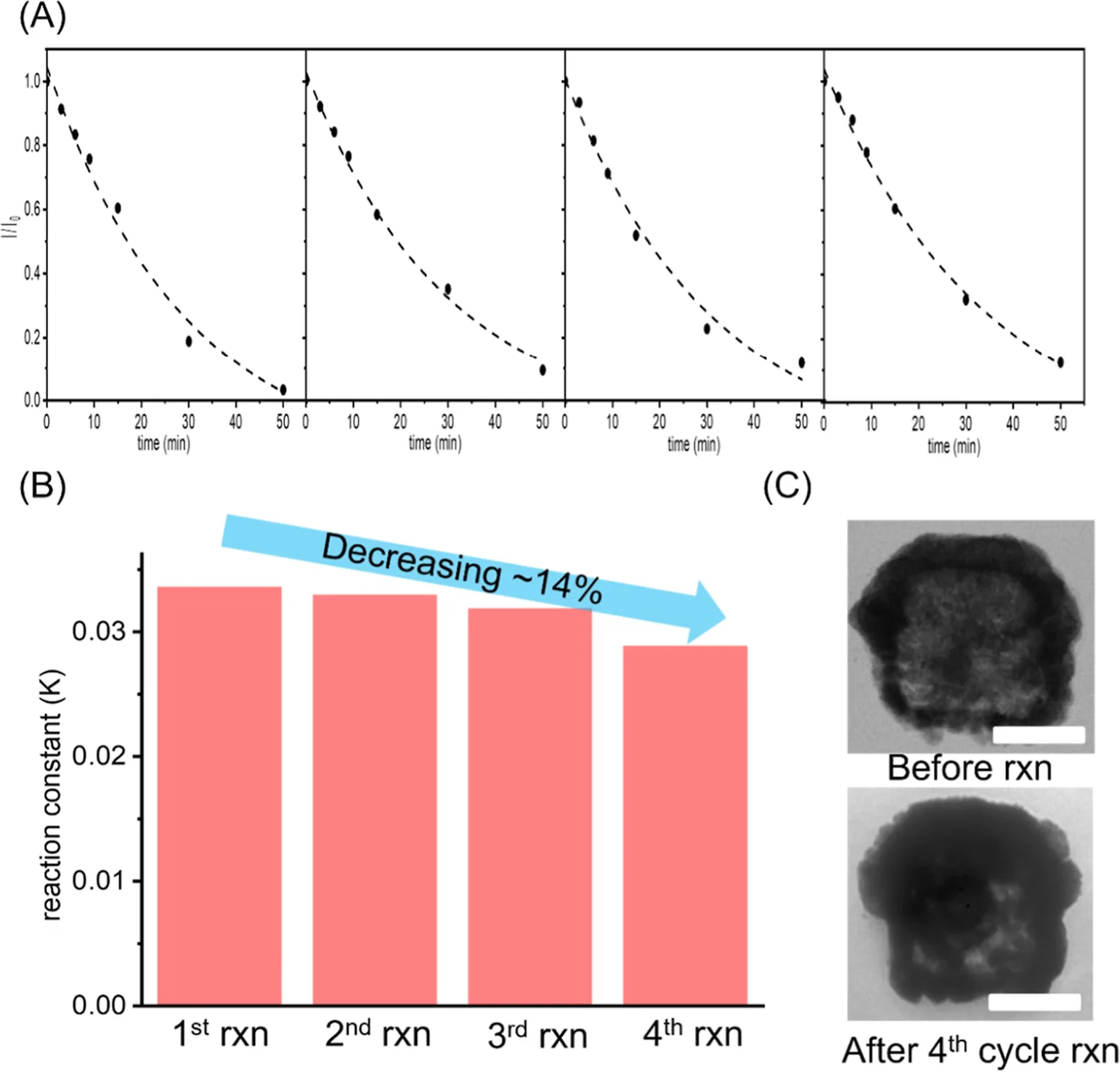
Stability testing of Au–Ag flower-like + Au–Ag cage nanocrystals as photocatalysts for the reduction reaction of 4-nitrophenol (4-NP) under the plasmonic mode of this nanocrystal (590 nm). (A) Variation of UV–vis absorption intensity (*I*/*I*_0_) of 4-NP with reaction time during 1 to 4 reaction cycles. (B) Variation of reaction constant (*K*) of the 4-NP reduction reaction with the number of reaction cycles. (C) Transmission electron microscopy (TEM) images before reaction and after four reaction cycles confirm that the morphology of Au–Ag flower-like + Au–Ag cage-like nanocrystals remain unchanged. The scale bar is 50 nm.

To demonstrate that the use of Janus-like nanocrystals as photocatalysts in chemical reactions is not specifically for 4-NP reduction, Janus-like nanocrystals have also been used as catalysts and photocatalysts to degrade other common industrial organic dyes, such as methyl orange (MO), Congo red (CR), and Rhodamine 6G (R6G). In the presence of sodium borohydride (NaBH_4_), the degradation of these organic dyes was catalyzed by Au–Ag flower + Au–Ag cage nanocrystals as catalysts (without light) and a photocatalyst (590 nm, corresponding to the on-plasmon mode of the nanocrystals). The degradation reactions of MO, CR, and R6G under the presence of NaBH_4_, and the relative absorption peaks before and after degradation, as shown in Figure 5B–D, respectively.^25–28^ By observing the changes in absorption intensity of N=N groups in MO at a wavelength of 480 nm, the changes in absorption intensity of N=N groups in CR at a wavelength of 500 nm, and the changes in absorption intensity of ^+^N=C groups in R6G at a wavelength of 560 nm, the concentration changes of MO, CR, and R6G during the degradation process can be obtained. The relevant UV–vis spectral changes during the degradation process are shown in Supporting Information Figure S44.

**Figure 5:**
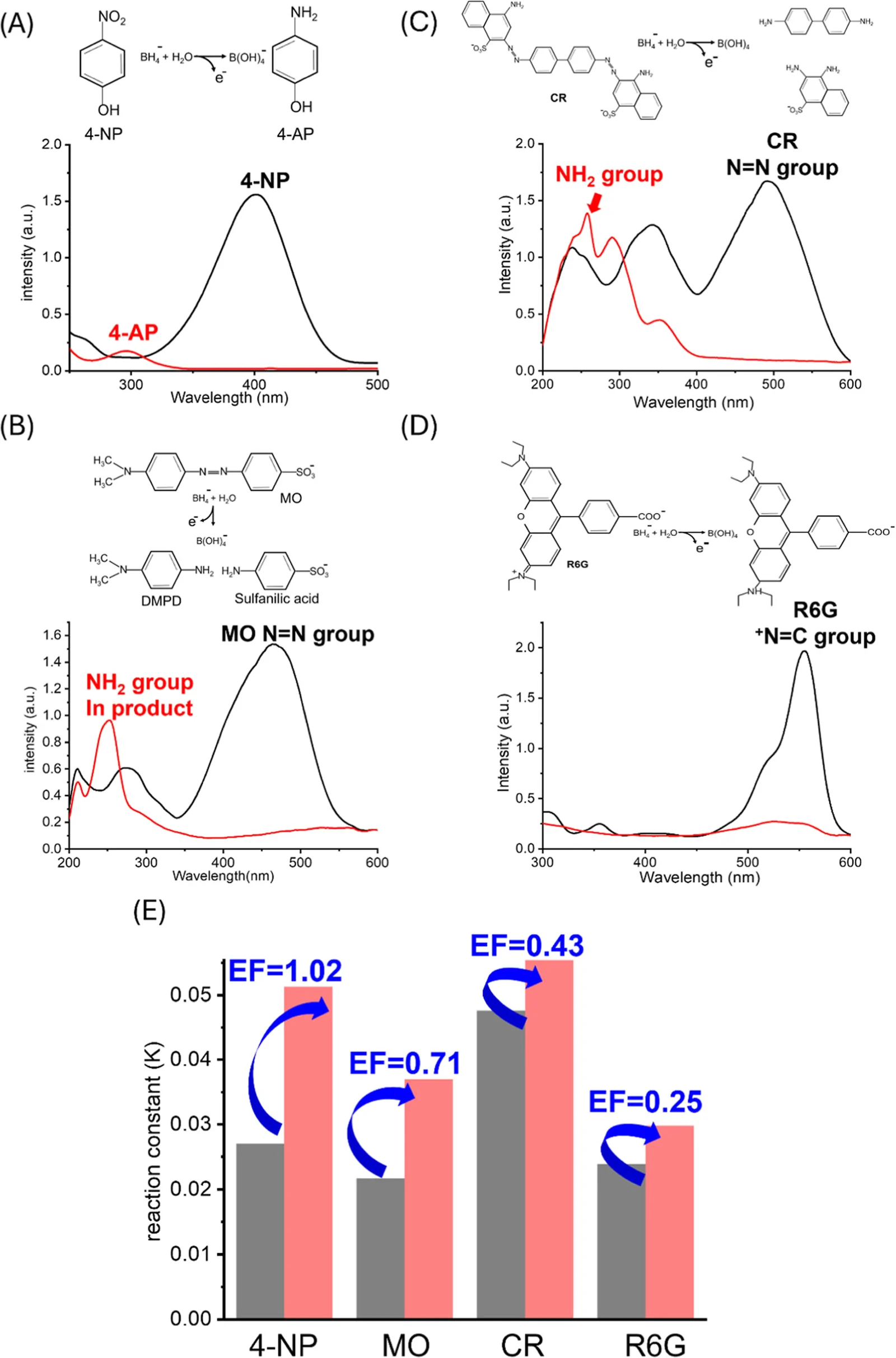
Reduction reactions of 4-NP and three different organic dyes were studied using Au–Ag flower-like + Au–Ag cage nanocrystals as catalysts and photocatalysts. (A) Changes in the UV–vis absorption curves of 4-nitrophenol (4-NP) before and after the reduction reaction. (B) Changes in the UV–vis absorption curves of methyl orange (MO) before and after the reduction reaction. (C) Changes in the UV–vis absorption curves of Congo red (CR) before and after the reduction reaction. (D) Changes in the UV–vis absorption curves of Rhodamine 6G (R6G) before and after the reduction reaction. Black curve: before reaction; red curve: after reaction. (E) Changes in the reaction constant (*K*) for the reduction of 4-NP and organic dyes using Au–Ag flower-like + Au–Ag cage nanocrystals as catalysts and photocatalysts.

And the degradation mechanisms of all organic dyes conformed to first-order kinetic reaction predictions, as shown in Supporting Information Figure S44 and Table S21. In the presence of Janus-like nanocrystals as catalysts or photocatalysts, all organic dyes underwent degradation, with reaction constants of approximately 0.02–0.055 (min^–1^). The enhancement factor (EF) for photocatalytic performance was 0.25–1.02 compared to catalytic performance, as shown in Figure 5E. Organic dyes with a main absorption peak closer to the irradiation wavelength (590 nm) exhibited a lower EF. This may be because the organic dyes absorbed some of the irradiation photons, reducing the number of photons absorbed by the nanocrystals and thus affecting the photoreduction reaction. These results suggest that by adjusting the plasmonic response of the nanocrystals, it may be possible to optimize the degradation of different organic dyes by Janus-like nanocrystals as photocatalysts.

## 3 Conclusions

Through the “two-step continuous seed-mediated” method, highly heterogeneous Janus-like nanocrystals composed of active materials (Au and Pt) and plasmonic materials (Ag and Au) with tunable morphology and composition can be obtained. Compared to bimetallic nanocrystals with half of the Janus-like nanocrystal structure, these Janus-like nanocrystals exhibit significantly enhanced catalytic performance for the reduction of 4-NP and organic dyes, increasing by approximately 1.5 to 5 times. This can be attributed to the fact that its highly heterogeneous morphology and composition provide a greater number of active sites (higher surface area/volume ratio) and higher active sites (strong synergistic effect) for the reaction. Since these Janus-like nanocrystals are composed of Ag and Au, which have excellent plasmonic properties, their catalytic performance in reduction reactions can be further enhanced under irradiation at specific wavelengths. Compared with the catalytic and photocatalytic performance of bimetallic nanocrystals Au–Ag and Pt–Ag reported in the literature, Janus-like nanocrystals can degrade 4 to more than 10^2^ times more 4-NP molecules per unit weight of nanocrystal within a similar reaction time. These results demonstrate that Janus-like nanocrystals exhibit excellent catalytic and photocatalytic performance in the reduction of 4-NP and organic dyes. The photocatalytic performance also depends on the number of photons that the nanocrystals can effectively absorb, and photon absorption by organic dyes inhibits this effect. Furthermore, these nanocrystals also exhibit high stability under more stringent multicycle (more than 4 cycles) reaction conditions. These results demonstrate that by adjusting the structure and composition of nanocrystals, specific chemical reactions can be accelerated, making the design of multifunctional nanocrystals through ″multistep continuous seed-mediated″ methods valuable.

## 4 Experimental Section

### 4.1 Materials

Silver nitrate (≥99%, Fluka), 1,5-pentanediol (98%, ACROS), polyvinylpyrrolidone (PVP, *M*_w_ = 55,000 (g/mol), Sigma), copper­(II) chloride (99%, Sigma), polystyrene (PS, *M*_w_ = 35,000 (g/mol), Sigma), hydrogen tetrachloroaurate (III) trihydrate (99.99%, Alfa Aesar), potassium Tetrachloroplatinate­(II) (99.9%, UniRegion Bio-Tech), sylgard 184 (silicone elastomer, prepolydimethylsiloxane­(PDMS) solution, UniRegion Bio-Tech), l­(+)-Ascorbic acid (99%, ACROS), ethanol (95%), toluene (>99.5%, J.T. Baker), and chloroform (99.8%, Macron) 4-Nitrophenol­(99%, Alfa Aesar), congo red­(Dye content ≥35%, Sigma), methyl orange­(Pure, ACROS), and rhodamine 6G­(95%, Sigma) were used.

### 4.2 Synthesis of Shaped AgNCs

Silver nanocubes (AgNCs) were synthesized via a modified polyol process involving two precursor solutions prepared in 5 mL of 1,5-pentanediol: the first one contained 0.20 g of AgNO3 and 44 μL of 0.043 M CuCl2, the second one consisted of 0.10 g of PVP. The synthesis was performed by heating 10 mL of 1,5-pentanediol to 193 °C in a 50 mL round-bottom flask under continuous stirring. The AgNO3 and PVP precursors were then alternately injected into the hot solvent at rates of 500 μL/min and 320 μL/30 s, respectively, with the final particle size determined by the total injection volume. To achieve high morphological uniformity, the crude colloidal solution—which initially contained approximately 20% shape impurities such as triangular, spherical, and wire-like nanocrystals, underwent a rigorous postsynthetic purification protocol. After the synthesis process, sequential filtration four times with 450 nm filter paper and six times with 220 nm filter paper was performed, and the population of uniform AgNCs increased to over 95%. These purified AgNCs, characterized by an average size of 100 nm, were subsequently employed as seeds for the fabrication of bimetallic (seed-mediated products) and Janus-like metallic nanocrystals (“two-step continuous seed-mediated” products).

### 4.3 Deposition of Polystyrene Thin Films on the Substrate

Silicon or glass substrates were first cleaned by using a freshly prepared piranha solution, consisting of concentrated sulfuric acid and hydrogen peroxide with a 7:3 volume ratio. To enhance the adhesion between the substrate and the subsequent polymer layer, the surfaces were treated with hexamethyldisilazane (HMDS) vapor to induce hydrophobicity. Polystyrene (PS) was dissolved in toluene at a concentration of approximately 5 wt % and deposited onto the HMDS-treated substrates via spin-coating. The thickness of the resulting PS films was precisely regulated by adjusting the polymer solution concentration and the spin speed; in this study, the film thickness was maintained at approximately 200 nm.

### 4.4 Deposition of AgNC Arrays on PS Films and Partial Embedded AgNC into PS Films

To prepare the AgNC arrays on PS films, the purified AgNC colloidal solution was first precipitated in ethanol and subsequently redispersed in CHCl3 to accelerate the evaporation rate during array formation. A Langmuir–Blodgett (LB) technique was employed, where the AgNC/CHCl3 suspension was added dropwise to the water–air interface within a glass Petri dish, resulting in an isotropically distributed monolayer of silver nanocrystals. The surface tension at the interface was modulated to control the AgNC surface coverage and packing density. Finally, the well-arrayed AgNC monolayer was transferred onto the PS-coated substrates via a dip-coating process, ensuring a uniform distribution across the polymer thin film. The AgNC array on the PS thin film was then partially embedded into the PS film by thermal treatment at a temperature higher than the glass-transition temperature of PS (*T* > *T*_g_ of PS). The embedding depth of AgNCs in PS film can be controlled by thermal treatment conditions (treatment temperature and time).

### 4.5 Using AgNC Exposure Height ∼ 2*L*/3 in a Polymer Matrix as a Template to Synthesize Heterogeneous Au–Ag Nanocrystals or Pt–Ag Nanocrystals through a First “Seed-Mediated” Method.^11^

To embed the uniform AgNC arrays into the polystyrene (PS) film, we subjected the AgNC-decorated substrates (silicon or glass) to a thermal treatment process. This thermal treatment allowed the AgNCs to partially sink into the 200 nm thick PS layer until a specific exposure height (H) of approximately 2*L*/3 (66 nm) was reached, where L represents the edge length of the nanocubes. The resulting AgNC-PS composite substrate was then suspended in various aqueous reaction media to facilitate further chemical modifications under controlled conditions.

For synthesis of Au–Ag nanocrystals: (1) various PVP (reducing agents and ligands) and (2) Au^3+^/Ag atoms (reactive sites).

The different concentration of HAuCl_4_ solution as a Au^3+^ ion precursor with a specific volume was injected into the reaction solution for the formation of heterogeneous Au–Ag nanocrystals by reducing the Au^3+^ ion. Due to the different reaction conditions, there are two different heterogeneous Pt–Ag nanocrystals: flower-like and cage-like nanocrystals can be generated. The details of the synthesis process were shown as follows.

(a) Flowerlike nanocrystals: 8 mg of PVP was dissolved in 12 mL of deionized water (0.012 mM) within a 20 mL glass vial. The AgNC-PS-glass or AgNC-PS-silicon substrates were mounted and suspended in this PVP solution under constant magnetic stirring. The reaction was conducted both under high-definition (HD) mode irradiation and in the absence of irradiation to evaluate the effects of light. Subsequently, 1.37 mL of a 1 mM AuCl_4_^–^ solution (maintaining a molar ratio of Au^3+^ ions to exposed surface silver atoms = 2.75) was injected at 20 min intervals. The time-dependent optical evolution of the samples was monitored via extinction spectroscopy of the AgNC-PS-glass substrates, while the morphological formation of the Au–Ag alloy nanocrystals was characterized by using scanning electron microscopy (SEM) and high-resolution transmission electron microscopy (HRTEM).
(b) Cage-like nanocrystals: 8 mg of PVP was dissolved in 12 mL of deionized water (0.0125 mM) within a 20 mL glass vial. The AgNC-PS-glass and AgNC-PS-silicon substrates were mounted and suspended in this aqueous PVP solution under continuous magnetic stirring. The reaction was conducted both under high-definition (HD) mode irradiation and in the absence of irradiation to evaluate the influence of photoinduction. Subsequently, 3.72 mL of a 2.1 mM AuCl_4_^–^ solution (maintaining a molar ratio of Au^3+^ ions to exposed surface silver atoms = 7.5) was injected at 20 min intervals. The time-dependent optical evolution of the samples was monitored via extinction spectroscopy of the AgNC-PS-glass substrates, while the morphological transformation of the Au–Ag alloy nanocrystals was characterized by using scanning electron microscopy (SEM) and high-resolution transmission electron microscopy (HRTEM).

For synthesis of Pt–Ag nanocrystals: (1) various PVP (reducing agents and ligands) and AA concentrations (reducing agents); (2) reaction temperatures; and (3) Pt^2+^/Ag atoms (reactive sites).

The different concentration of K_2_PtCl_4_ solution as a Pt^2+^ ion precursor with a specific volume was injected into the reaction solution for the formation of heterogeneous Pt–Ag nanocrystals by reducing the Pt^2+^ ion. Due to the different reaction conditions, there are two different heterogeneous Pt–Ag nanocrystals: flower-like and cage-like nanocrystals can be generated. The detail synthesis process shows as follows.

(a) Flower-like nanocrystals: 10 mg of PVP and 1 mg of ascorbic acid (AA) were dissolved in 15 mL of deionized water (with concentrations of 0.012 mM and 0.388 mM, respectively) within a 20 mL glass vial at room temperature. The AgNC-PS-glass and AgNC-PS-silicon substrates were subsequently mounted and suspended in this PVP/AA aqueous solution under continuous magnetic stirring. Following this, 21.2 mL of a 1 mM PtCl_4_^–2^ solution (maintaining a molar ratio of Pt^2+^ ions to exposed surface silver atoms = 10) was injected at 20 min intervals. The time-dependent optical evolution of the samples was monitored via extinction spectroscopy of the AgNC-PS-glass substrates, while the morphological transformation of the Pt–Ag alloy nanocrystals was characterized by using scanning electron microscopy (SEM) and high-resolution transmission electron microscopy (HRTEM).

PS: the extinction spectrum intensity of the AgNC array = 1.154 was used to calculate the number of AgNCs in the substrate and the volume of Pt^2+^ ion solution injected for the reaction.

(b) Cage-like nanocrystal: 10 mg of PVP was dissolved in 15 mL of deionized water (0.012 mM) within a 20 mL glass vial maintained at 0 °C. The AgNC-PS-glass and AgNC-PS-silicon substrates were mounted and suspended in this chilled PVP aqueous solution under continuous magnetic stirring. Subsequently, 14.1 mL of a 0.1 M PtCl_4_^–2^ solution (maintaining a molar ratio of Pt^2+^ ions to exposed surface silver atoms of 2000) was injected at 20 min intervals. The time-dependent optical evolution of the samples was monitored via extinction spectroscopy of the AgNC-PS-glass substrates, while the morphological transformation of the Pt–Ag nanocrystals was characterized by using scanning electron microscopy (SEM) and high-resolution transmission electron microscopy (HRTEM).

PS: The extinction spectrum intensity of the AgNC array = 0.7 was used to calculate the number of AgNCs in the substrate and the volume of Pt^2+^ ion solution injected for the reaction.

### 4.6 Reversal of Heterogeneous Bimetallic Nanocrystals Embedding the PS Matrix into the PDMS Matrix

To fabricate Janus-like nanocrystals, a pre-PDMS solution, which consists of 90 wt % dimethylsiloxane and 10 wt % curing agent, was deposited onto the PS-supported heterogeneous Au–Ag or Pt–Ag nanocrystal arrays via drop-coating. A clean silicon or glass substrate was then placed on top to create a sandwich structure consisting of PDMS, the heterogeneous nanocrystals, and the PS layer. This assembly structure was subjected to thermal treatment in an oven at 50 °C for 2 days to facilitate the polymerization of the dimethylsiloxane into a solid PDMS film.

Subsequently, the sandwich structure was immersed in an acetone bath at 0 °C for 24 h to selectively dissolve the PS layer. This dissolution process successfully exposed the previously embedded, unreacted silver part of the heterogeneous nanocrystals, which now protruded from the PDMS matrix. These newly accessible surfaces served as specific nucleation sites for a secondary “seed-mediated” growth phase, facilitating the fabrication of Janus-like nanocrystals with tailored morphologies and multifaceted chemical compositions.

The reaction conditions of the second “seed-mediated” method were the same as the first “seed-mediated” method, and Au–Ag or Pt–Ag nanocrystals with different morphologies can be formed on the unreacted AgNC part in the heterogeneous nanocrystals.

### 4.7 Removal of PDMS for Janus Nanocrystal Characterizations

The PDMS with Janus nanocrystals was immersed in chloroform and shaken in an ultrasonic water bath for 20–30 min to dissolve the PDMS. Janus nanocrystals were separated from a chloroform solution containing PDMS and Janus nanocrystals through centrifugation at a centrifugal speed of 13.4 krpm for 20 min. The precipitation generated after centrifugation was redispersed in 0.5 mL of ethanol and then centrifuged to remove traces of PDMS in the solution. The redispersion and centrifugation process of Janus nanocrystals in ethanol were repeated twice before further characterization of Janus nanocrystals.

### 4.8 Janus-like Metallic Nanocrystals and Bimetallic Nanocrystals as Catalysts and Photocatalysts for the Reduction of Organic Dyes

#### 4.8.1 4-Nitrophenol Reduction Reaction

The synthesized Janus or bimetallic nanocrystals are placed in a glass vial with 2 mL of chloroform and subjected to ultrasonication for 20–30 min to detach the nanocrystals from the PDMS surface. After that, the suspension is centrifuged at 13.4 krpm for 20 min, then the supernatant is removed. To ensure complete elimination of residual PDMS, the precipitates are washed with 1 mL of ethanol and centrifuged at 13.4 krpm for 20 min. This process was repeated three times. After the final centrifugation, the nanocrystals are resuspended in 1 mL of ethanol via ultrasonication to achieve a uniform dispersion.

For the degradation of the organic dye, a 1.7 mM 4-nitrophenol (4-NP) solution and a 0.4 M sodium borohydride (NaBH_4_) solution are prepared. In a cuvette, 2.6 mL of deionized water is mixed with 150 μL of the 1.7 mM 4-NP solution and 150 μL of the 0.4 M NaBH4 solution; the mixture turns yellow upon addition, at which point the UV–vis extinction spectrum (200–600 nm) is recorded as T0. Finally, 100 μL of the nanocrystal suspension is added to the cuvette. The UV–vis spectrum is measured at specific intervals (3, 6, 9, 15, 30, and 60 min, followed by hourly measurements) until the extinction peaks show no further significant changes.

For the photocatalytic performance tests, the solution was irradiated with visible light at wavelengths corresponding to the specific extinction spectral modes of the Janus nanocrystals. This targeted irradiation is designed to enhance the localized surface plasmon resonance (LSPR) effect. The light source used for photocatalytic performance measurement was an LED point light source with its power at 9.9 W. The UV–vis spectrum is measured at specific intervals (3, 6, 9, 15, 30, and 60 min, followed by hourly measurements) until the extinction peaks show no further significant changes.

The catalytic and photocatalytic performance of 4-NP and organic dye reductions is detected by observing the changes in the UV–vis absorption peaks corresponding to the dye molecules over time.

The reaction mechanism: a 4NP molecule forms 4NP ions in the presence of NaBH_4_ and then the 4NP ions and BH_4_^–^ ions react on a catalyst or photocatalyst (nanocrystals) to generate 4AP.

#### 4.8.2 Methyl Orange Reduction Reaction

A 15 mM methyl orange (MO) aqueous solution and a 0.4 M sodium borohydride (NaBH_4_) solution were prepared. In a cuvette, 2650 μL of DI water is mixed with 100 μL of 15 mM MO solution and 150 μL of the 0.4 M NaBH4, at which point the UV–vis extinction spectrum (200–600 nm) is recorded as T0. Finally, 100 μL of the nanocrystal suspension is added to the cuvette. The UV–vis spectrum is measured at specific intervals (3, 6, 9, 15, 30, and 60 min, followed by hourly measurements) until the extinction peaks show no further significant changes.

For the photocatalytic performance tests, the solution was irradiated with visible light at wavelengths corresponding to the specific extinction spectral modes of the Janus nanocrystals. This targeted irradiation is designed to enhance the localized surface plasmon resonance (LSPR) effect. The light source used for photocatalytic performance measurement was an LED point light source with its power at 9.9 W. The UV–vis spectrum is measured at specific intervals (3, 6, 9, 15, 30, and 60 min, followed by hourly measurements) until the extinction peaks show no further significant changes.

The reaction mechanism: the methyl orange molecule and BH_4_^–^ ions react with a catalyst and a photocatalyst (nanocrystal), and then the azo group on the methyl orange is hydrogenated and reduced, cleaving into an aromatic amine.

#### 4.8.3 Congo Red Reduction Reaction

A 1 mM Congo Red (CR) aqueous solution and a 0.4 M sodium borohydride (NaBH_4_) solution were prepared. In a cuvette, 2650 μL of DI water is mixed with 100 μL of 1 mM CR solution and 150 μL of the 0.4 M NaBH_4_, at which point the UV–vis extinction spectrum (200–600 nm) is recorded as *T*_0_. Finally, 100 μL of the nanocrystal suspension is added to the cuvette. The UV–vis spectrum is measured at specific intervals (3, 6, 9, 15, 30, and 60 min, followed by hourly measurements) until the extinction peaks show no further significant changes.

For the photocatalytic performance tests, the solution was irradiated with visible light at wavelengths corresponding to the specific extinction spectral modes of the Janus nanocrystals. This targeted irradiation is designed to enhance the localized surface plasmon resonance (LSPR) effect. The light source used for photocatalytic performance measurement was an LED point light source with its power at 9.9 W. The UV–vis spectrum is measured at specific intervals (3, 6, 9, 15, 30, and 60 min, followed by hourly measurements) until the extinction peaks show no further significant changes.

The reaction mechanism: congo red molecules and BH_4_^–^ ion react with a catalyst and a photocatalyst (nanocrystals), and then the azo groups on Congo red are hydrogenated and reduced, cleaving into aromatic amines.

#### 4.8.4 Rhodamine 6G Reduction Reaction

A 0.4 M sodium borohydride (NaBH_4_) solution was prepared. In a cuvette, 2745 μL of DI water is mixed with 5 μL of Rhodamine 6G (R6G) dye and 150 μL of the 0.4 M NaBH_4_, at which point the UV–vis extinction spectrum (200–600 nm) is recorded as T_0_. Finally, 100 μL of the nanocrystal suspension is added to the cuvette. The UV–vis spectrum is measured at specific intervals (3, 6, 9, 15, 30, and 60 min, followed by hourly measurements) until the extinction peaks show no further significant changes.

For the photocatalytic performance tests, the solution was irradiated with visible light at wavelengths corresponding to the specific extinction spectral modes of the Janus nanocrystals. This targeted irradiation is designed to enhance the localized surface plasmon resonance (LSPR) effect. The light source used for photocatalytic performance measurement was an LED point light source with its power at 9.9 W. The UV–vis spectrum is measured at specific intervals (3, 6, 9, 15, 30, and 60 min, followed by hourly measurements) until the extinction peaks show no further significant changes.

The reaction mechanism: rhodamine 6G and BH_4_^–^ ions in a catalyst and a photocatalyst (nanocrystals), and then the luminescent group is hydrogenated to convert into a colorless product.

## Supplementary Material



## Notes

HRTEM images, EDX analysis, and UV–vis spectroscopy of the nanocrystal composition during Janus nanocrystal formation, SEM image of Janus nanocrystals on PS/PDMS substrate, and UV–vis spectroscopy of nanocrystals as catalysts and photocatalysts for 4-NP reduction (PDF)
