# Supplementary material for: Janus-like Metallic Nanocrystals with Tunable Composition and Morphology Developed to Optimize their Catalytic and Photocatalytic Performance in the Reduction of 4‑NP and Organic Dyes
Source: Langmuir. 2026 Aug 12;42(33):24418–30. doi: 10.1021/acs.langmuir.6c03367 (PMC13523739; doi:10.1021/acs.langmuir.6c03367)
Supplement: Supplementary file 1 [file la6c03367_si_001.pdf]

Supporting information

Janus-like Metallic Nanocrystals with Tunable Composition and Morphology Developed to Optimize their Catalytic and Photocatalytic Performance in the Reduction of 4-NP and Organic Dyes

Chin-Chieh Yeh, Yu-Hsiang Chang, and Su-Wen Hsu*

Department of Chemical Engineering, National Cheng Kung University, Taiwan

No. 1 University Road, East Dist., Tainan City 70101, Taiwan (R.O.C)

*E-mail: swhsu@gs.ncku.edu.tw

Heterogeneous Nanocrystals characterization (SEM/HRTEM/UV-vis) Figure S1-S10
Heterogeneous Nanocrystals as 4-NP reduction catalysts Figure S11-S30
Heterogeneous Nanocrystals as 4-NP reduction photocatalysts Figure S31-S43
Heterogeneous Nanocrystals as organic dye reduction catalysts Figure S44

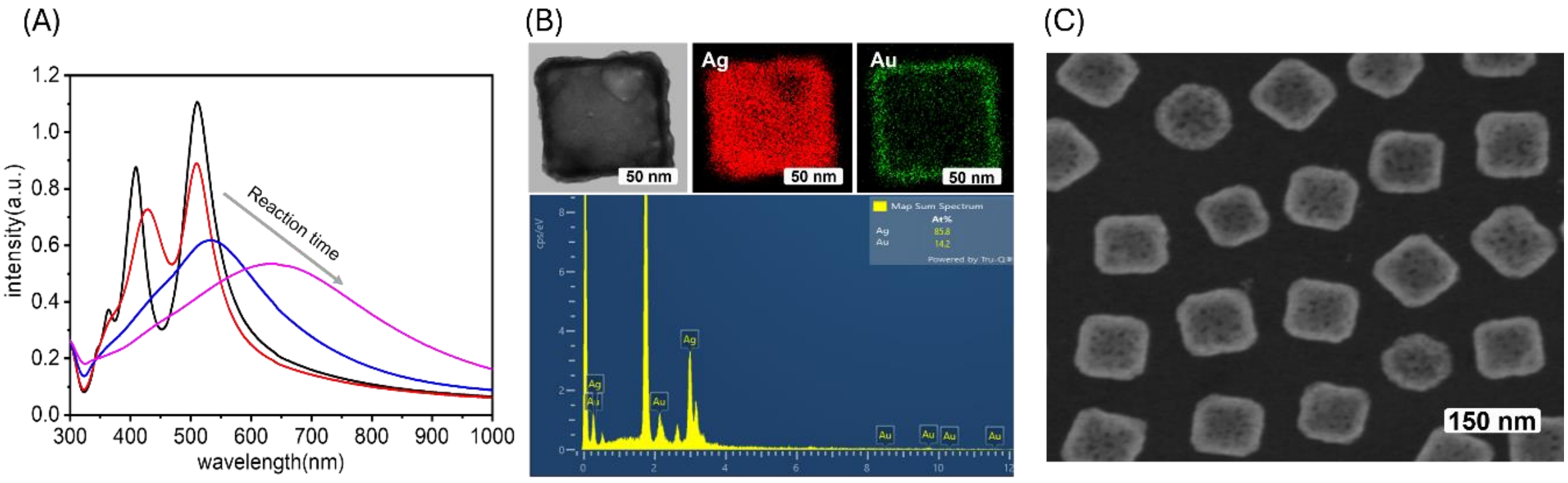

Figure S1 Au-Ag cage nanocrystals were prepared by seed-mediated synthesis. (A) Changes in the extinction spectrum during seed-mediated synthesis. (B) EDX spectrum showing the compositional distribution and atomic percentage of each component in the nanocrystals. (C) SEM image of the final product after seed-mediated synthesis.

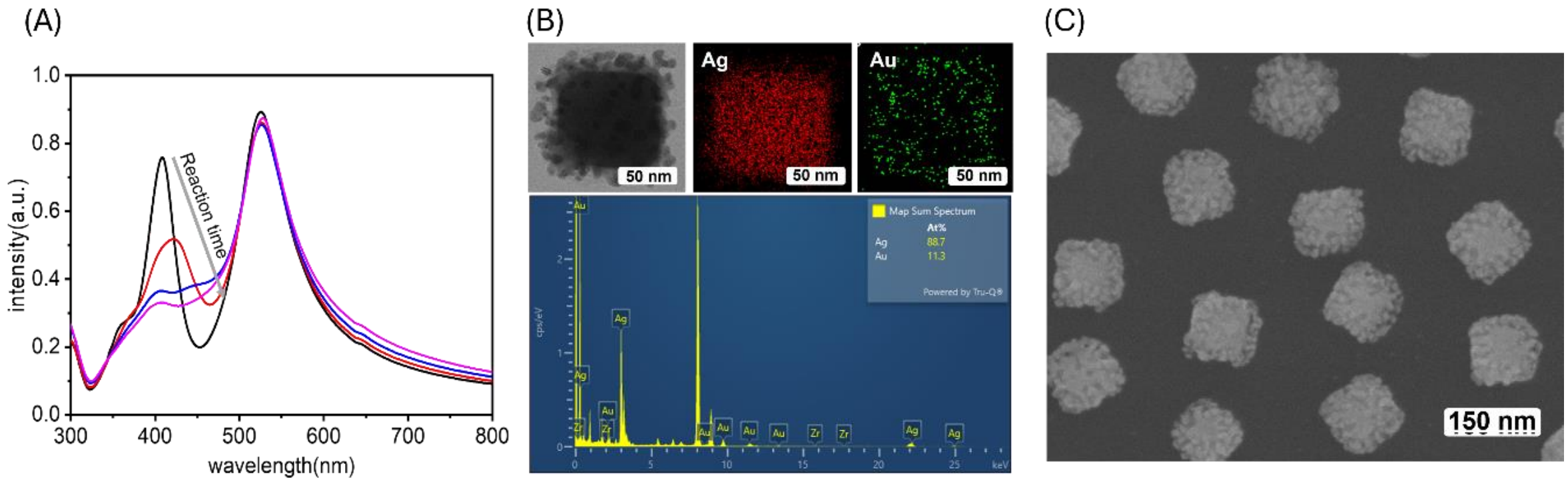

Figure S2 Au-Ag flower nanocrystals were prepared by seed-mediated synthesis. (A) Changes in the extinction spectrum during seed-mediated synthesis. (B) EDX spectrum showing the compositional distribution and atomic percentage of each component in the nanocrystals. (C) SEM image of the final product after seed-mediated synthesis.

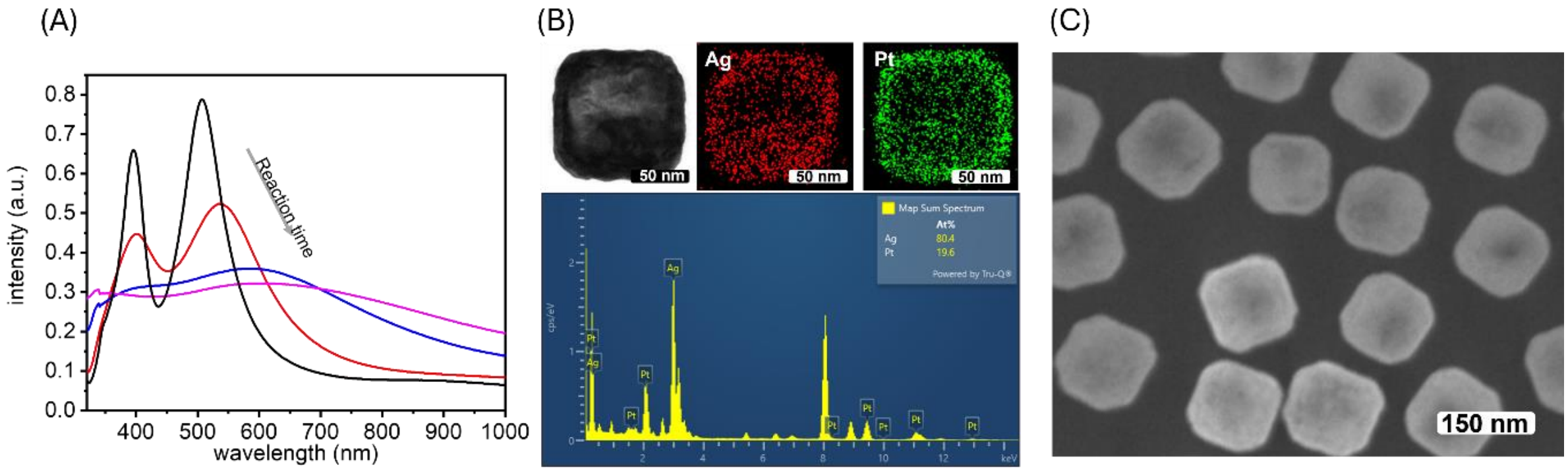

Figure S3 Pt-Ag cage nanocrystals were prepared by seed-mediated synthesis. (A) Changes in the extinction spectrum during seed-mediated synthesis. (B) EDX spectrum showing the compositional distribution and atomic percentage of each component in the nanocrystals. (C) SEM image of the final product after seed-mediated synthesis.

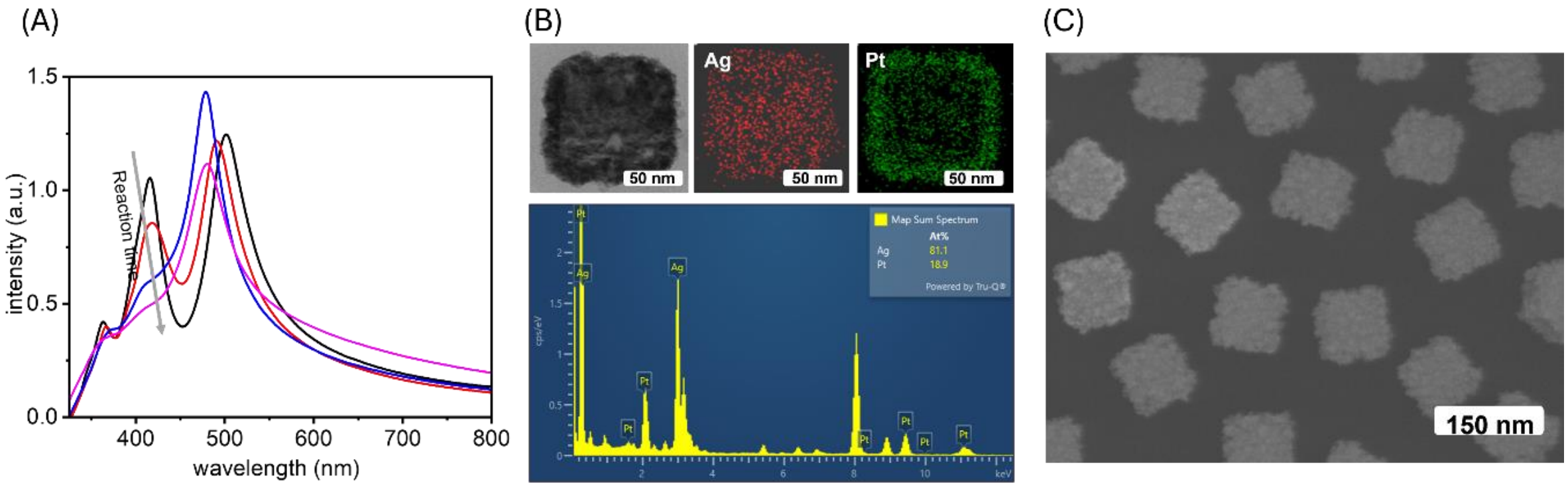

Figure S4 Pt-Ag flower nanocrystals were prepared by seed-mediated synthesis. (A) Changes in the extinction spectrum during seed-mediated synthesis. (B) EDX spectrum showing the compositional distribution and atomic percentage of each component in the nanocrystals. (C) SEM image of the final product after seed-mediated synthesis.

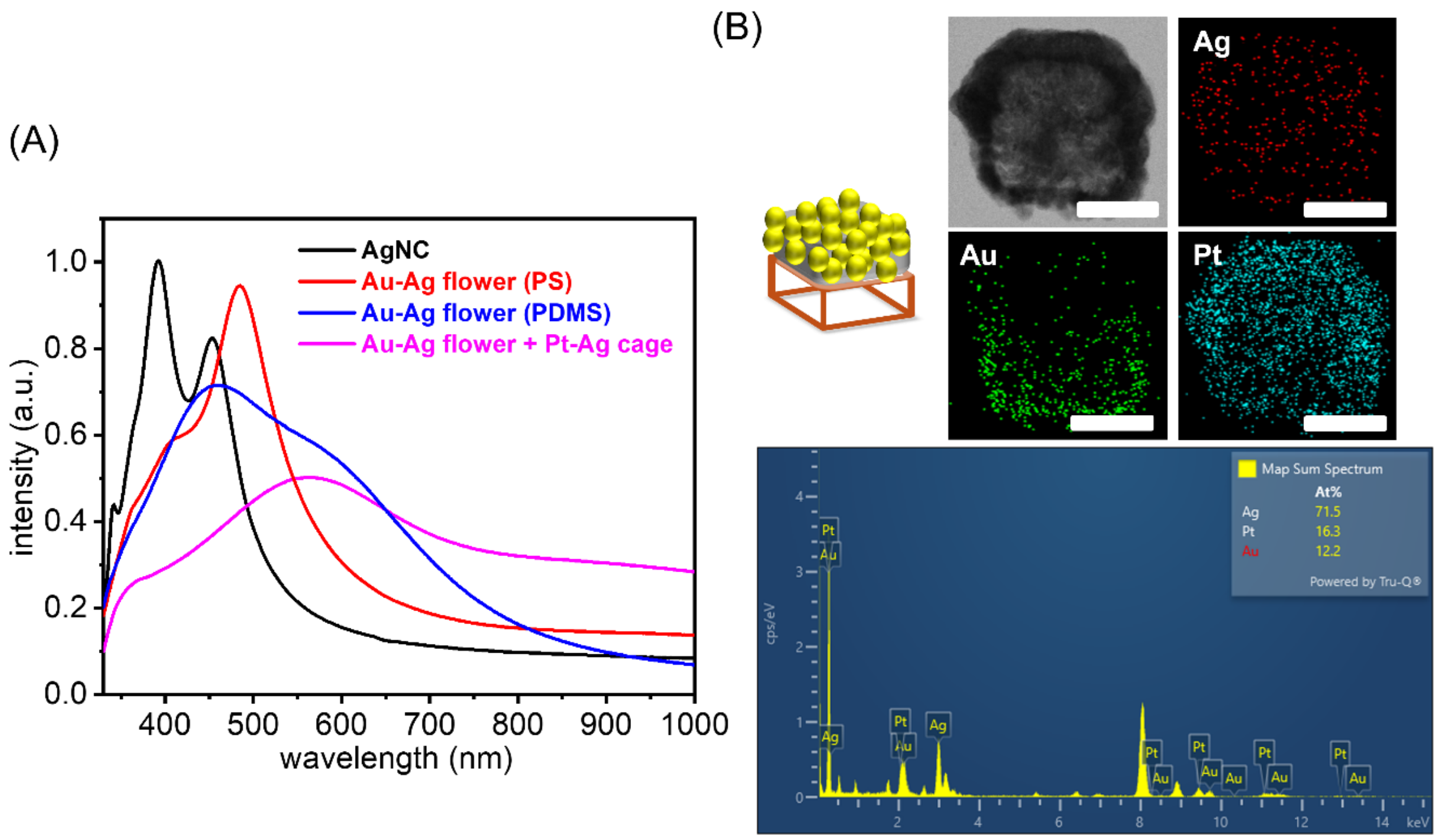

Figure S5 Janus-like Au-Ag flower + Pt-Ag cage nanocrystals were prepared by "two seed-mediated synthesis method". (A) Changes in the extinction spectrum during seed-mediated synthesis. Black curve: Ag nanocubes (AgNC) on PS matrix; Red curve: Au-Ag flower on PS matrix; Blue curve: Au-Ag flower on PDMS matrix; Purple curve: Au-Ag flower + Pt-Ag cage on PDMS matrix. (B) EDX spectrum showing the compositional distribution and atomic percentage of each component in the nanocrystals. Scale bar: 50 nm.

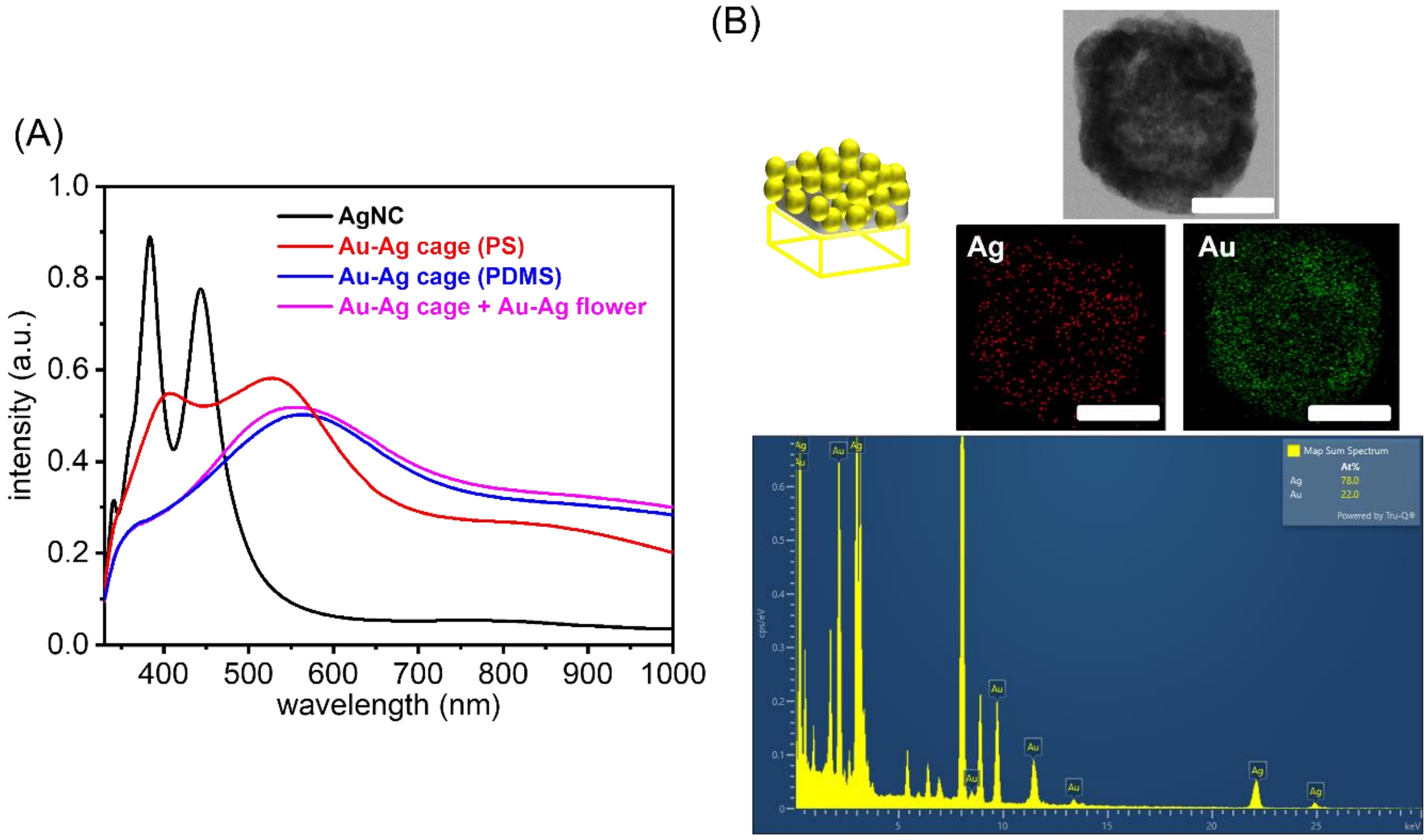

Figure S6 Janus-like Au-Ag cage + Au-Ag flower nanocrystals were prepared by “two seed-mediated synthesis method”. (A) Changes in the extinction spectrum during seed-mediated synthesis. Black curve: Ag nanocubes (AgNC) on PS matrix; Red curve: Au-Ag cage on PS matrix; Blue curve: Au-Ag cage on PDMS matrix; Purple curve: Au-Ag flower + Au-Ag cage on PDMS matrix. (B) EDX spectrum showing the compositional distribution and atomic percentage of each component in the nanocrystals. Scale bar: 50 nm.

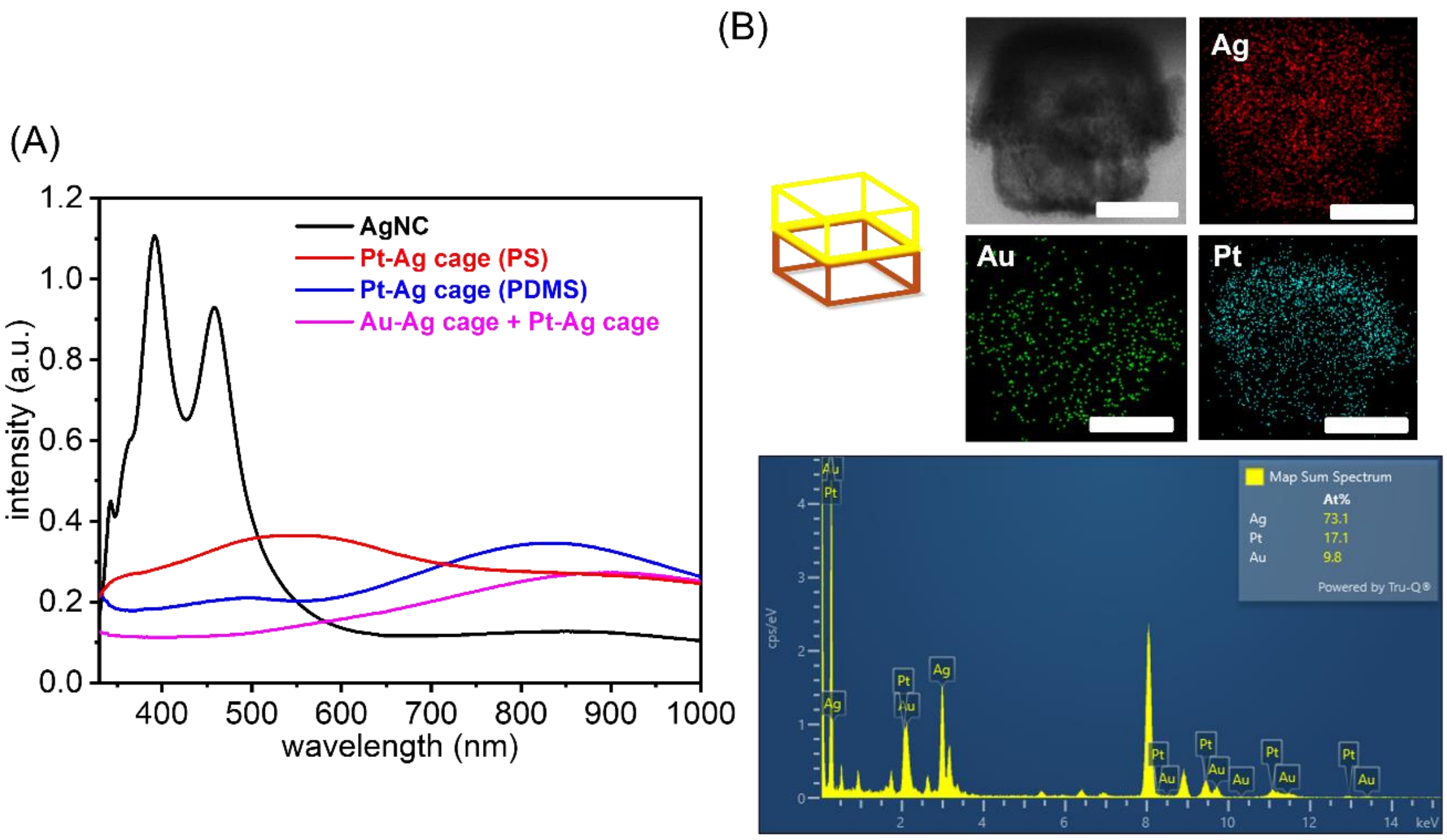

Figure S7 Janus-like Pt-Ag cage + Au-Ag cage nanocrystals were prepared by "two seed-mediated synthesis method". (A) Changes in the extinction spectrum during seed-mediated synthesis. Black curve: Ag nanocubes (AgNC) on PS matrix; Red curve: Pt-Ag cage on PS matrix; Blue curve: Pt-Ag cage on PDMS matrix; Purple curve: Pt-Ag cage + Au-Ag cage on PDMS matrix. (B) EDX spectrum showing the compositional distribution and atomic percentage of each component in the nanocrystals. Scale bar: 50 nm.

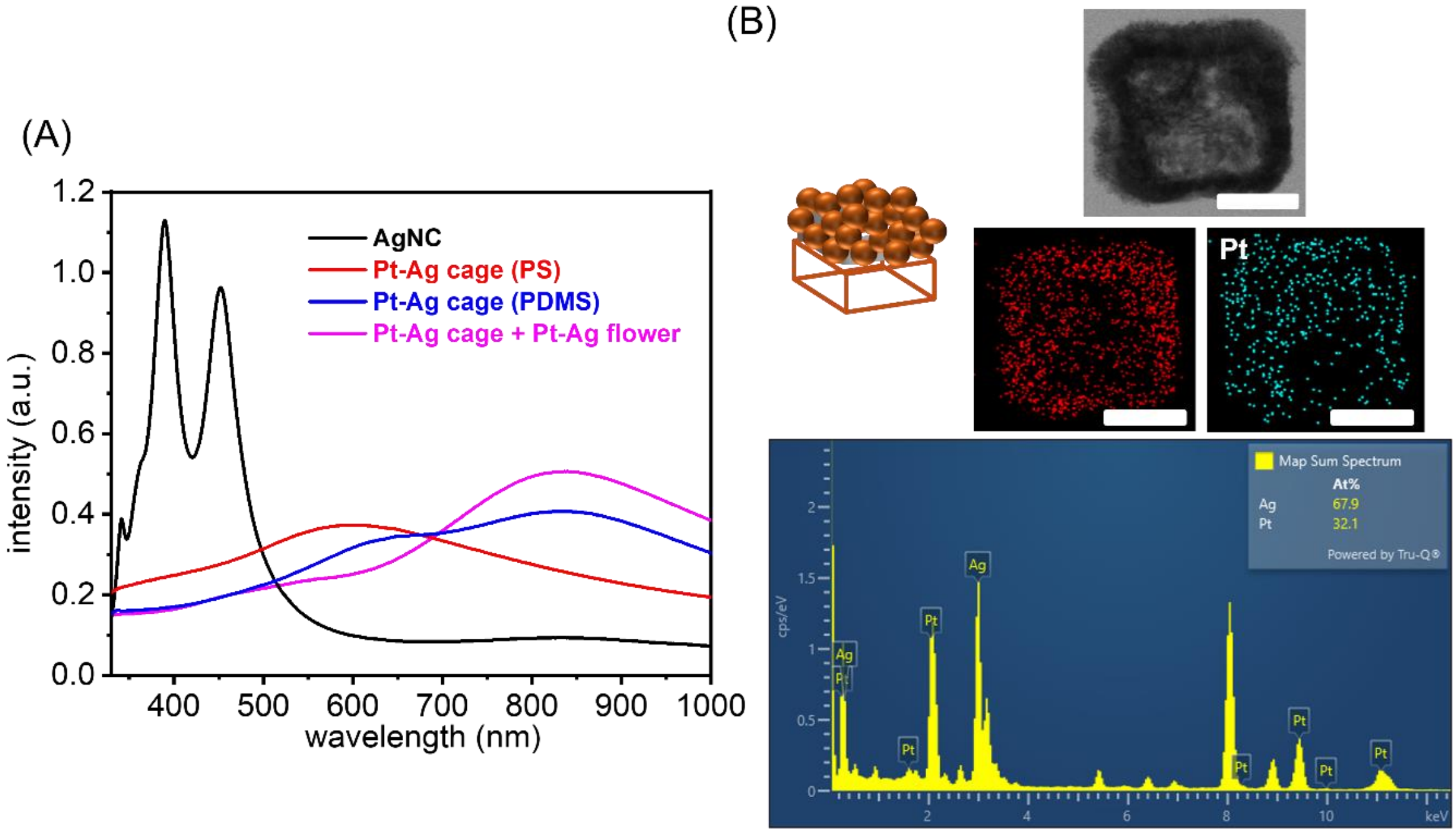

Figure S8 Janus-like Pt-Ag cage + Pt-Ag flower nanocrystals were prepared by "two seed-mediated synthesis method". (A) Changes in the extinction spectrum during seed-mediated synthesis. Black curve: Ag nanocubes (AgNC) on PS matrix; Red curve: Pt-Ag cage on PS matrix; Blue curve: Pt-Ag cage on PDMS matrix; Purple curve: Pt-Ag cage + Pt-Ag flower on PDMS matrix. (B) EDX spectrum showing the compositional distribution and atomic percentage of each component in the nanocrystals. Scale bar: 50 nm.

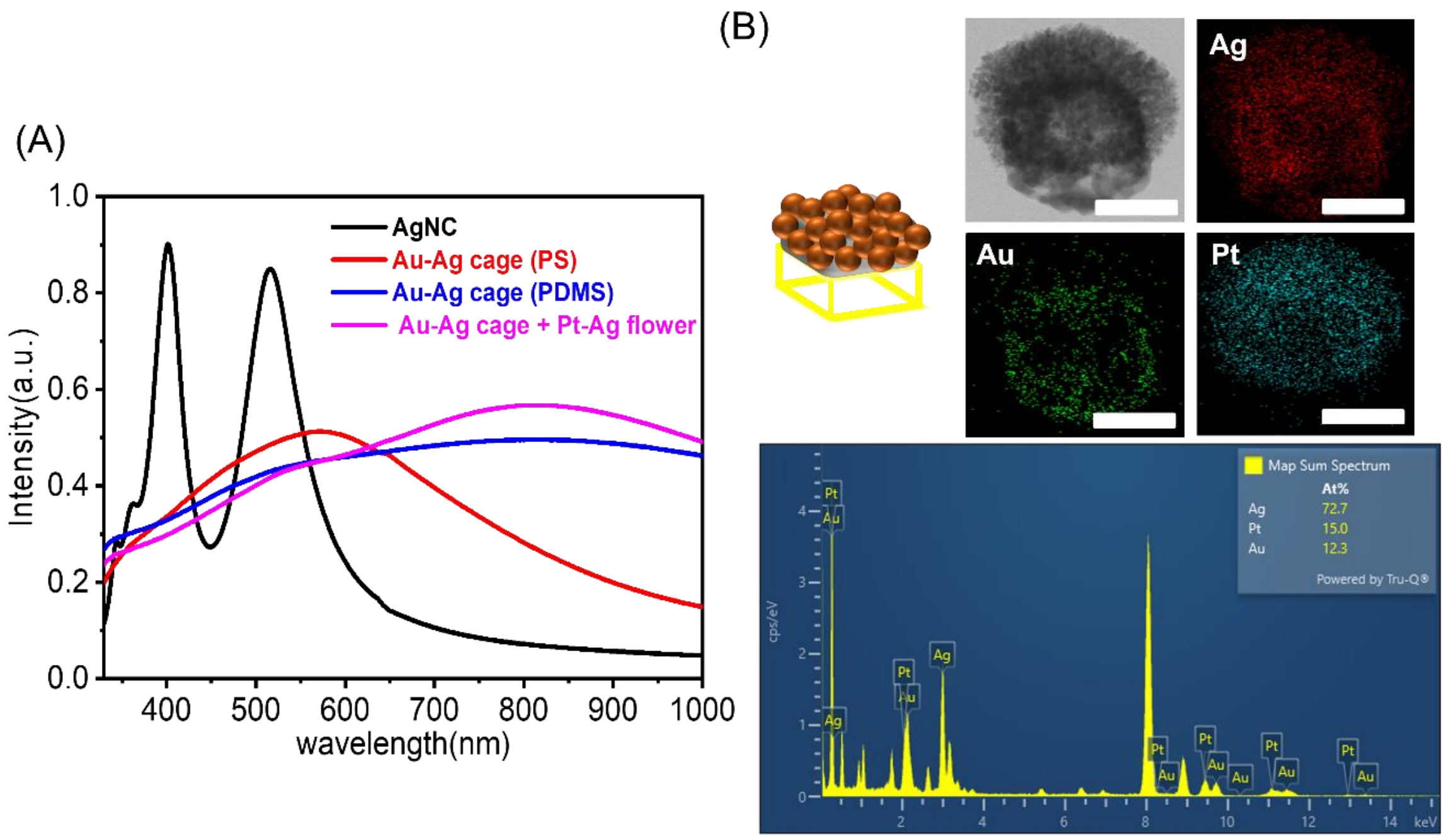

Figure S9 Janus-like Au-Ag cage + Pt-Ag flower nanocrystals were prepared by “two seed-mediated synthesis method”. (A) Changes in the extinction spectrum during seed-mediated synthesis. Black curve: Ag nanocubes (AgNC) on PS matrix; Red curve: Au-Ag cage on PS matrix; Blue curve: Au-Ag cage on PDMS matrix; Purple curve: Au-Ag cage + Pt-Ag flower on PDMS matrix. (B) EDX spectrum showing the compositional distribution and atomic percentage of each component in the nanocrystals. Scale bar: 50 nm.

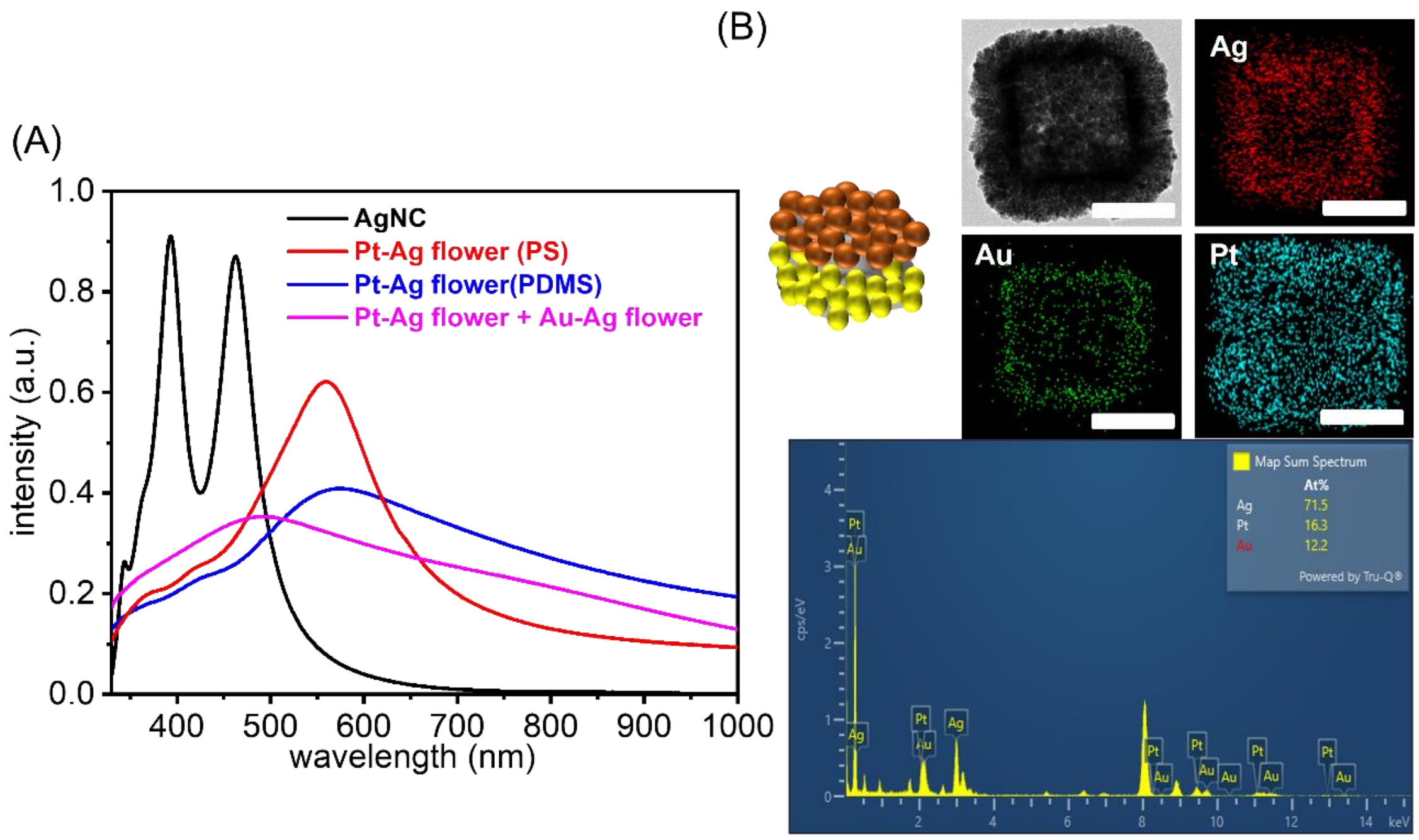

Figure S10 Janus-like Pt-Ag flower + Au-Ag flower nanocrystals were prepared by "two seed-mediated synthesis method". (A) Changes in the extinction spectrum during seed-mediated synthesis. Black curve: Ag nanocubes (AgNC) on PS matrix; Red curve: Pt-Ag flower on PS matrix; Blue curve: Pt-Ag flower on PDMS matrix; Purple curve: Pt-Ag flower + Au-Ag flower on PDMS matrix. (B) EDX spectrum showing the compositional distribution and atomic percentage of each component in the nanocrystals. Scale bar: 50 nm.

Table S1 Lists of the compositional composition of bimetallic nanocrystals prepared by seed-mediated synthesis and the compositional changes of Janus-like metallic nanocrystal through "two-seed-mediated synthesis".

	Bi-component nanocrystal			
	I	II	III	IV
	Au-Ag cage	Au-Ag flower	Pt-Ag cage	Pt-Ag flower
Ag at %	85.8	88.7	80.4	81.1
Au at%	14.2	11.3	---	---
Pt at %	---	---	19.6	18.9

	Janus nanocrystal																	
	Au-Ag flower + Pt-Ag cage			Au-Ag cage+ Au-Ag flower			Pt-Ag cage + Au-Ag cage			Pt-Ag cage + Pt-Ag flower			Au-Ag cage + Pt-Ag flower			Pt-Ag flower + Au-Ag flower		
	1st rxn (I)	2nd rxn		1st rxn (II)	2nd rxn		1st rxn (III)	2nd rxn		1st rxn (I)	2nd rxn		1st rxn (I)	2nd rxn		1st rxn (IV)	2nd rxn	
		Theor.	Expt.		Theor.	Expt.		Theor.	Expt.		Theor.	Expt.		Theor.	Expt.		Theor.	Expt.
Ag at %	88.7	70.7	71.5	85.8	78.0	78.0	80.4	73.1	73.1	80.4	67.9	67.9	85.8	72.7	72.7	81.1	71.2	71.5
Au at%	11.3	13.1	12.2	14.2	22.0	22.0	---	9.8	9.8	---	---	---	14.2	12.3	12.3	---	12.1	12.2
Pt at %	---	16.1	16.3	---	---	---	19.6	17.1	17.1	19.6	32.1	32.1	---	15.0	15.0	18.9	16.6	16.3
	2nd rxn: Pt : Ag =18.5 : 81.5			2nd rxn: Au : Ag =10.4 : 89.6			2nd rxn Au : Ag =11.8 : 88.2 (36% Au from galvanic replacement of Pt atom)			2nd rxn: Pt : Ag =18.5 : 81.5			2nd rxn: Pt : Ag =17.1 : 82.9			2nd rxn: Au : Ag = 14.6: 85.4		

PS:

(1) The flower-like Au-Ag and Pt-Ag nanocrystals generated in the first seed-mediated synthesis and the Janus-like nanocrystals generated in the subsequent second seed-mediated synthesis exhibit strong interactions between the nanocrystals and PDMS. During the PDMS removal process, some of the petals of the flower-like nanocrystals fall off, resulting in changes in composition.

(2) During the second seed-mediated synthesis, when the Au-Ag cage structure is formed, not only Ag atoms but also Pt atoms can be reduced to ions through a galvanic replacement reaction with Au^{3+} ions.

Table S2 Catalyst concentration (mg/ml) was calculated through heat treatment.

	Bi-component nanocrystal				Janus nanocrystal					
	Au-Ag cage	Au-Ag flower	Pt-Ag cage	Pt-Ag flower	Au-Ag flower+ Pt-Ag cage	Au-Ag flower+ Au-Ag cage	Au-Ag cage+ Pt-Ag cage	Pt-Ag flower+ Pt-Ag cage	Pt-Ag flower+ Au-Ag cage	Pt-Ag flower+ Au-Ag flower
weight of nanocrystal in colloidal solution (mg)	0.047	0.090	0.086	0.082	0.118	0.106	0.092	0.121	0.086	0.066
volume of colloidal solution (mL)	0.250	0.425	0.475	0.425	0.737	0.625	0.500	0.737	0.500	0.352
Concentration of colloidal solution (mg/mL)	0.190	0.210	0.180	0.191	0.160	0.170	0.184	0.164	0.172	0.188

PS: (1) the weight of nanocrystal in colloidal solution was obtained through heat treatment.
(2) concentration of catalysis for dye reduction (mg/ mL)
= concentration of colloidal solution × injection volume of colloidal solution / total volume of dye solution.

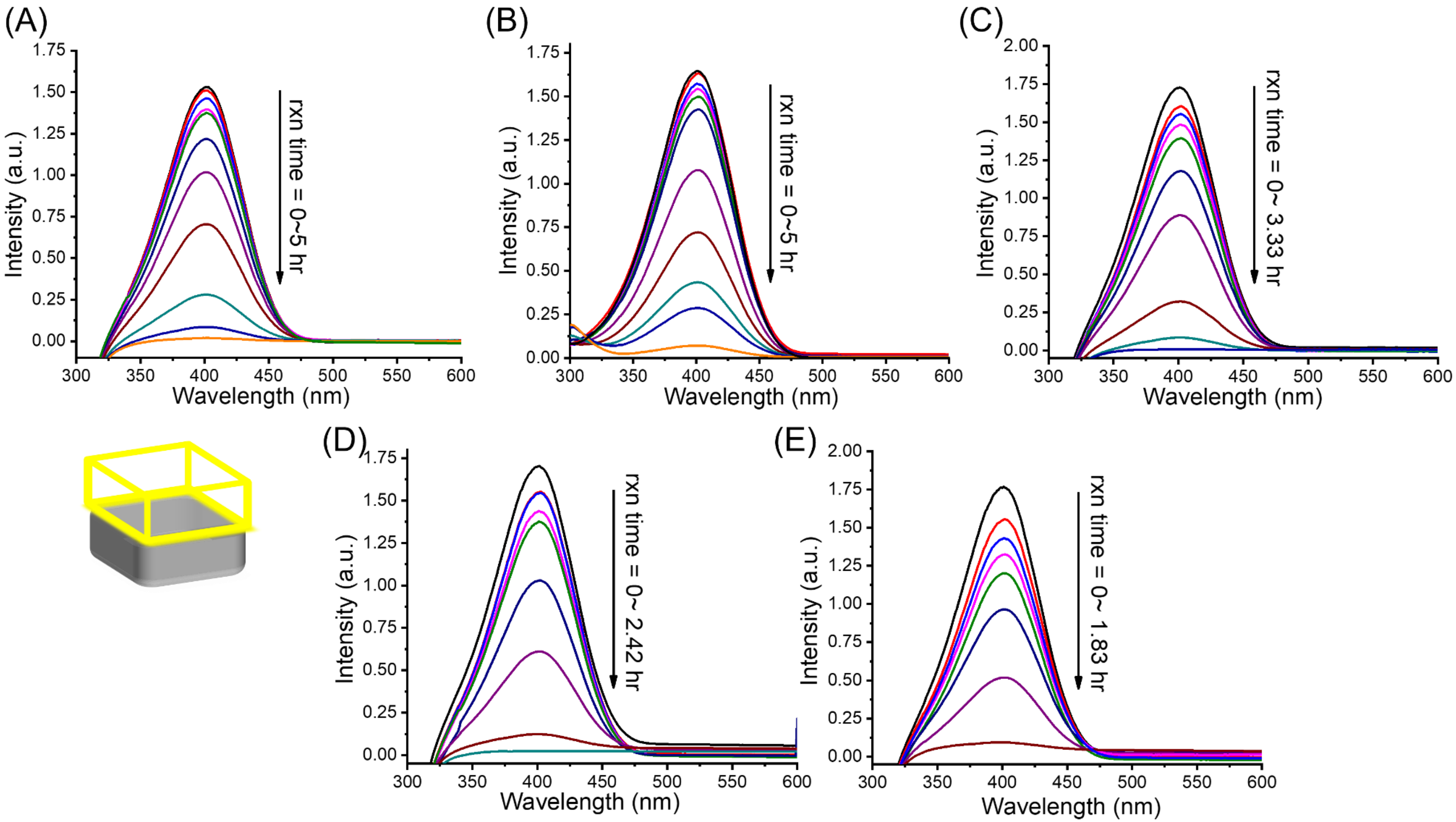

Figure S11 The reduction of 4-NP was carried out using Au-Ag cage nanocrystals as catalysts. The 4-NP concentration varied with reaction time under different catalyst concentrations. The catalyst concentration increased sequentially from (A) to (E).

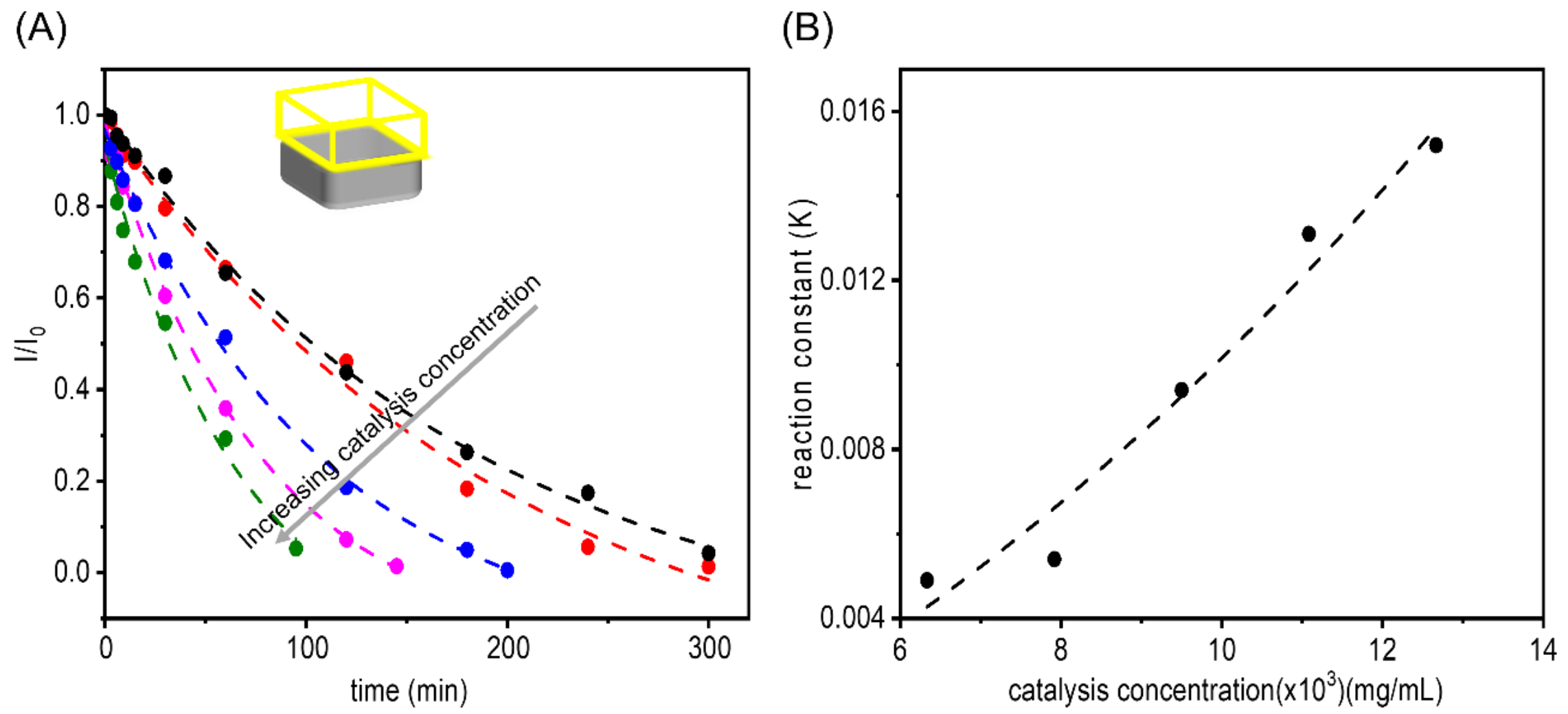

Figure S12 The reduction of 4-NP was carried out using Au-Ag cage nanocrystal as catalysts. (A) Concentration change (I/I_0, I: concentration of 4-NP during the reaction, I_0: initial concentration of 4-NP) over time. Solid dots represent experimental data. Dashed lines represent the fitted curve of the first-order reaction mechanism ($\frac{I}{I_0} = y_0 + A \times e^{-(t/t_0)}$ and t_0= 1/reaction constant (K)). (B) The reaction constant (K) varies with catalyst concentration.

Table S3 The reaction constant (K) of reduction of 4-NP was carried out using Au-Ag cage nanocrystal as catalysts which resulted from $\frac{I}{I_0} = y_0 + A \times e^{-(t/t_0)}$.

Concentration of catalysts($\times 10^3$ mg/mL)	Fitting function : $I/I_0 = y_0 + A*exp(-t/t_0)$, $K=1/t_0$				
	y_0	A	t_0	$K=1/t_0$	R-square
6.333	-0.311	1.304	201.959	0.0050	0.994
7.917	-0.179	1.183	186.284	0.0054	0.997
9.500	-0.174	1.140	108.718	0.0092	0.997
11.083	-0.160	1.145	76.001	0.0132	0.997
12.667	-0.193	1.128	65.672	0.0152	0.981

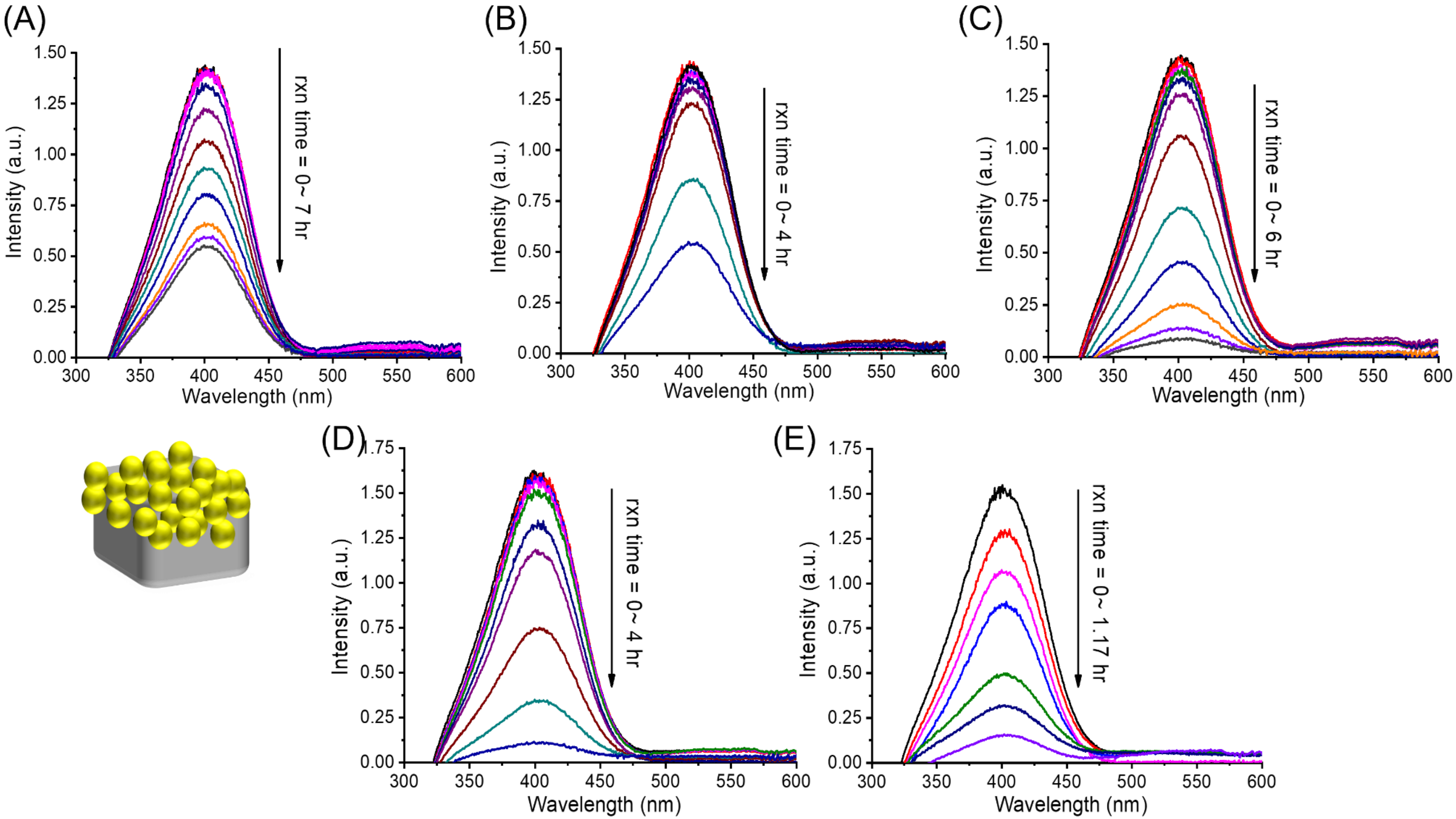

Figure S13 The reduction of 4-NP was carried out using Au-Ag flower nanocrystals as catalysts. The 4-NP concentration varied with reaction time under different catalyst concentrations. The catalyst concentration increased sequentially from (A) to (E).

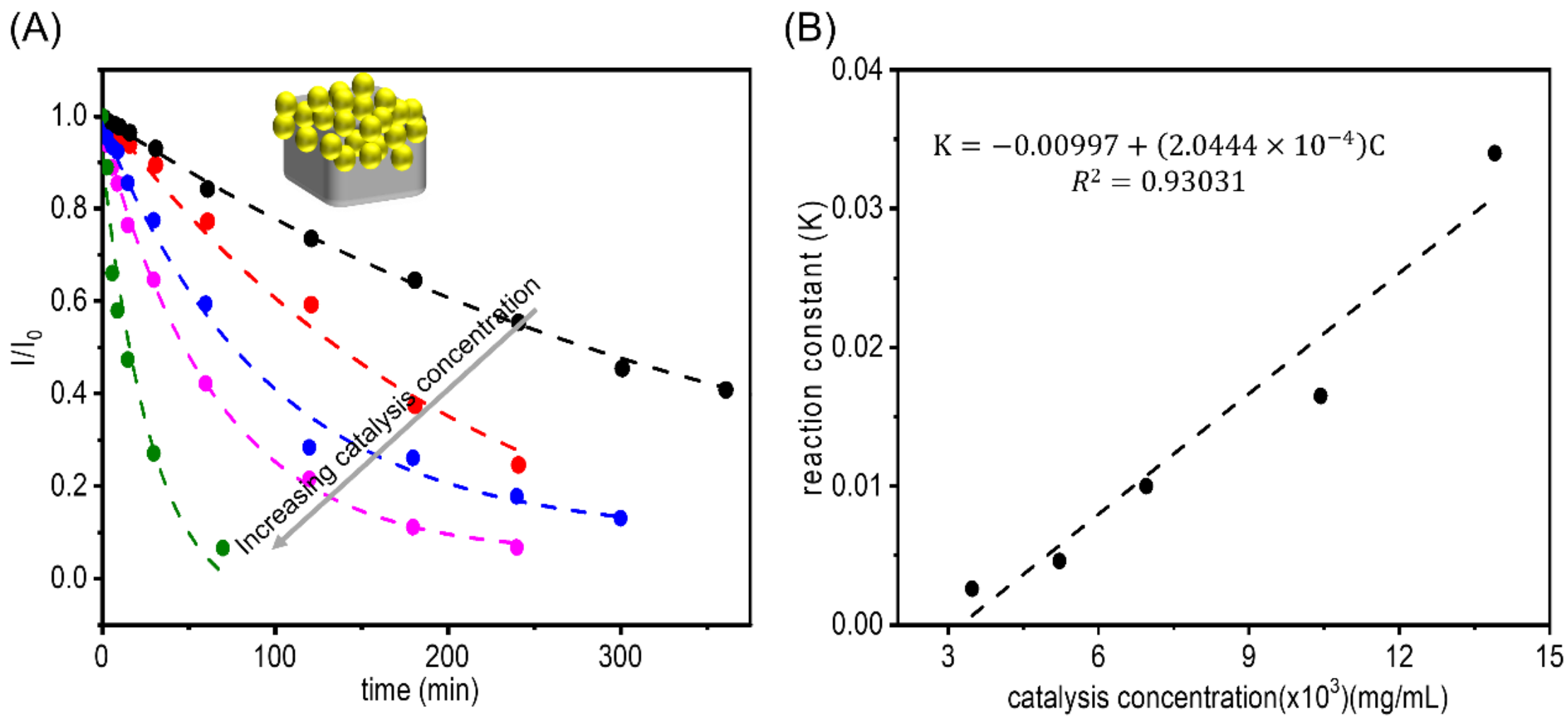

Figure S14 The reduction of 4-NP was carried out using Au-Ag flower nanocrystal as catalysts. (A) Concentration change (I/I_0, I: concentration of 4-NP during the reaction, I_0: initial concentration of 4-NP) over time. Solid dots represent experimental data. Dashed lines represent the fitted curve of the first-order reaction mechanism ($\frac{I}{I_0} = y_0 + A \times e^{-(t/t_0)}$ and t_0 = 1/reaction constant (K)). (B) The reaction constant (K) varies with catalyst concentration.

Table S4 The reaction constant (K) of reduction of 4-NP was carried out using Au-Ag flower nanocrystal as catalysts which resulted from $\frac{I}{I_0} = y_0 + A \times e^{-(t/t_0)}$.

Concentration of catalysts($\times 10^3$ mg/mL)	Fitting function : $I/I_0 = y_0 + A*\exp(-t/t_0)$, $K=1/t_0$				
	y_0	A	t_0	$K=1/t_0$	R-square
3.478	0.035	0.964	384.628	0.0026	0.998
5.217	-0.086	1.097	217.324	0.0046	0.989
6.956	0.188	0.846	100.039	0.0099	0.953
10.434	0.069	0.916	60.885	0.0164	0.998
13.912	-0.082	1.002	29.411	0.0340	0.949

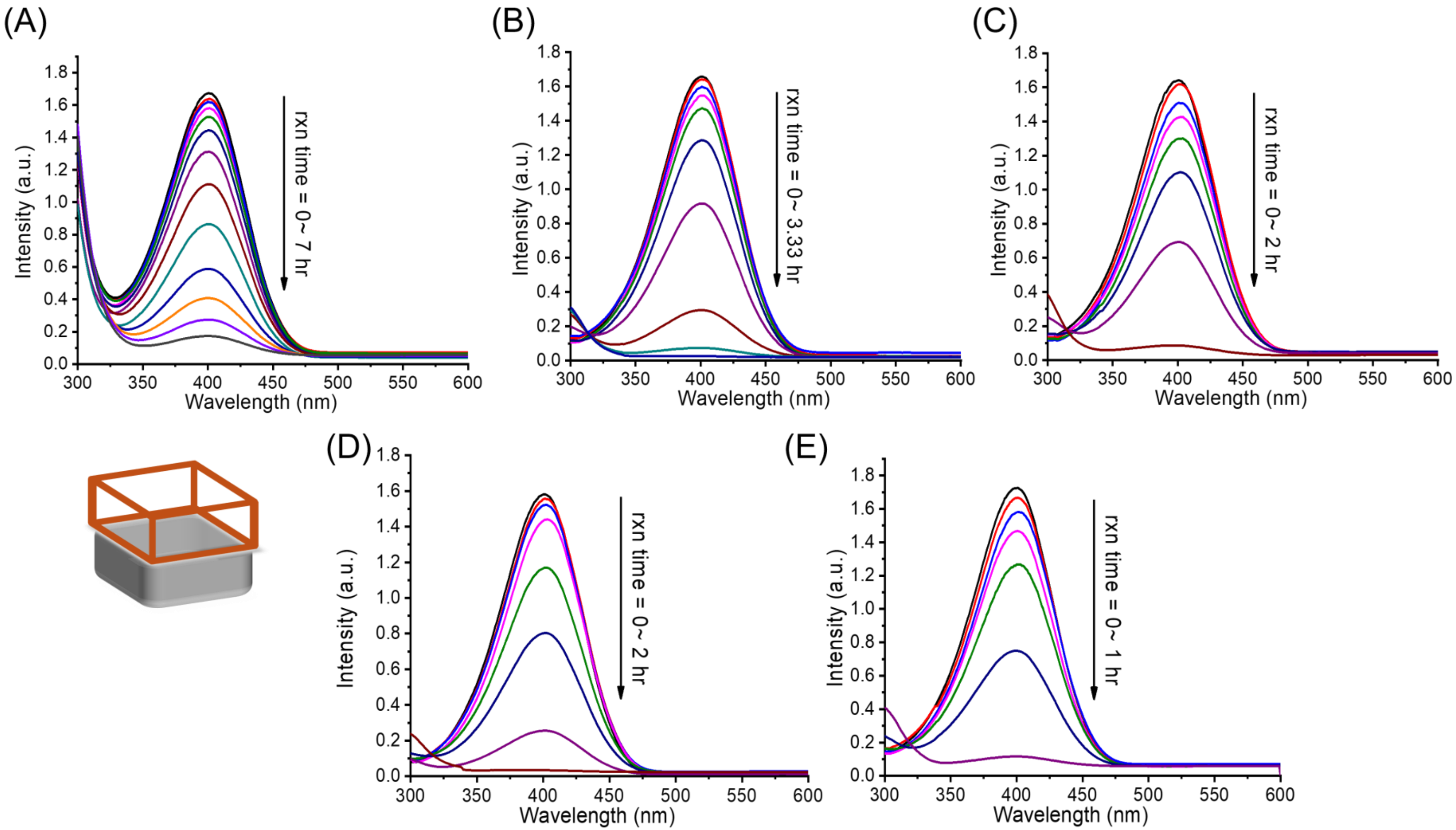

Figure S15 The reduction of 4-NP was carried out using Pt-Ag cage nanocrystals as catalysts. The 4-NP concentration varied with reaction time under different catalyst concentrations. The catalyst concentration increased sequentially from (A) to (E).

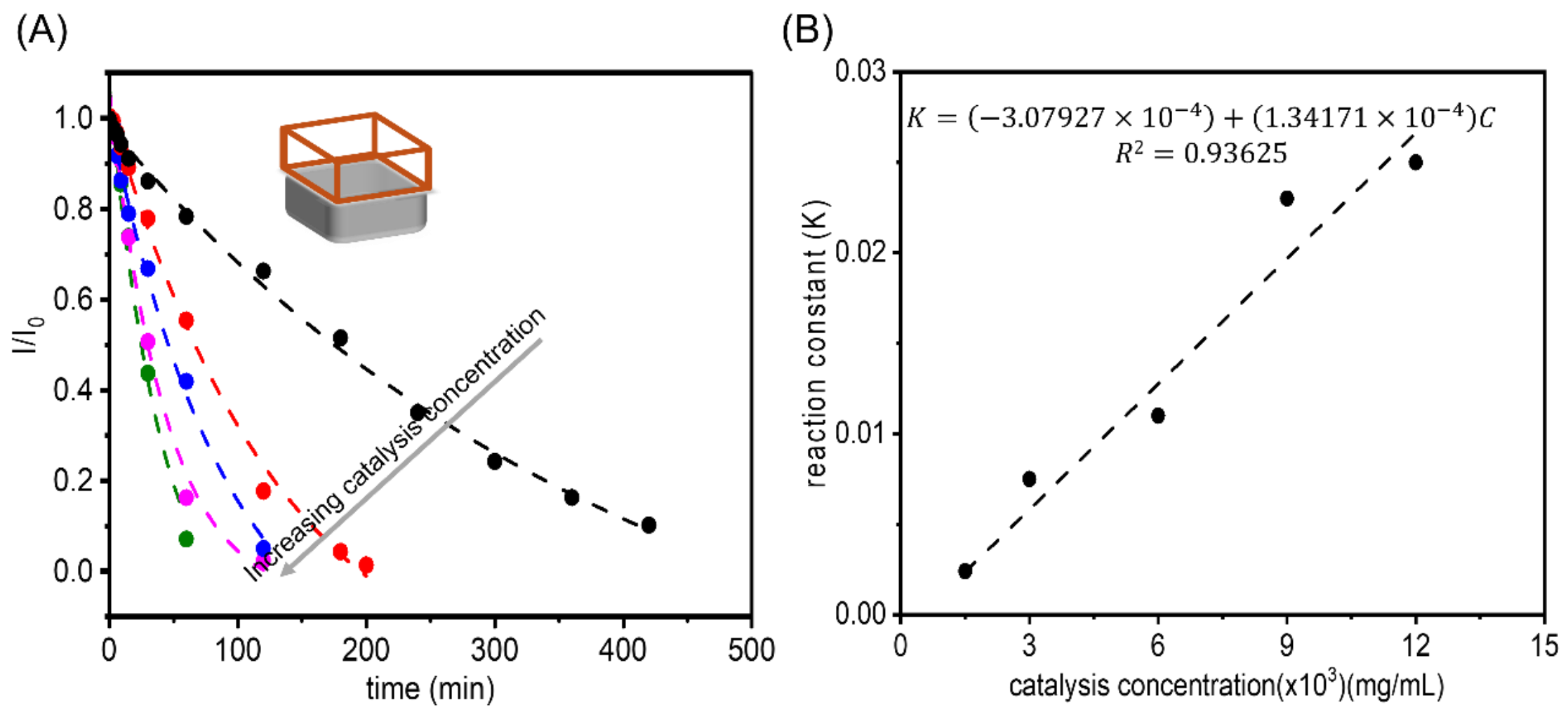

Figure S16 The reduction of 4-NP was carried out using Pt-Ag cage nanocrystal as catalysts. (A) Concentration change (I/I_0, I: concentration of 4-NP during the reaction, I_0: initial concentration of 4-NP) over time. Solid dots represent experimental data. Dashed lines represent the fitted curve of the first-order reaction mechanism ($\frac{I}{I_0} = y_0 + A \times e^{-(t/t_0)}$ and t_0 = 1/reaction constant (K)). (B) The reaction constant (K) varies with catalyst concentration.

Table S5 The reaction constant (K) of reduction of 4-NP was carried out using Pt-Ag cage nanocrystal as catalysts which resulted from $\frac{I}{I_0} = y_0 + A \times e^{-(t/t_0)}$.

Concentration of catalysts($\times 10^3$ mg/mL)	Fitting function : $I/I_0 = y_0 + A*\exp(-t/t_0)$, $K=1/t_0$				
	y_0	A	t_0	$K=1/t_0$	R-square
1.497	-0.419	1.401	416.67	0.0024	0.997
2.994	-0.304	1.324	133.33	0.0075	0.996
5.988	-0.265	1.272	90.9	0.0110	0.995
8.982	-0.072	1.139	43.478	0.0230	0.986
11.976	-0.162	1.230	39.887	0.0251	0.981

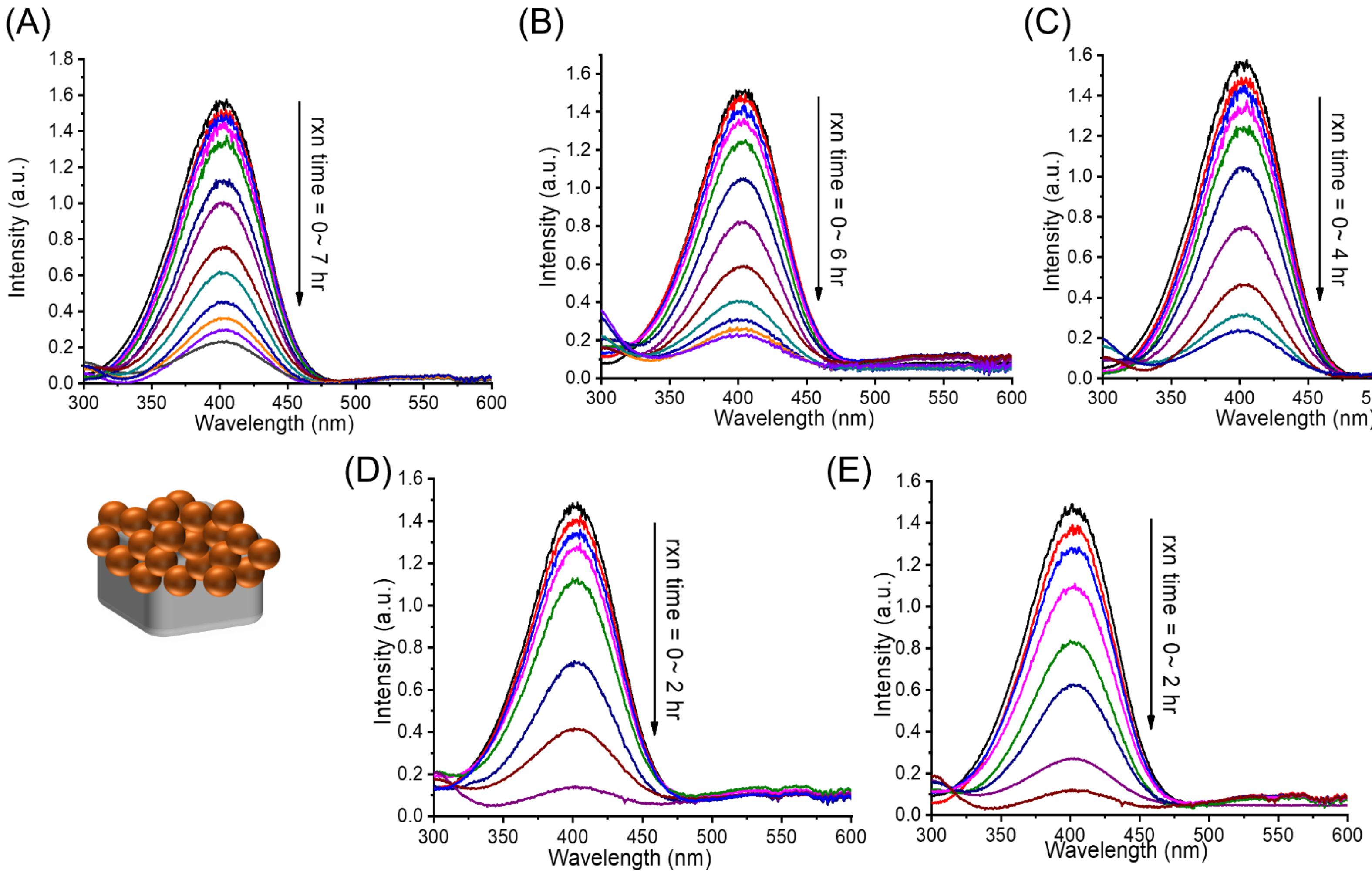

Figure S17 The reduction of 4-NP was carried out using Pt-Ag flower nanocrystals as catalysts. The 4-NP concentration varied with reaction time under different catalyst concentrations. The catalyst concentration increased sequentially from (A) to (E).

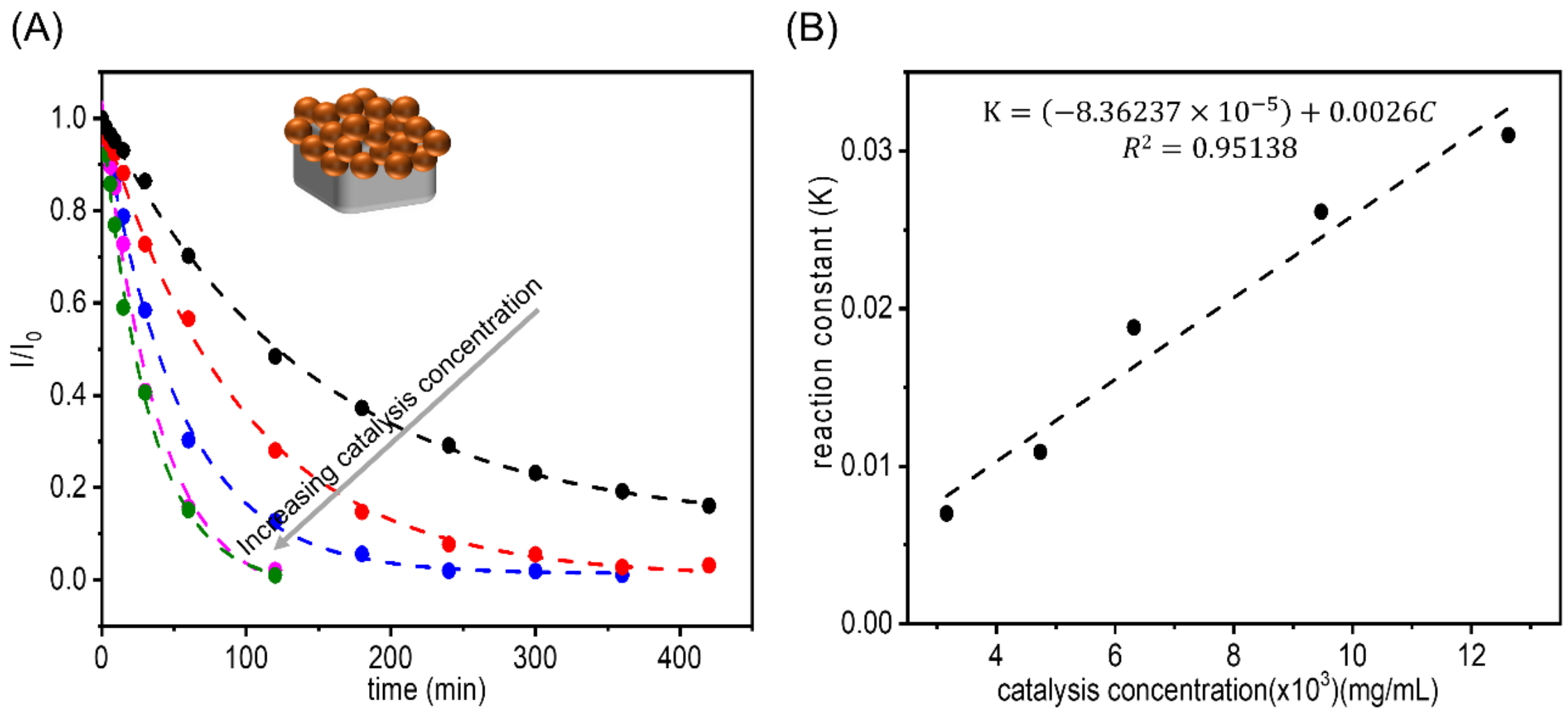

Figure S18 The reduction of 4-NP was carried out using Pt-Ag flower nanocrystal as catalysts. (A) Concentration change (I/I_0, I: concentration of 4-NP during the reaction, I_0: initial concentration of 4-NP) over time. Solid dots represent experimental data. Dashed lines represent the fitted curve of the first-order reaction mechanism ($\frac{I}{I_0} = y_0 + A \times e^{-(t/t_0)}$and t_0 = 1/reaction constant (K)). (B) The reaction constant (K) varies with catalyst concentration.

Table S6 The reaction constant (K) of reduction of 4-NP was carried out using Pt-Ag flower nanocrystal as catalysts which resulted from $\frac{I}{I_0} = y_0 + A \times e^{-(t/t_0)}$.

Concentration of catalysts($\times 10^3$ mg/mL)	Fitting function : $I/I_0= y_0+A*\exp(-t/t_0)$, $K=1/t_0$				
	y_0	A	t_0	$K=1/t_0$	R-square
3.156	0.120	0.888	142.624	0.0070	0.998
4.737	0.135	0.832	91.734	0.0109	0.994
6.313	0.182	0.808	53.198	0.0188	0.994
9.470	-0.010	0.978	38.206	0.0261	0.976
12.627	0.117	0.935	32.253	0.0310	0.986

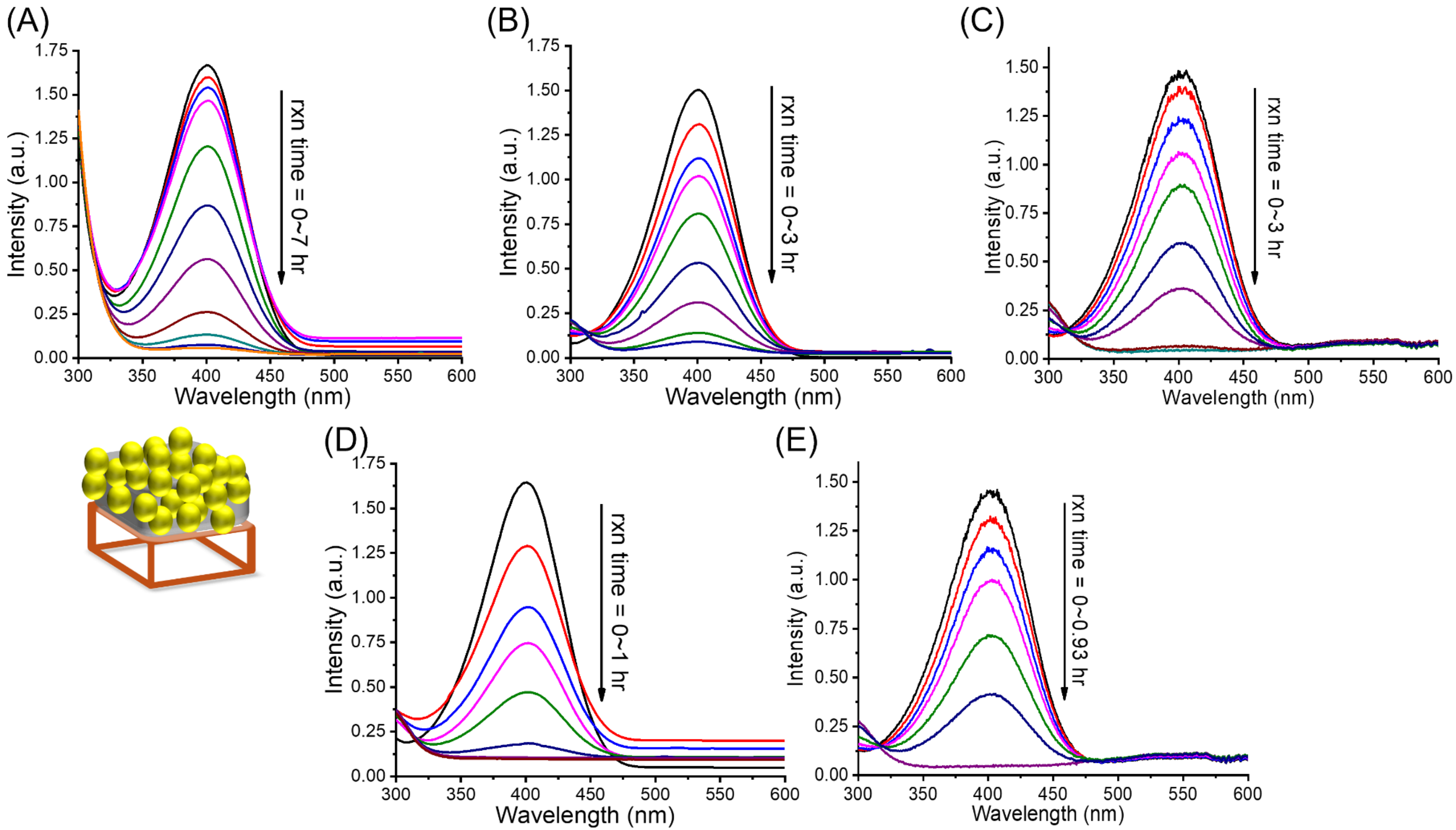

Figure S19 The reduction of 4-NP was carried out using Au-Ag flower + Pt-Ag cage nanocrystals as catalysts. The 4-NP concentration varied with reaction time under different catalyst concentrations. The catalyst concentration increased sequentially from (A) to (E).

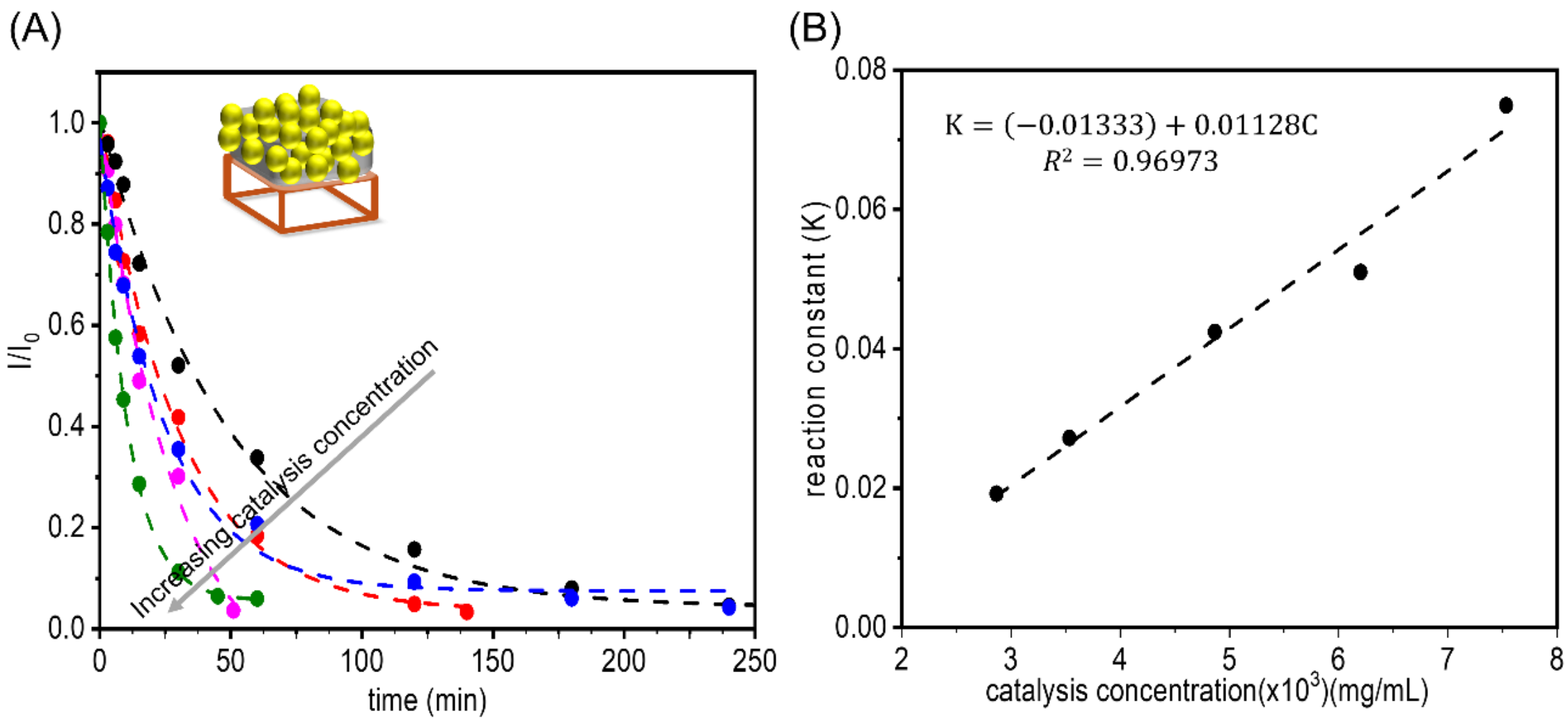

Figure S20 The reduction of 4-NP was carried out using Au-Ag flower + Pt-Ag cage nanocrystal as catalysts. (A) Concentration change (I/I_0, I: concentration of 4-NP during the reaction, I_0: initial concentration of 4-NP) over time. Solid dots represent experimental data. Dashed lines represent the fitted curve of the first-order reaction mechanism ($\frac{I}{I_0} = y_0 + A \times e^{-(t/t_0)}$ and t_0 = 1/reaction constant (K)). (B) The reaction constant (K) varies with catalyst concentration.

Table S7 The reaction constant (K) of reduction of 4-NP was carried out using Au-Ag flower + Pt-Ag cage nanocrystal as catalysts which resulted from $\frac{I}{I_0} = y_0 + A \times e^{-(t/t_0)}$.

Concentration of catalysts($\times 10^3$ mg/mL)	Fitting function : $I/I_0 = y_0 + A*\exp(-t/t_0)$, $K=1/t_0$				
	y_0	A	t_0	$K=1/t_0$	R-square
2.855	0.037	0.965	51.926	0.0193	0.995
3.522	0.480	0.786	36.676	0.0273	0.990
4.856	0.091	0.880	23.595	0.0424	0.992
6.188	0.019	1.022	19.766	0.0506	0.984
7.524	0.013	0.944	13.354	0.0749	0.989

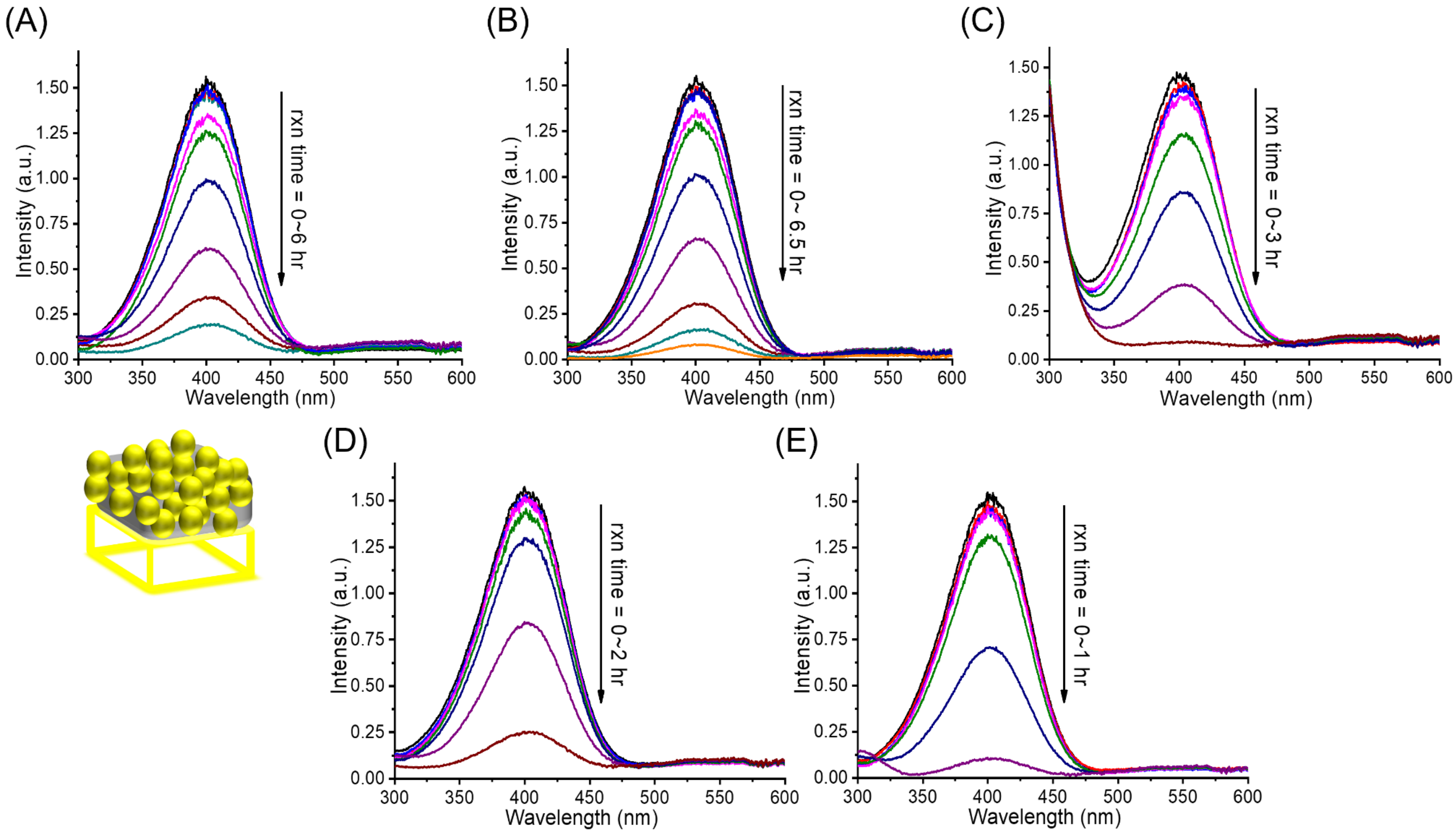

Figure S21 The reduction of 4-NP was carried out using Au-Ag flower + Au-Ag cage nanocrystals as catalysts. The 4-NP concentration varied with reaction time under different catalyst concentrations. The catalyst concentration increased sequentially from (A) to (E).

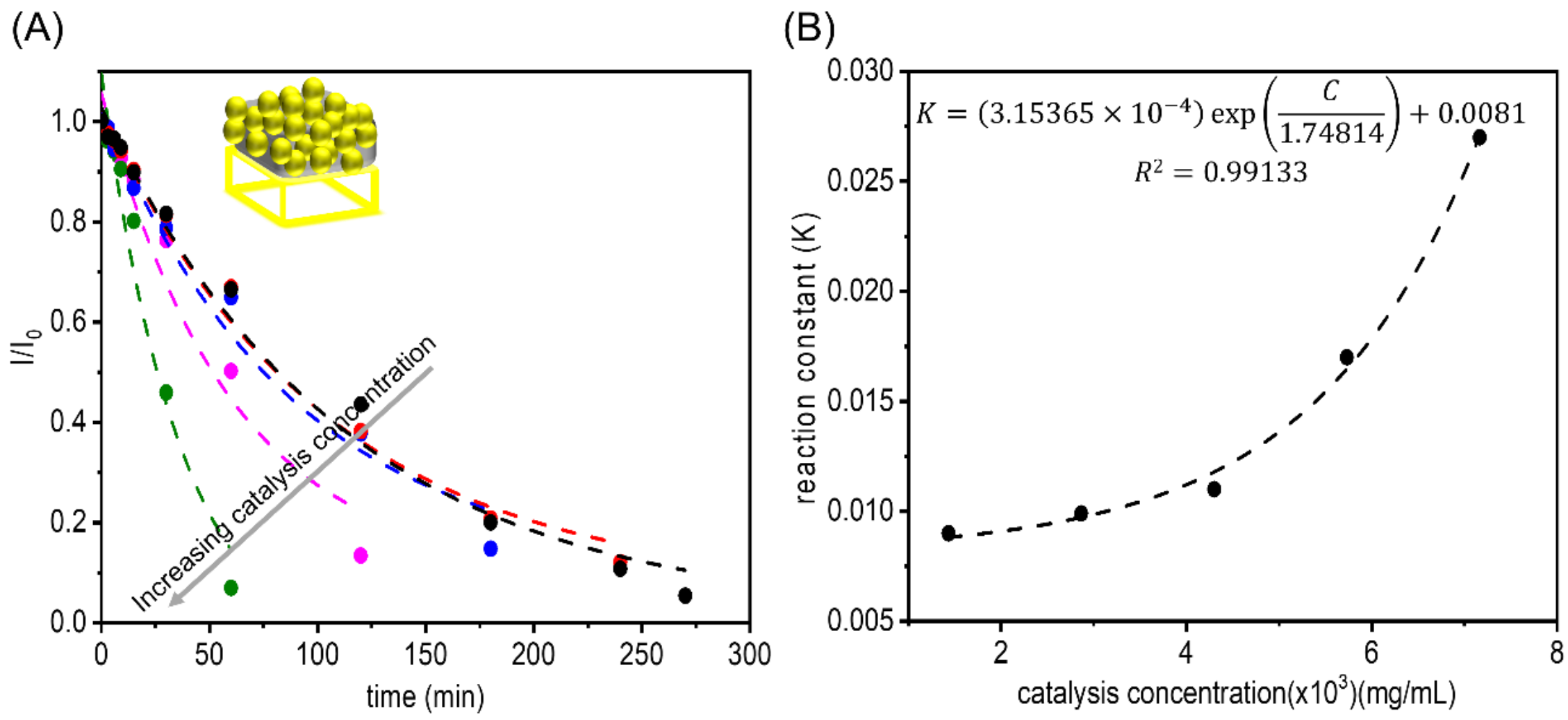

Figure S22 The reduction of 4-NP was carried out using Au-Ag flower + Au-Ag cage nanocrystal as catalysts. (A) Concentration change (I/I_0, I: concentration of 4-NP during the reaction, I_0: initial concentration of 4-NP) over time. Solid dots represent experimental data. Dashed lines represent the fitted curve of the first-order reaction mechanism ($\frac{I}{I_0} = y_0 + A \times e^{-(t/t_0)}$ and t_0= 1/reaction constant (K)). (B) The reaction constant (K) varies with catalyst concentration.

Table S8 The reaction constant (K) of reduction of 4-NP was carried out using Au-Ag flower + Au-Ag cage nanocrystal as catalysts which resulted from $\frac{I}{I_0} = y_0 + A \times e^{-(t/t_0)}$.

Concentration of catalysts($\times 10^3$ mg/mL)	Fitting function : $I/I_0 = y_0 + A*\exp(-t/t_0)$, $K=1/t_0$				
	y_0	A	t_0	$K=1/t_0$	R-square
1.433	0.016	1.012	110.808	0.0090	0.987
2.866	0.067	0.961	101.581	0.0098	0.989
4.300	0.014	0.922	88.993	0.0112	0.976
5.732	0.096	0.961	59.484	0.0168	0.955
7.165	-0.089	1.185	36.997	0.0270	0.946

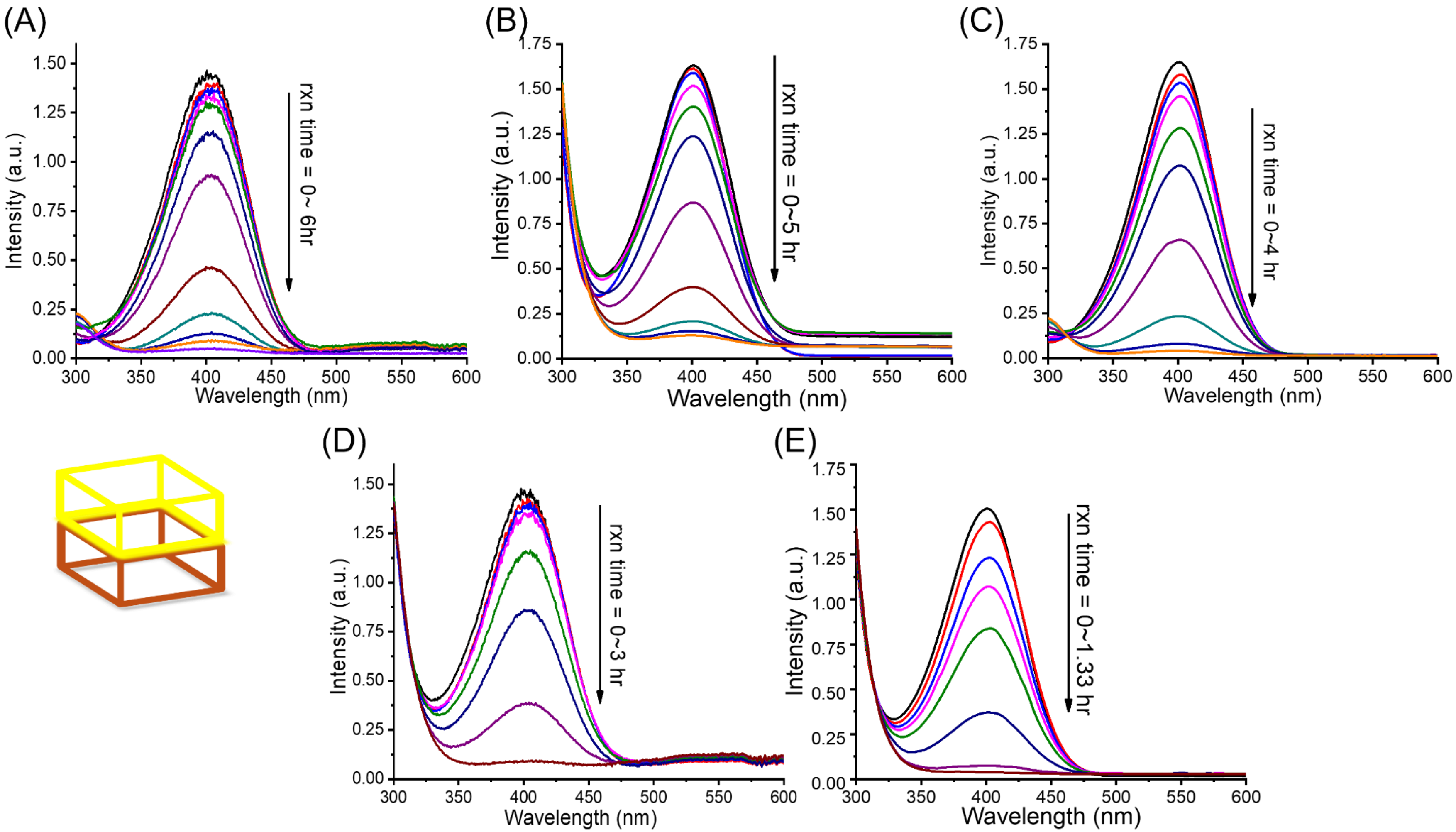

Figure S23 The reduction of 4-NP was carried out using Pt-Ag cage + Au-Ag cage nanocrystals as catalysts. The 4-NP concentration varied with reaction time under different catalyst concentrations. The catalyst concentration increased sequentially from (A) to (E).

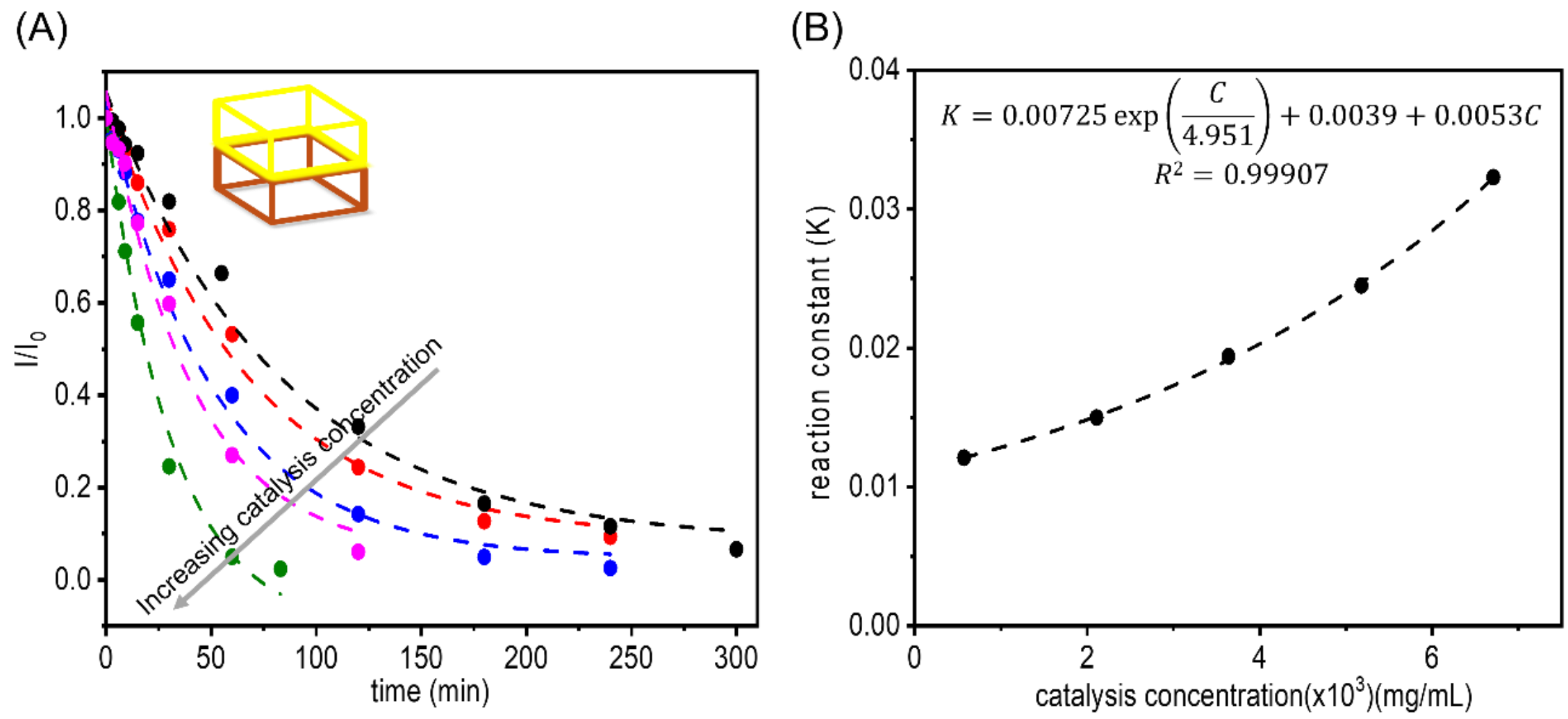

Figure S24 The reduction of 4-NP was carried out using Pt-Ag cage + Au-Ag cage nanocrystal as catalysts. (A) Concentration change (I/I_0, I: concentration of 4-NP during the reaction, I_0: initial concentration of 4-NP) over time. Solid dots represent experimental data. Dashed lines represent the fitted curve of the first-order reaction mechanism ($\frac{I}{I_0} = y_0 + A \times e^{-(t/t_0)}$ and t_0 = 1/reaction constant (K)). (B) The reaction constant (K) varies with catalyst concentration.

Table S9 The reaction constant (K) of reduction of 4-NP was carried out using Pt-Ag cage + Au-Ag cage nanocrystal as catalysts which resulted from $\frac{I}{I_0} = y_0 + A \times e^{-(t/t_0)}$.

Concentration of catalysts($\times 10^3$ mg/mL)	Fitting function : $I/I_0 = y_0 + A*\exp(-t/t_0)$, $K=1/t_0$				
	y_0	A	t_0	$K=1/t_0$	R-square
0.608	0.080	0.978	82.498	0.0121	0.966
2.148	0.089	0.960	66.859	0.0150	0.991
3.678	0.046	0.966	51.427	0.0194	0.993
5.218	0.051	1.006	40.797	0.0245	0.981
6.748	-0.106	1.112	30.908	0.0323	0.988

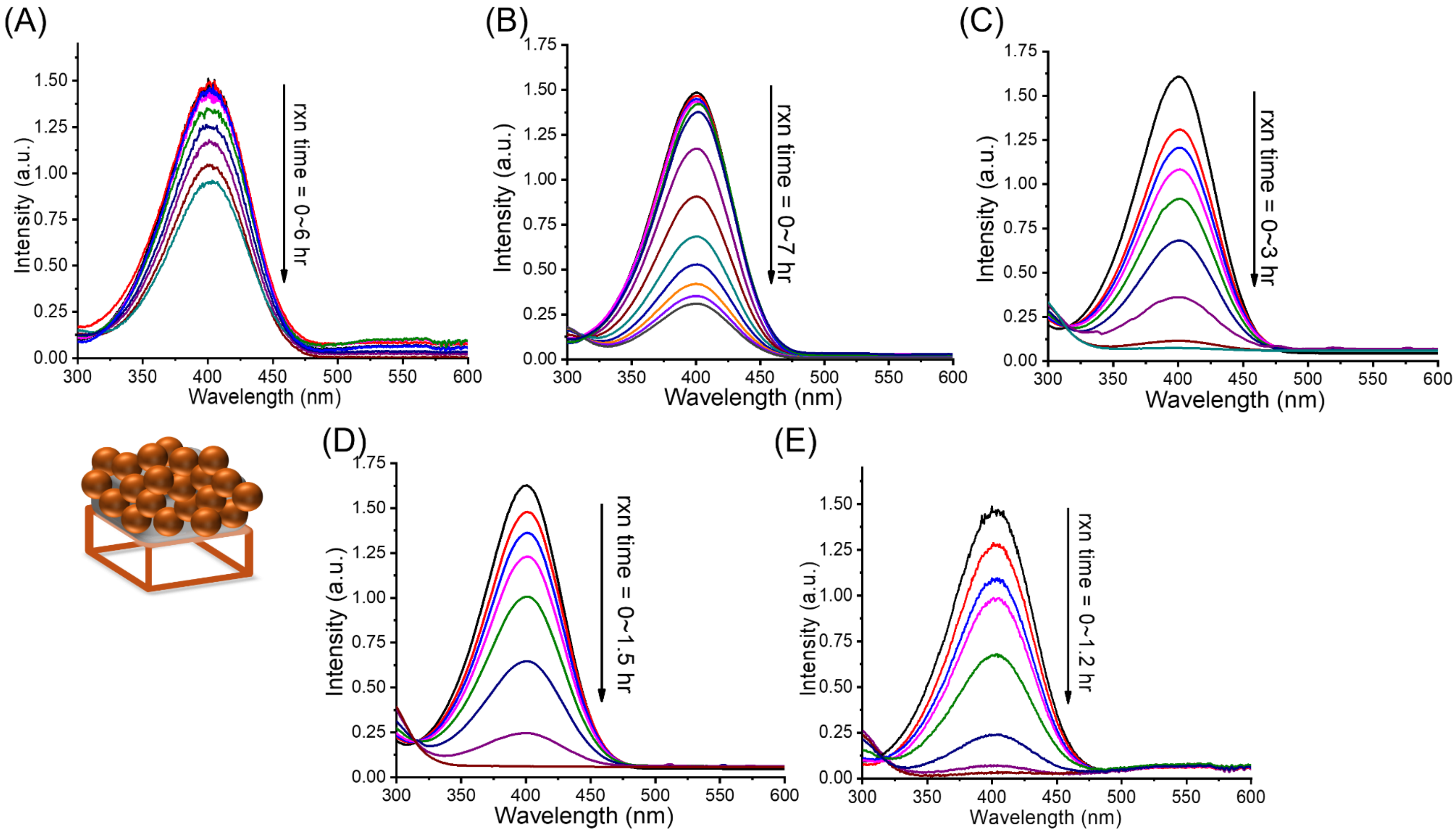

Figure S25 The reduction of 4-NP was carried out using Pt-Ag flower + Pt-Ag cage nanocrystals as catalysts. The 4-NP concentration varied with reaction time under different catalyst concentrations. The catalyst concentration increased sequentially from (A) to (E).

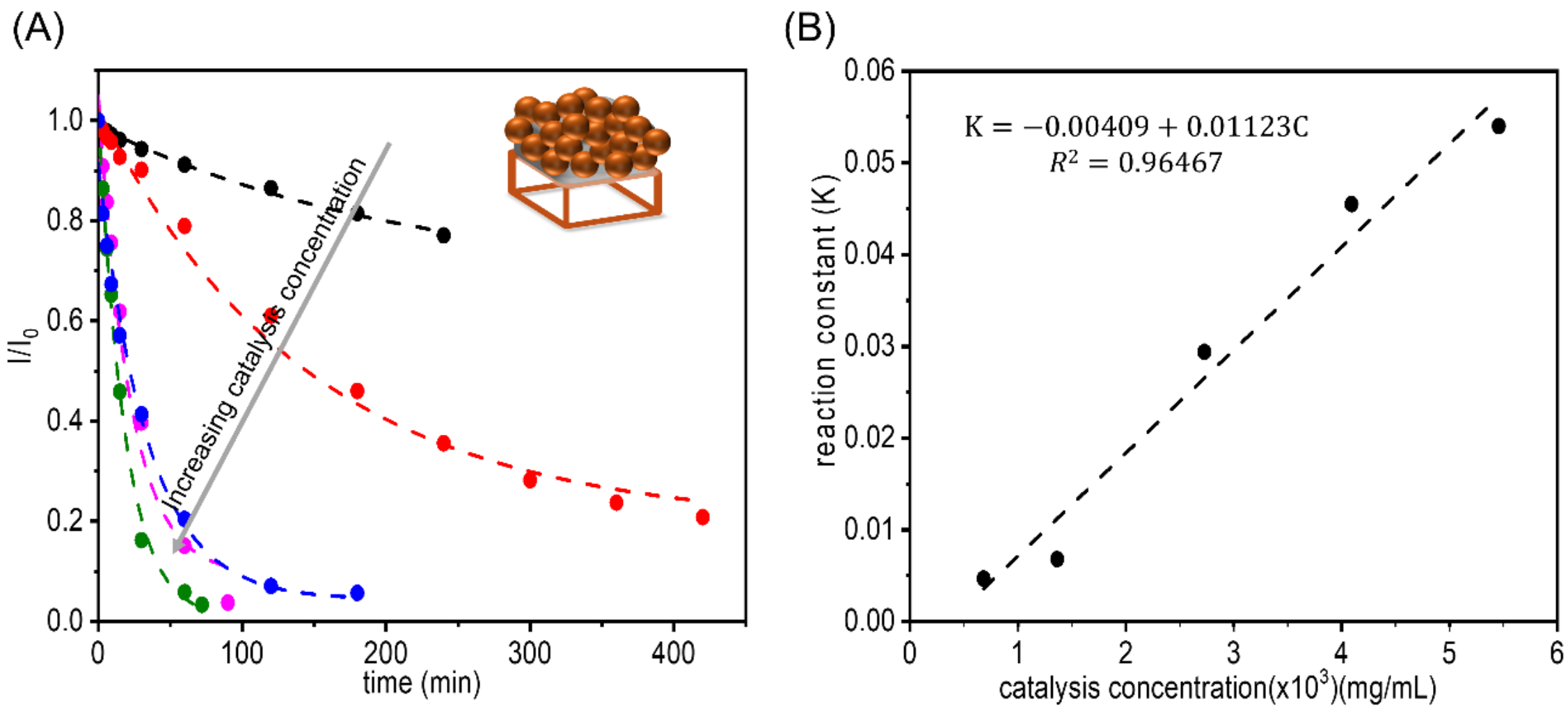

Figure S26 The reduction of 4-NP was carried out using Pt-Ag flower + Pt-Ag cage nanocrystal as catalysts. (A) Concentration change (I/I_0, I: concentration of 4-NP during the reaction, I_0: initial concentration of 4-NP) over time. Solid dots represent experimental data. Dashed lines represent the fitted curve of the first-order reaction mechanism ($\frac{I}{I_0} = y_0 + A \times e^{-(t/t_0)}$ and t_0 = 1/reaction constant (K)). (B) The reaction constant (K) varies with catalyst concentration.

Table S10 The reaction constant (K) of reduction of 4-NP was carried out using Pt-Ag flower + Pt-Ag cage nanocrystal as catalysts which resulted from $\frac{I}{I_0} = y_0 + A \times e^{-(t/t_0)}$.

Concentration of catalysts($\times 10^3$ mg/mL)	Fitting function : $I/I_0 = y_0 + A*\exp(-t/t_0)$, $K=1/t_0$				
	y_0	A	t_0	$K=1/t_0$	R-square
0.682	0.677	0.312	214.225	0.0047	0.992
1.364	0.194	0.827	145.898	0.0069	0.990
2.728	0.044	0.870	34.021	0.0294	0.986
4.092	0.094	0.956	21.994	0.0455	0.981
5.456	0.006	1.010	18.445	0.0542	0.996

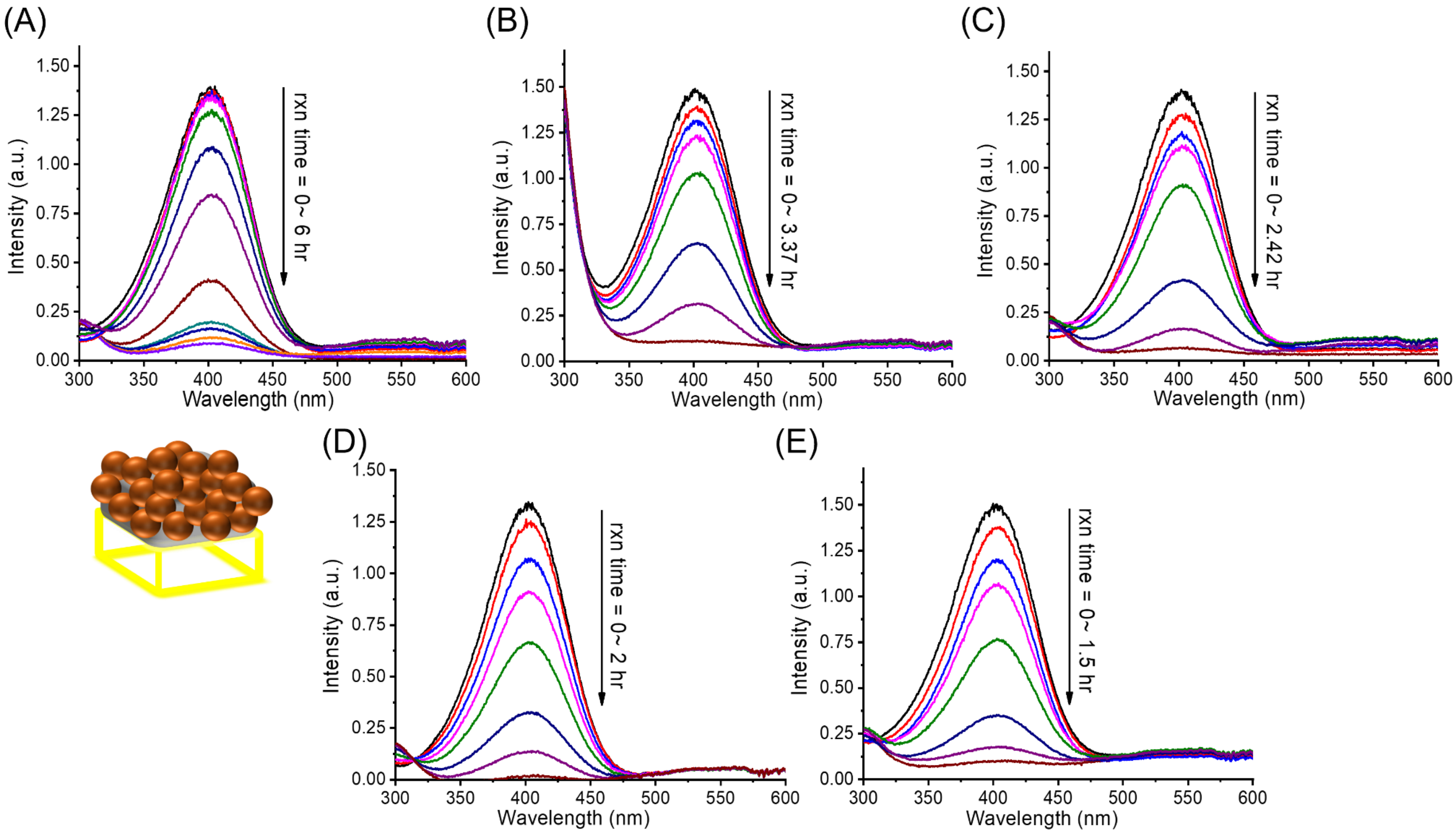

Figure S27 The reduction of 4-NP was carried out using Au-Ag cage + Pt-Ag flower nanocrystals as catalysts. The 4-NP concentration varied with reaction time under different catalyst concentrations. The catalyst concentration increased sequentially from (A) to (E).

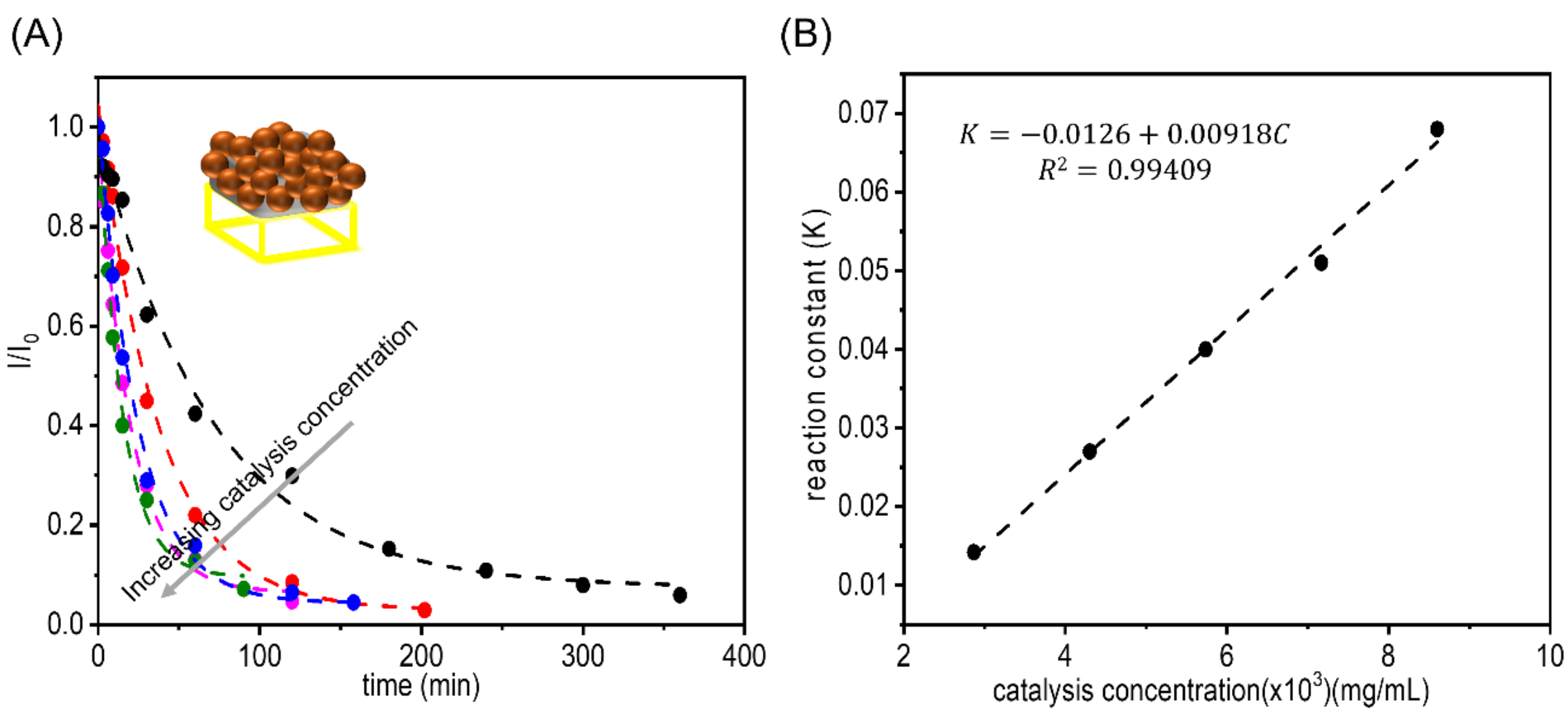

Figure S28 The reduction of 4-NP was carried out using Au-Ag cage + Pt-Ag flower nanocrystal as catalysts. (A) Concentration change (I/I_0, I: concentration of 4-NP during the reaction, I_0: initial concentration of 4-NP) over time. Solid dots represent experimental data. Dashed lines represent the fitted curve of the first-order reaction mechanism ($\frac{I}{I_0} = y_0 + A \times e^{-(t/t_0)}$ and t_0 = 1/reaction constant (K)). (B) The reaction constant (K) varies with catalyst concentration.

Table S11 The reaction constant (K) of reduction of 4-NP was carried out using Au-Ag cage + Pt-Ag flower nanocrystal as catalysts which resulted from $\frac{I}{I_0} = y_0 + A \times e^{-(t/t_0)}$.

Concentration of catalysts($\times 10^3$ mg/mL)	Fitting function : $I/I_0 = y_0 + A*\exp(-t/t_0)$, $K=1/t_0$				
	y_0	A	t_0	$K=1/t_0$	R-square
2.867	0.075	0.910	70.364	0.0142	0.991
4.301	0.029	1.016	37.079	0.0270	0.994
5.734	0.053	0.926	21.106	0.0473	0.997
7.168	0.093	0.963	19.419	0.0515	0.991
8.601	0.096	0.914	14.796	0.0676	0.994

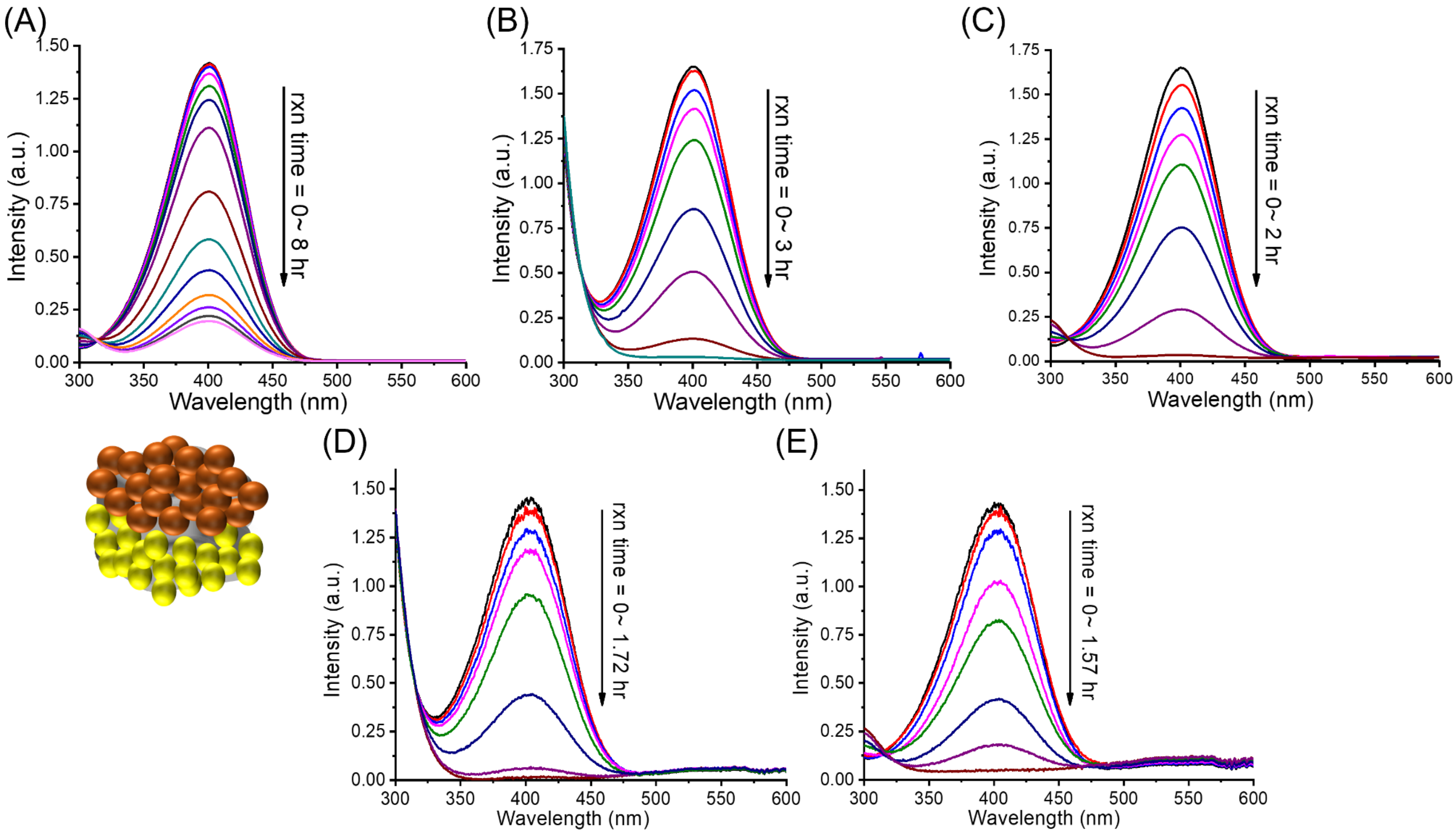

Figure S29 The reduction of 4-NP was carried out using Pt-Ag flower + Au-Ag flower nanocrystals as catalysts. The 4-NP concentration varied with reaction time under different catalyst concentrations. The catalyst concentration increased sequentially from (A) to (E).

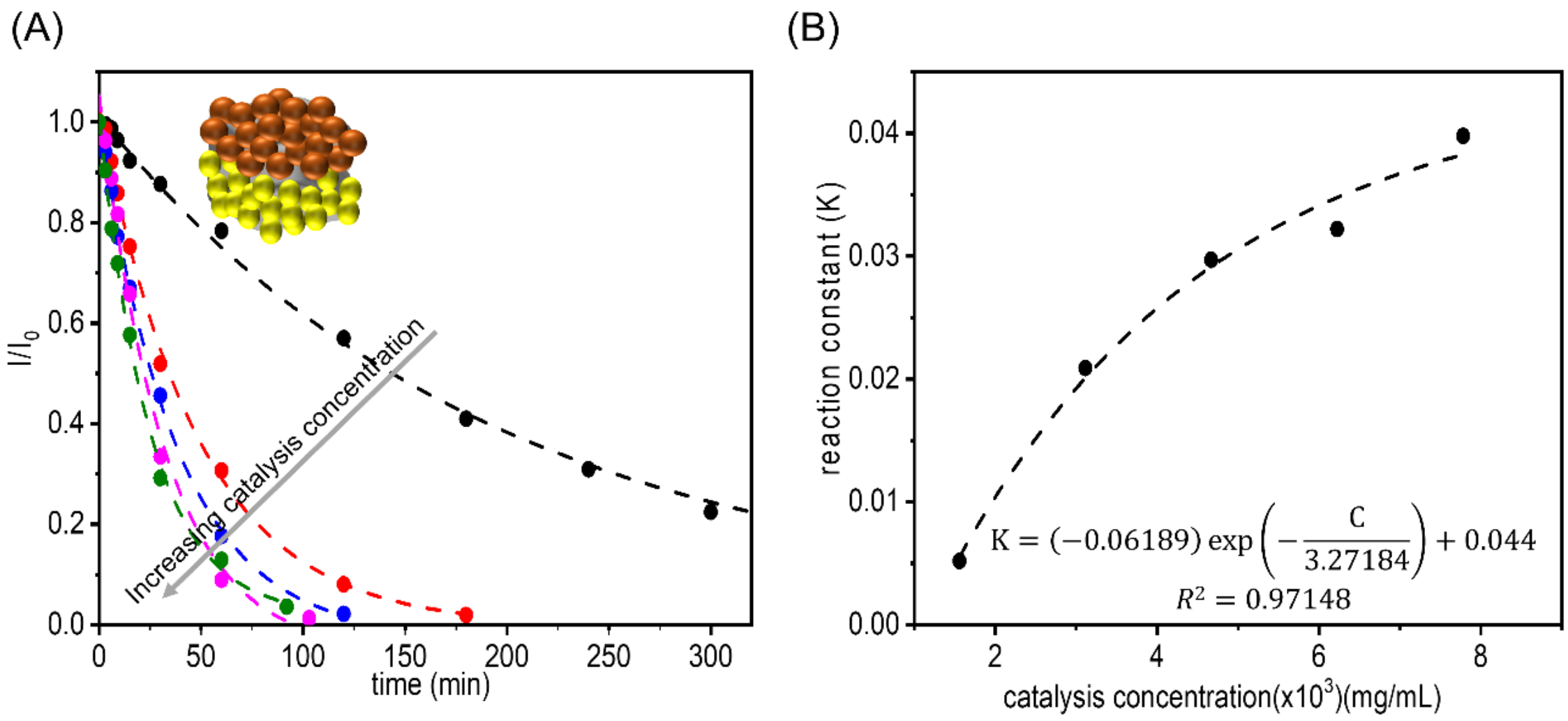

Figure S30 The reduction of 4-NP was carried out using Pt-Ag flower + Au-Ag flower nanocrystal as catalysts. (A) Concentration change (I/I_0, I: concentration of 4-NP during the reaction, I_0: initial concentration of 4-NP) over time. Solid dots represent experimental data. Dashed lines represent the fitted curve of the first-order reaction mechanism ($\frac{I}{I_0} = y_0 + A \times e^{-(t/t_0)}$ and t_0 = 1/reaction constant (K)). (B) The reaction constant (K) varies with catalyst concentration.

Table S12 The reaction constant (K) of reduction of 4-NP was carried out using Pt-Ag flower + Au-Ag flower nanocrystal as catalysts which resulted from $\frac{I}{I_0} = y_0 + A \times e^{-(t/t_0)}$.

Concentration of catalysts($\times 10^3$ mg/mL)	Fitting function : $I/I_0 = y_0 + A*\exp(-t/t_0)$, $K=1/t_0$				
	y_0	A	t_0	$K=1/t_0$	R-square
1.556	0.042	0.970	191.538	0.0052	0.998
3.112	-0.002	1.031	47.847	0.0209	0.997
4.668	-0.026	1.033	33.670	0.0297	0.999
6.224	-0.055	1.107	31.055	0.0322	0.990
7.78	0.019	0.989	25.135	0.0398	0.997

Table S13 The catalytic and photocatalytic performances of Janus-like nanocrystals were compared with those of bimetallic nanocrystals Au-Ag and Pt-Ag reported in the literatures.

Catalysts type		Weight of catalyst (mg)	4-NP (mole)	$NaBH_4$(mole)	4-NP /weight of catalyst (mole/mg)	Overall rxn time	Reference
Janus-like NC	Catalyst	0.018	2.25×10^{-7}	6×10^{-5}	1.25×10^{-5}	80~120min	This manuscript
	Photocatalyst					< 60 min	
Pt/Ag bimetallic NP (catalyst)		4.5	1.5×10^{-6}	4.49×10^{-5}	0.033×10^{-5}	~5 min	a
Pt/Ag bimetallic NP (Photocatalyst)		10	3×10^{-7}	1×10^{-7}	0.003×10^{-5}	~5 min	b
Pt/Ag bimetallic NP (Photocatalyst)		0.15	5×10^{-7}	1×10^{-4}	0.33×10^{1}	~5 min	c
Au/Ag bimetallic NP (catalyst)		1	2.77×10^{-7}	6.67×10^{-6}	0.003×10^{-5}	~30 min	d
Au/Ag bimetallic NP (Photocatalyst)		0.125	3×10^{-7}	2.6×10^{-2}	0.24×10^{-5}	~8 min	e

a. S. Li, F. Tang, H. Wang, J. Feng, Z. Jin, "Au-Ag and Pt-Ag bimetallic nanoparticles@halloysite nanotubes: morphological modulation, improvement othermal stability and catalytic performance" RSC Adv, 2018, 8, 10237-10245.

b. S.G. Khasevani, M. Taheri, M.R. Gholami, "Green synthesis of Ag-Pt bimetallic nanoparticles supported on the Metal-Organic framework (MOF)–Derived metal oxides (γ-Fe_2O_3/CuO) nanocomposite as a reusable heterogeneous nanocatalyst and nanophotocatalyst"Mater. Chem. Phys., 2021, 261 Article 124218.

c. Meng Ding, Bing-Qian Shan, Bo Peng, Jia-Feng Zhou and Kun Zhang, "Dynamic Pt–OH−·H2O–Ag species mediate coupled electron and proton transfer for catalytic hydride reduction of 4-nitrophenol at the confined nanoscale interface", Phys. Chem. Chem. Phys., 2022,24, 7923-793. N. Arora,

d. A. Mehta, A. Mishra and S. Basu, 4-Nitrophenol reduction catalysed by Au–Ag bimetallic nanoparticles supported on LDH: homogeneous vs. heterogeneous catalysis, Appl. Clay Sci., 2018, 151, 1–9.

e. Y. Su, P. Gao, G. Zhang, Y. Zhou, L. Shi, J. Wu, W. Liang, S. Shuang, Y. Zhang. "Multifunctional Au-Ag NCs for luminescence and colorimetric double signal sensing of H2S and catalytic reduction of nitrophenol " Talanta, 282 (2025), 127022

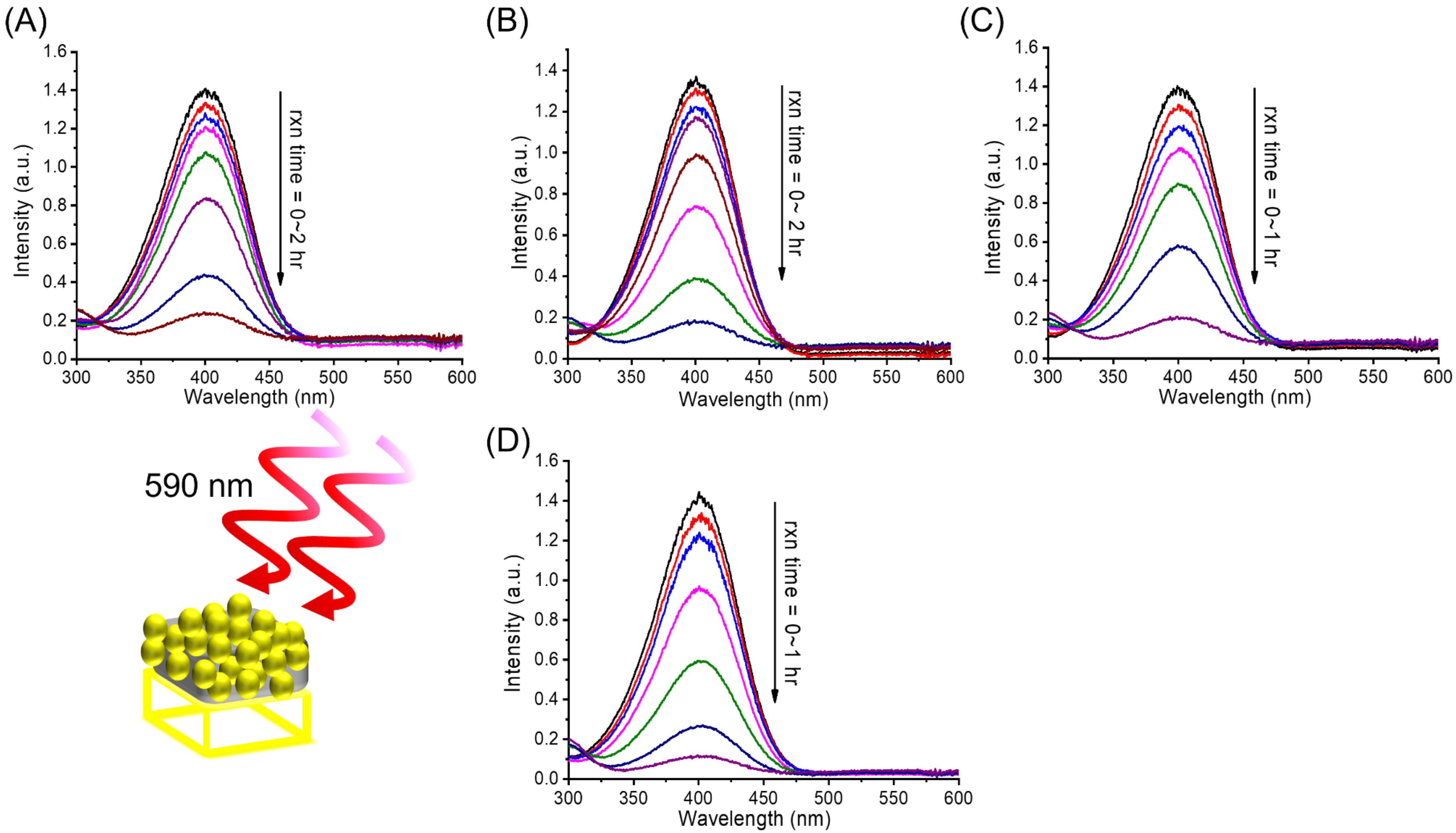

Figure S31 Au-Ag flower + Au-Ag cage nanocrystals were used as photocatalysts to carry out the reduction reaction of 4-NP under irradiation at a wavelength of 590 nm (on-plasmonic mode of this Janus-like metallic nanocrystal). The 4-NP concentration varied with reaction time under different catalyst concentrations. The catalyst concentration increased sequentially from (A) to (D).

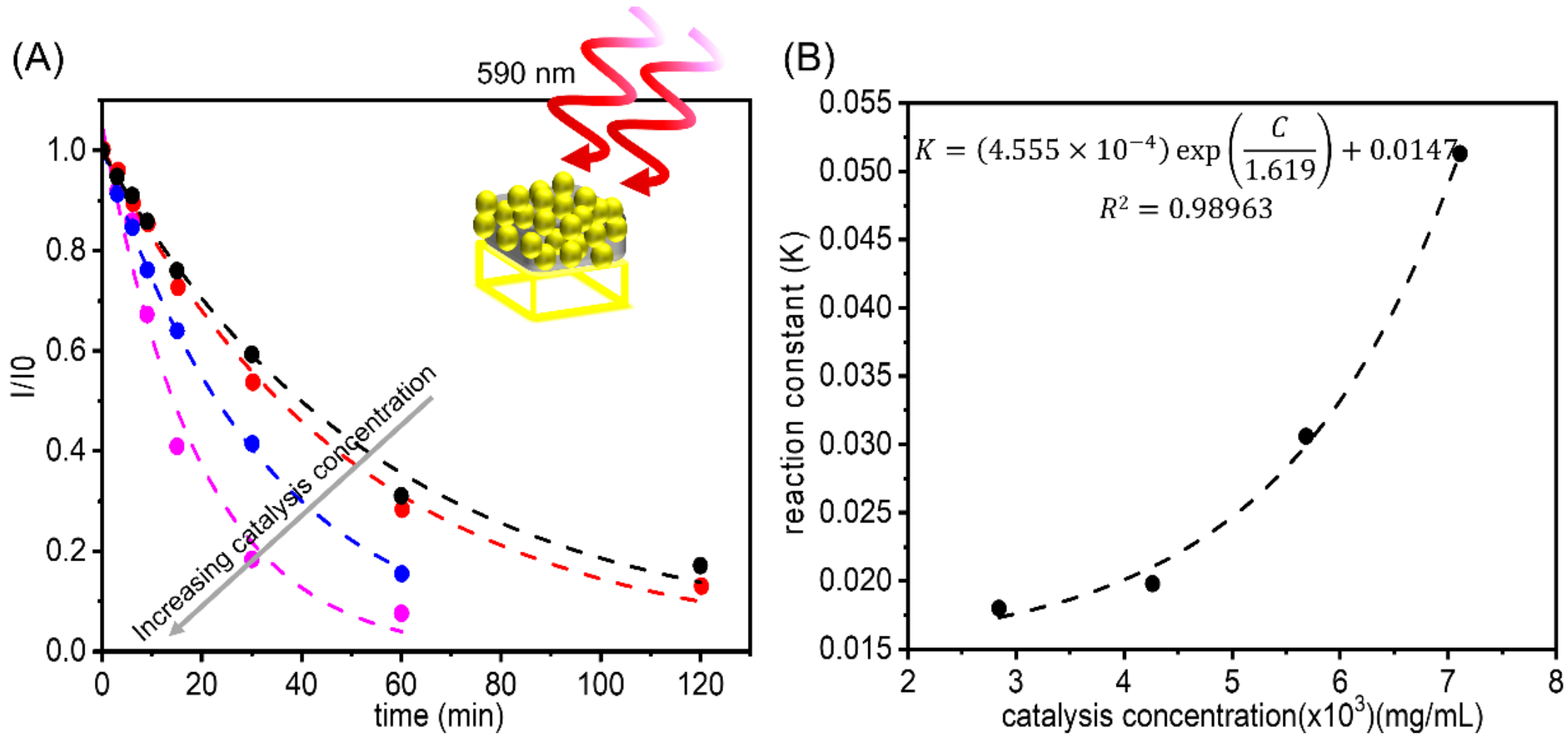

Figure S32 Au-Ag flower + Au-Ag cage nanocrystals were used as photocatalysts to carry out the reduction reaction of 4-NP under irradiation at a wavelength of 590 nm (on-plasmonic mode of this Janus-like metallic nanocrystal). (A) Concentration change (I/I_0, I: concentration of 4-NP during the reaction, I_0: initial concentration of 4-NP) over time. Solid dots represent experimental data. Dashed lines represent the fitted curve of the first-order reaction mechanism ($\frac{I}{I_0} = y_0 + A \times e^{-(t/t_0)}$ and t_0 = 1/reaction constant (K)). (B) The reaction constant (K) varies with catalyst concentration.

Table S14 The reaction constant (K) of reduction of 4-NP was carried out using Au-Ag flower + Au-Ag cage nanocrystal as photocatalysts at a wavelength of 590 nm (on-plasmonic mode of this Janus-like metallic nanocrystal) which resulted from $\frac{I}{I_0} = y_0 + A \times e^{-(t/t_0)}$.

Concentration of catalysts($\times 10^3$ mg/mL)	Fitting function : $I/I_0 = y_0 + A*\exp(-t/t_0)$, $K=1/t_0$				
	y_0	A	t_0	$K=1/t_0$	R-square
2.842	0.025	0.973	55.554	0.0180	0.994
4.263	0.004	0.996	50.507	0.0198	0.996
5.684	0.003	1.003	32.677	0.0306	0.999
7.109	-0.009	1.061	19.481	0.0513	0.972

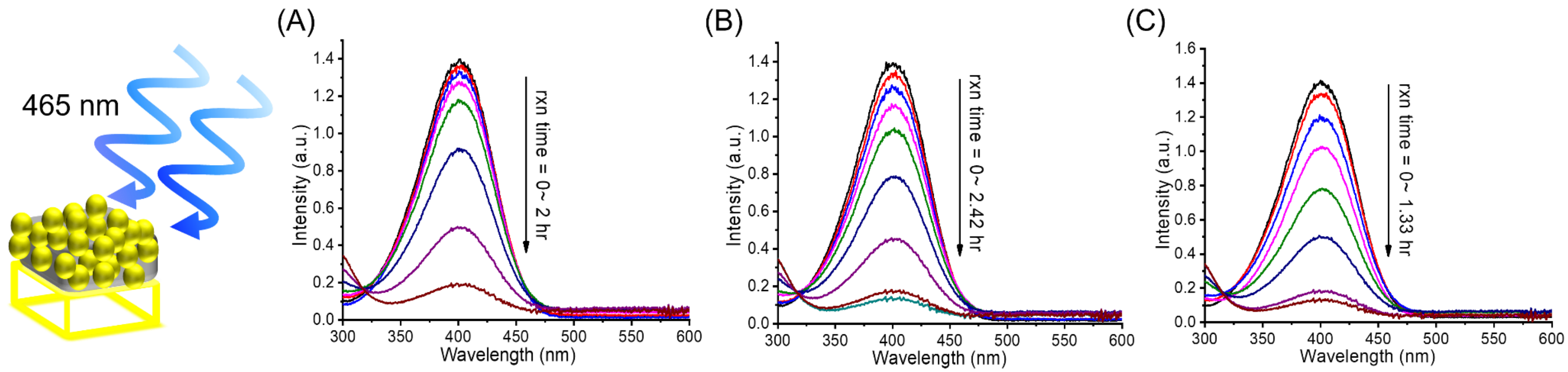

Figure S33 Au-Ag flower + Au-Ag cage nanocrystals were used as photocatalysts to carry out the reduction reaction of 4-NP under irradiation at a wavelength of 465 nm (off-plasmonic mode of this Janus-like metallic nanocrystal). The 4-NP concentration varied with reaction time under different catalyst concentrations. The catalyst concentration increased sequentially from (A) to (C).

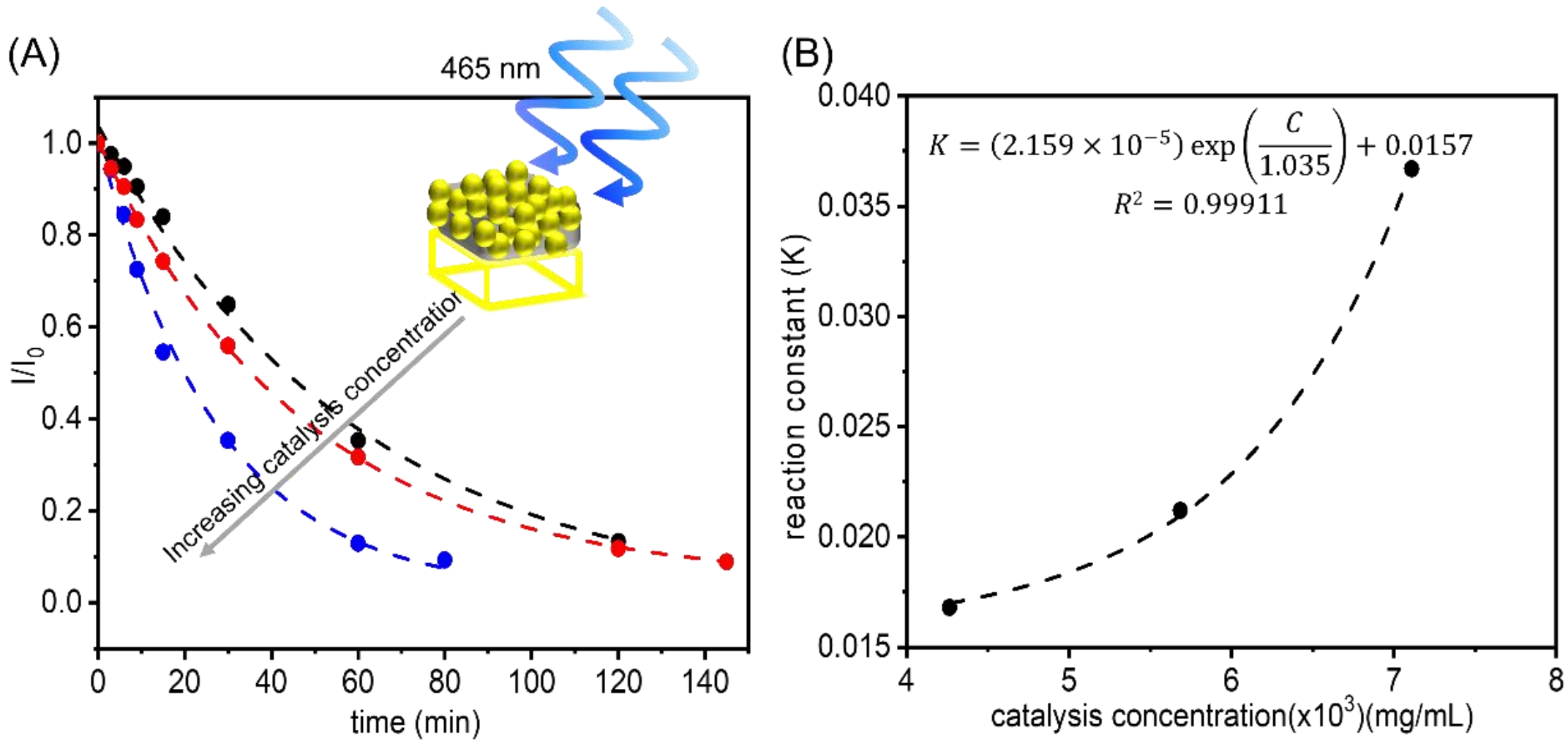

Figure S34 Au-Ag flower + Au-Ag cage nanocrystals were used as photocatalysts to carry out the reduction reaction of 4-NP under irradiation at a wavelength of 465 nm (off-plasmonic mode of this Janus-like metallic nanocrystal). (A) Concentration change (I/I_0, I: concentration of 4-NP during the reaction, I_0: initial concentration of 4-NP) over time. Solid dots represent experimental data. Dashed lines represent the fitted curve of the first-order reaction mechanism ($\frac{I}{I_0} = y_0 + A \times e^{-(t/t_0)}$ and t_0 = 1/reaction constant (K)). (B) The reaction constant (K) varies with catalyst concentration.

Table S15 The reaction constant (K) of reduction of 4-NP was carried out using Au-Ag flower + Au-Ag cage nanocrystal as photocatalysts at a wavelength of 465 nm (off-plasmonic mode of this Janus-like metallic nanocrystal) which resulted from $\frac{I}{I_0} = y_0 + A \times e^{-(t/t_0)}$.

Concentration of catalysts($\times 10^3$ mg/mL)	Fitting function : $I/I_0 = y_0 + A*\exp(-t/t_0)$, $K=1/t_0$				
	y_0	A	t_0	$K=1/t_0$	R-square
4.263	-0.002	1.041	59.571	0.0168	0.993
5.684	0.045	0.961	47.233	0.0212	0.999
7.109	0.023	0.992	27.244	0.0367	0.994

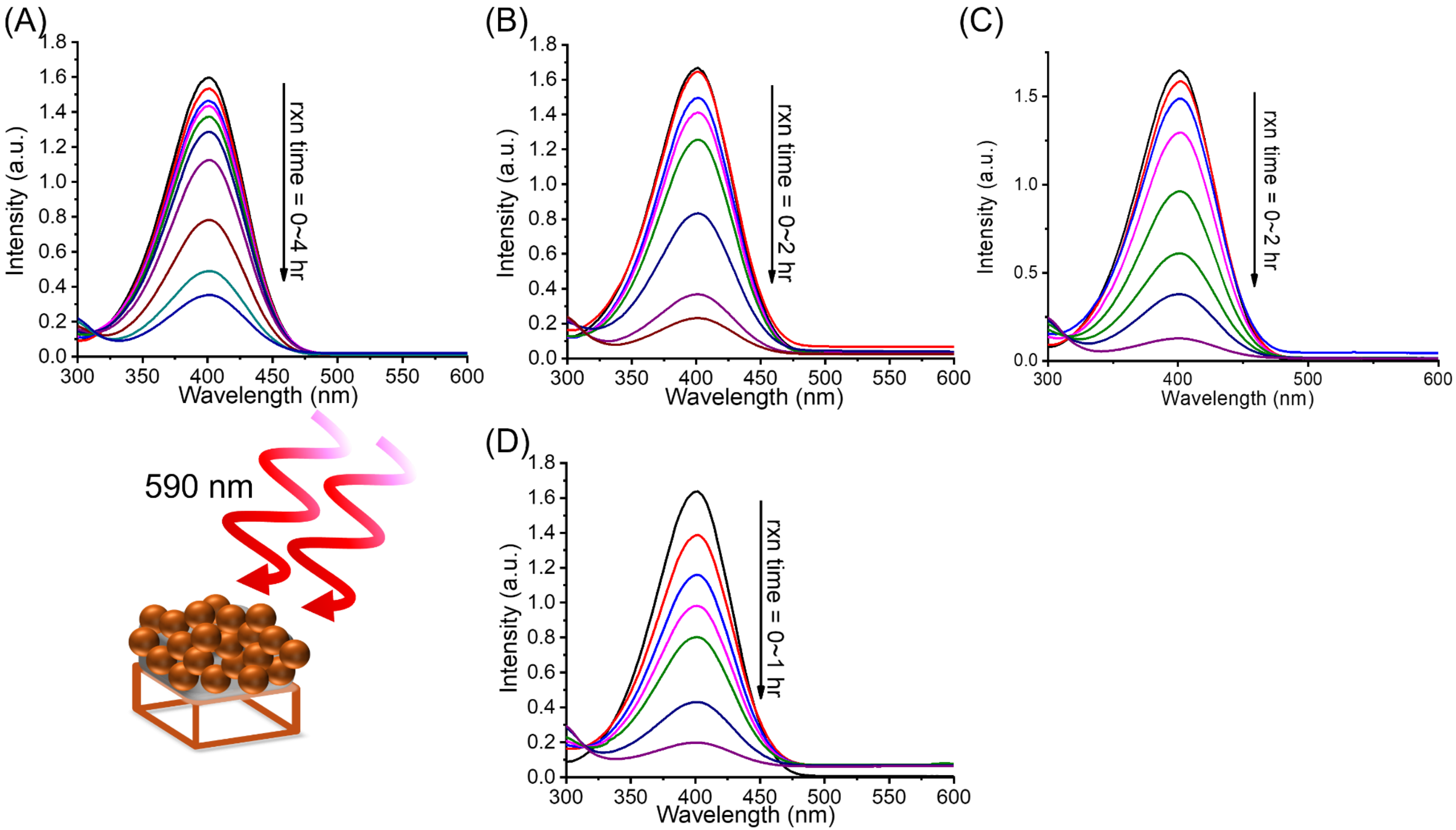

Figure S35 Pt-Ag flower + Pt-Ag cage nanocrystals were used as photocatalysts to carry out the reduction reaction of 4-NP under irradiation at a wavelength of 590 nm (on-plasmonic mode of this Janus-like metallic nanocrystal). The 4-NP concentration varied with reaction time under different catalyst concentrations. The catalyst concentration increased sequentially from (A) to (D).

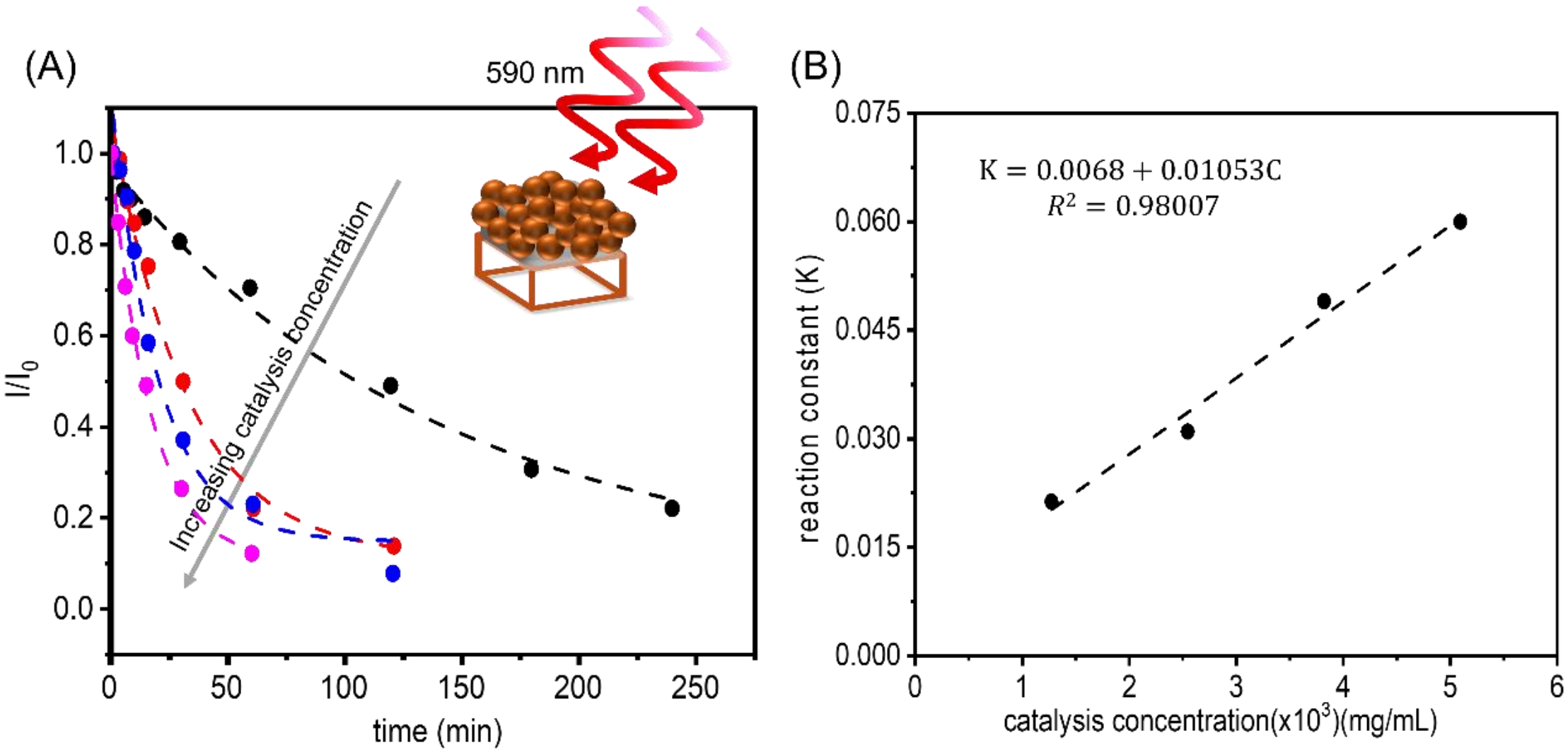

Figure S36 Pt-Ag flower + Pt-Ag cage nanocrystals were used as photocatalysts to carry out the reduction reaction of 4-NP under irradiation at a wavelength of 590 nm (on-plasmonic mode of this Janus-like metallic nanocrystal). (A) Concentration change (I/I_0, I: concentration of 4-NP during the reaction, I_0: initial concentration of 4-NP) over time. Solid dots represent experimental data. Dashed lines represent the fitted curve of the first-order reaction mechanism ($\frac{I}{I_0} = y_0 + A \times e^{-(t/t_0)}$ and t_0 = 1/reaction constant (K)). (B) The reaction constant (K) varies with catalyst concentration.

Table S16 The reaction constant (K) of reduction of 4-NP was carried out using Pt-Ag flower + Pt-Ag cage nanocrystal as photocatalysts at a wavelength of 590 nm (on-plasmonic mode of this Janus-like metallic nanocrystal) which resulted from $\frac{I}{I_0} = y_0 + A \times e^{-(t/t_0)}$.

Concentration of catalysts($\times 10^3$ mg/mL)	Fitting function : $I/I_0 = y_0 + A*\exp(-t/t_0)$, $K=1/t_0$				
	y_0	A	t_0	$K=1/t_0$	R-square
1.274	-0.081	1.143	46.948	0.0213	0.983
2.547	0.113	0.942	32.287	0.0309	0.989
3.821	0.147	0.935	20.108	0.0497	0.975
5.094	0.106	0.884	16.763	0.0597	0.997

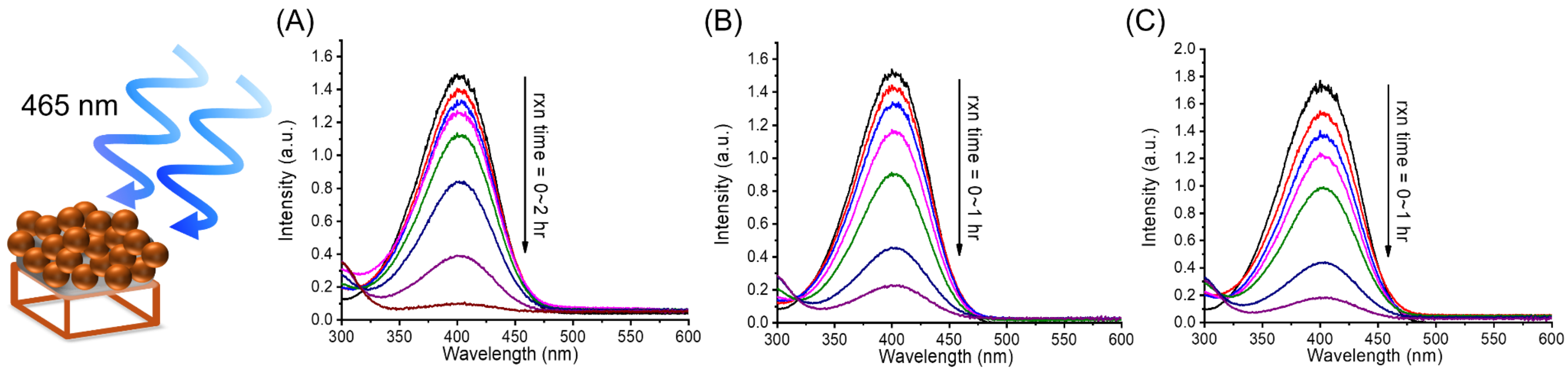

Figure S37 Pt-Ag flower + Pt-Ag cage nanocrystals were used as photocatalysts to carry out the reduction reaction of 4-NP under irradiation at a wavelength of 465 nm (off-plasmonic mode of this Janus-like metallic nanocrystal). The 4-NP concentration varied with reaction time under different catalyst concentrations. The catalyst concentration increased sequentially from (A) to (C).

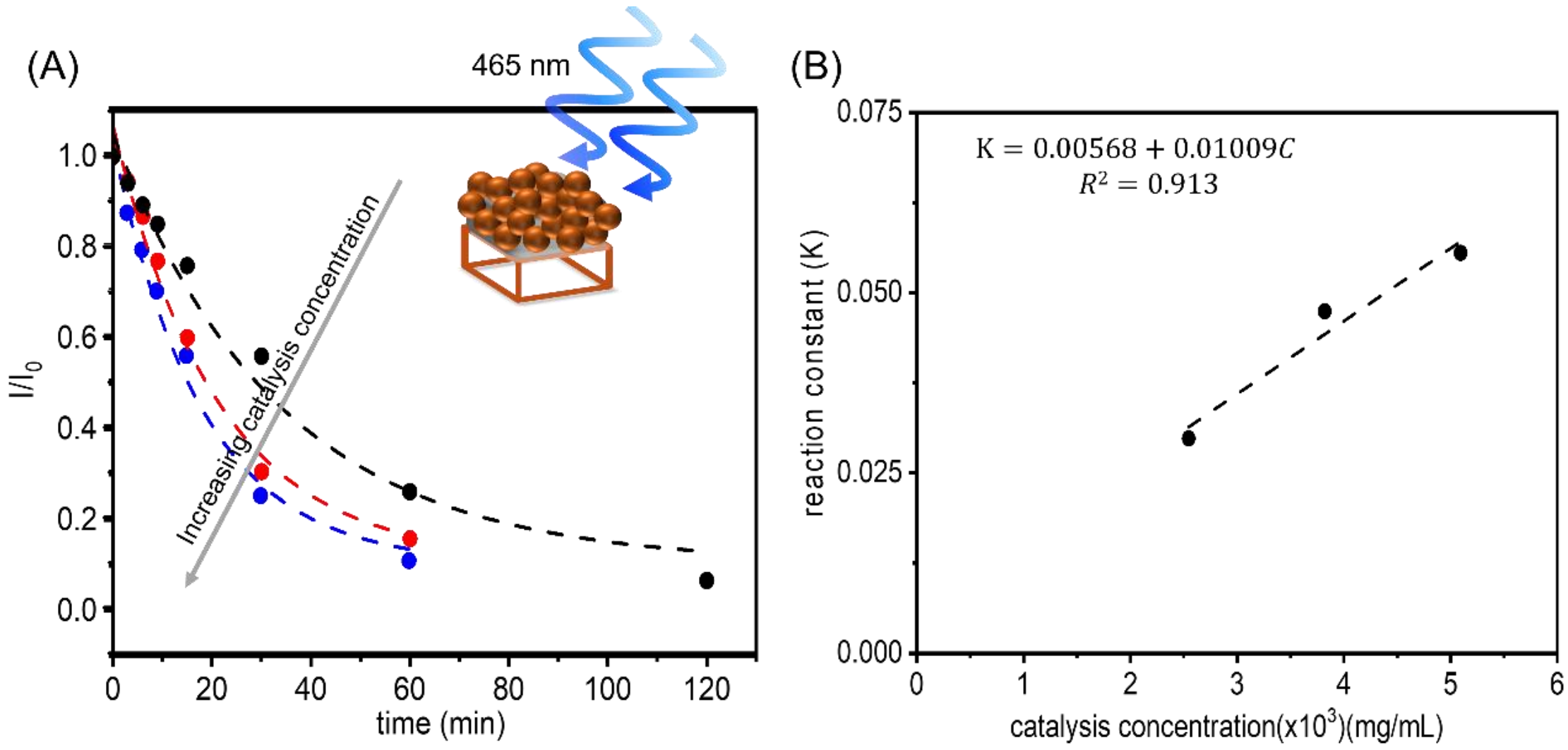

Figure S38 Pt-Ag flower + Pt-Ag cage nanocrystals were used as photocatalysts to carry out the reduction reaction of 4-NP under irradiation at a wavelength of 465 nm (off-plasmonic mode of this Janus-like metallic nanocrystal). (A) Concentration change (I/I_0, I: concentration of 4-NP during the reaction, I_0: initial concentration of 4-NP) over time. Solid dots represent experimental data. Dashed lines represent the fitted curve of the first-order reaction mechanism ($\frac{I}{I_0} = y_0 + A \times e^{-(t/t_0)}$ and t_0 = 1/reaction constant (K)). (B) The reaction constant (K) varies with catalyst concentration.

Table S17 The reaction constant (K) of reduction of 4-NP was carried out using Pt-Ag flower + Pt-Ag cage nanocrystal as photocatalysts at a wavelength of 465 nm (off-plasmonic mode of this Janus-like metallic nanocrystal) which resulted from $\frac{I}{I_0} = y_0 + A \times e^{-(t/t_0)}$.

Concentration of catalysts($\times 10^3$ mg/mL)	Fitting function : $I/I_0 = y_0 + A*\exp(-t/t_0)$, $K=1/t_0$				
	y_0	A	t_0	$K=1/t_0$	R-square
2.547	0.104	0.946	33.565	0.0298	0.978
3.821	0.103	0.956	21.054	0.0474	0.985
5.094	0.097	0.940	18.027	0.0554	0.986

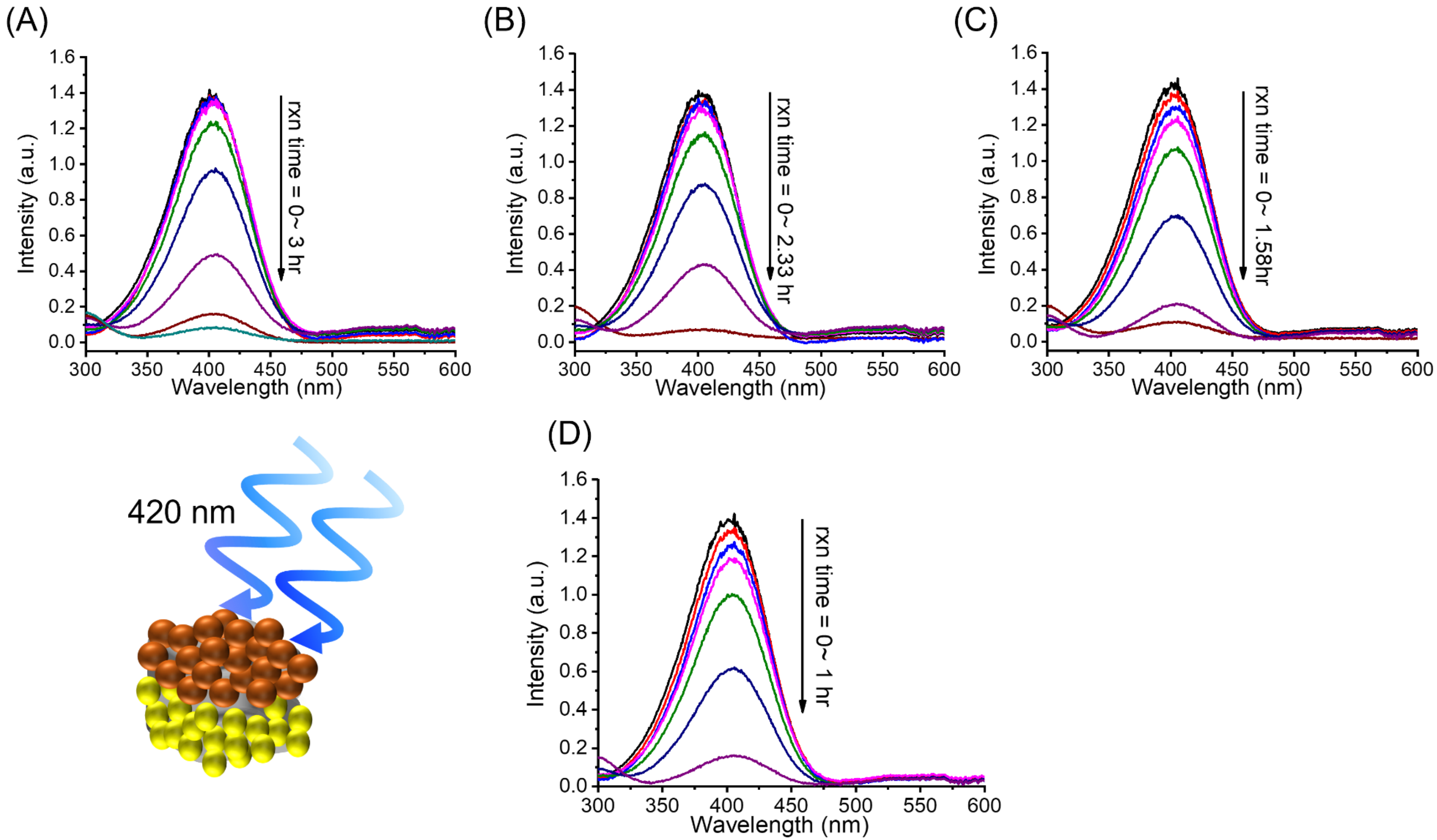

Figure S39 Pt-Ag flower + Au-Ag flower nanocrystals were used as photocatalysts to carry out the reduction reaction of 4-NP under irradiation at a wavelength of 420 nm (on-plasmonic mode of this Janus-like metallic nanocrystal). The 4-NP concentration varied with reaction time under different catalyst concentrations. The catalyst concentration increased sequentially from (A) to (D).

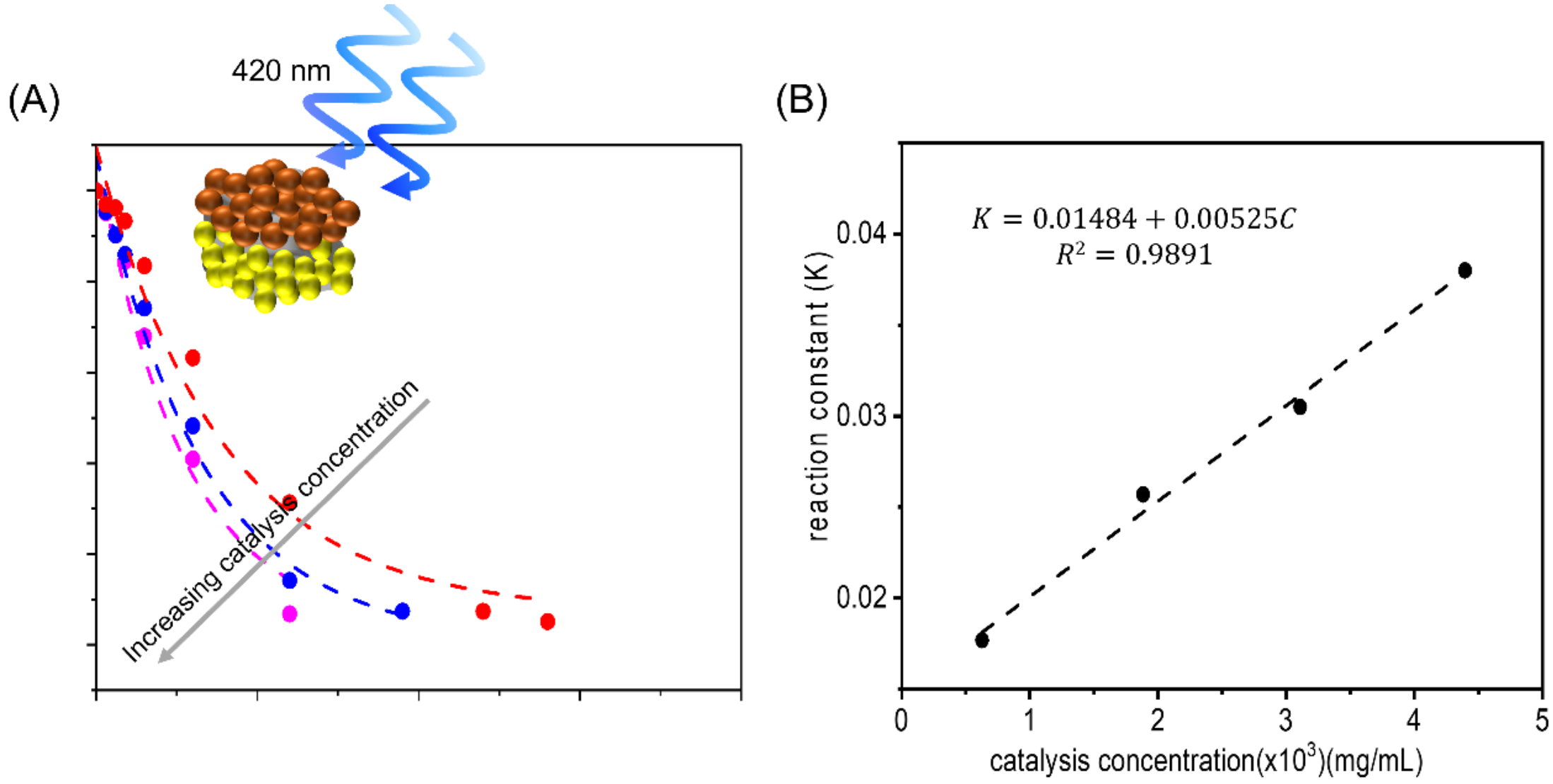

Figure S40 Pt-Ag flower + Au-Ag flower nanocrystals were used as photocatalysts to carry out the reduction reaction of 4-NP under irradiation at a wavelength of 420 nm (on-plasmonic mode of this Janus-like metallic nanocrystal). (A) Concentration change (I/I_0, I: concentration of 4-NP during the reaction, I_0: initial concentration of 4-NP) over time. Solid dots represent experimental data. Dashed lines represent the fitted curve of the first-order reaction mechanism ($\frac{I}{I_0} = y_0 + A \times e^{-(t/t_0)}$ and t_0 = 1/reaction constant (K)). (B) The reaction constant (K) varies with catalyst concentration.

Table S18 The reaction constant (K) of reduction of 4-NP was carried out using Pt-Ag flower + Au-Ag flower nanocrystal as photocatalysts at a wavelength of 420 nm (on-plasmonic mode of this Janus-like metallic nanocrystal) which resulted from $\frac{I}{I_0} = y_0 + A \times e^{-(t/t_0)}$.

Concentration of catalysts($\times 10^3$ mg/mL)	Fitting function : $I/I_0 = y_0 + A*\exp(-t/t_0)$, $K=1/t_0$				
	y_0	A	t_0	$K=1/t_0$	R-square
0.628	0.010	1.056	56.234	0.0178	0.988
1.884	0.071	1.025	38.916	0.0257	0.975
3.11	0.007	1.074	32.766	0.0305	0.983
4.396	0.035	1.049	26.505	0.0377	0.963

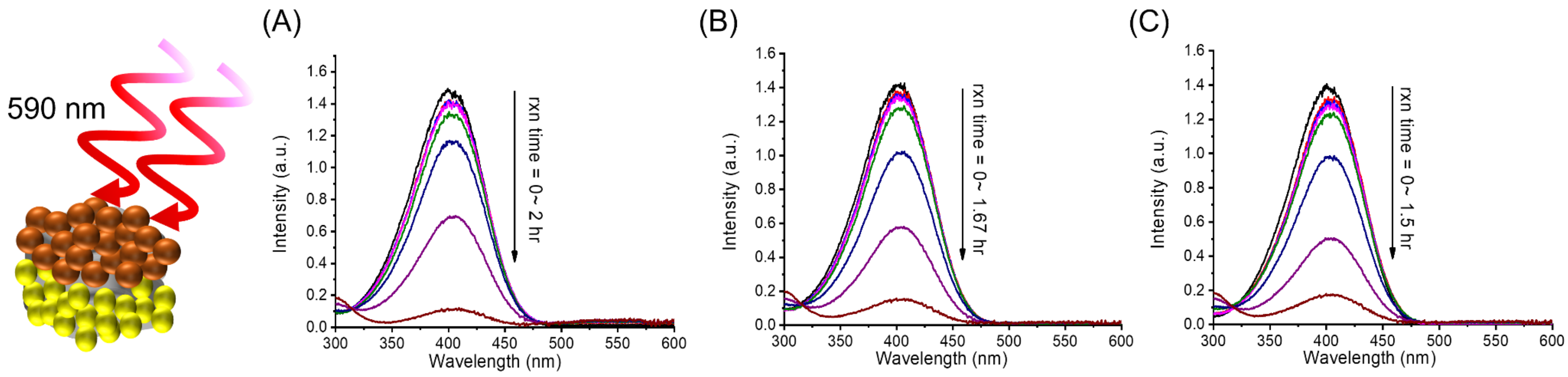

Figure S41 Pt-Ag flower + Au-Ag flower nanocrystals were used as photocatalysts to carry out the reduction reaction of 4-NP under irradiation at a wavelength of 590 nm (off-plasmonic mode of this Janus-like metallic nanocrystal). The 4-NP concentration varied with reaction time under different catalyst concentrations. The catalyst concentration increased sequentially from (A) to (C).

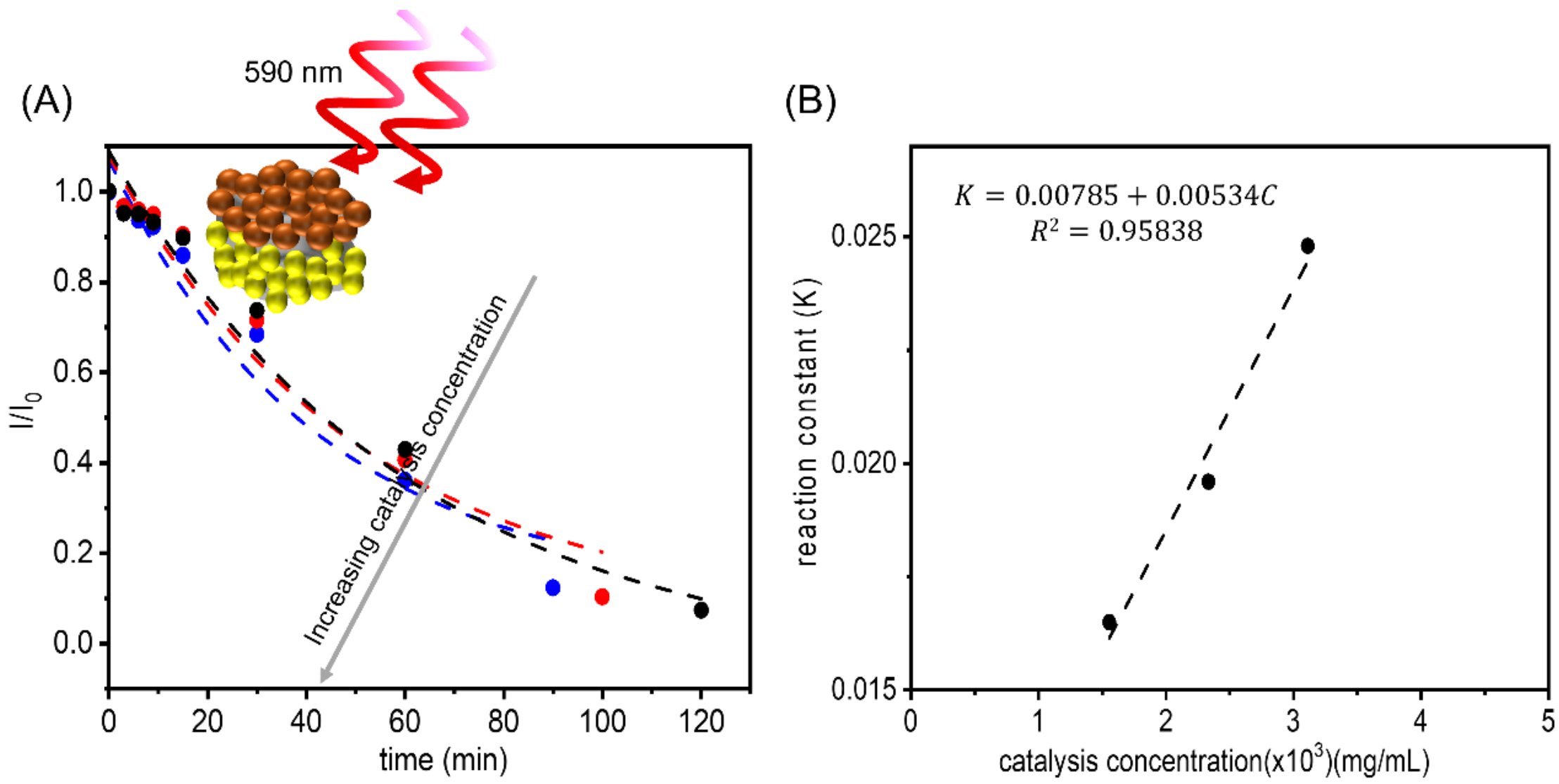

Figure S42 Pt-Ag flower + Au-Ag flower nanocrystals were used as photocatalysts to carry out the reduction reaction of 4-NP under irradiation at a wavelength of 590 nm (off-plasmonic mode of this Janus-like metallic nanocrystal). (A) Concentration change (I/I_0, I: concentration of 4-NP during the reaction, I_0: initial concentration of 4-NP) over time. Solid dots represent experimental data. Dashed lines represent the fitted curve of the first-order reaction mechanism ($\frac{I}{I_0} = y_0 + A \times e^{-(t/t_0)}$ and t_0 = 1/reaction constant (K)). (B) The reaction constant (K) varies with catalyst concentration.

Table S19 The reaction constant (K) of reduction of 4-NP was carried out using Pt-Ag flower + Au-Ag flower nanocrystal as photocatalysts at a wavelength of 590 nm (off-plasmonic mode of this Janus-like metallic nanocrystal) which resulted from $\frac{I}{I_0} = y_0 + A \times e^{-(t/t_0)}$.

Concentration of catalysts($\times 10^3$ mg/mL)	Fitting function : $I/I_0 = y_0 + A*\exp(-t/t_0)$, $K=1/t_0$				
	y_0	A	t_0	$K=1/t_0$	R-square
1.556	-0.059	1.146	60.492	0.0165	0.957
2.333	0.059	1.020	51.031	0.0196	0.944
3.111	0.117	0.956	41.746	0.0239	0.942

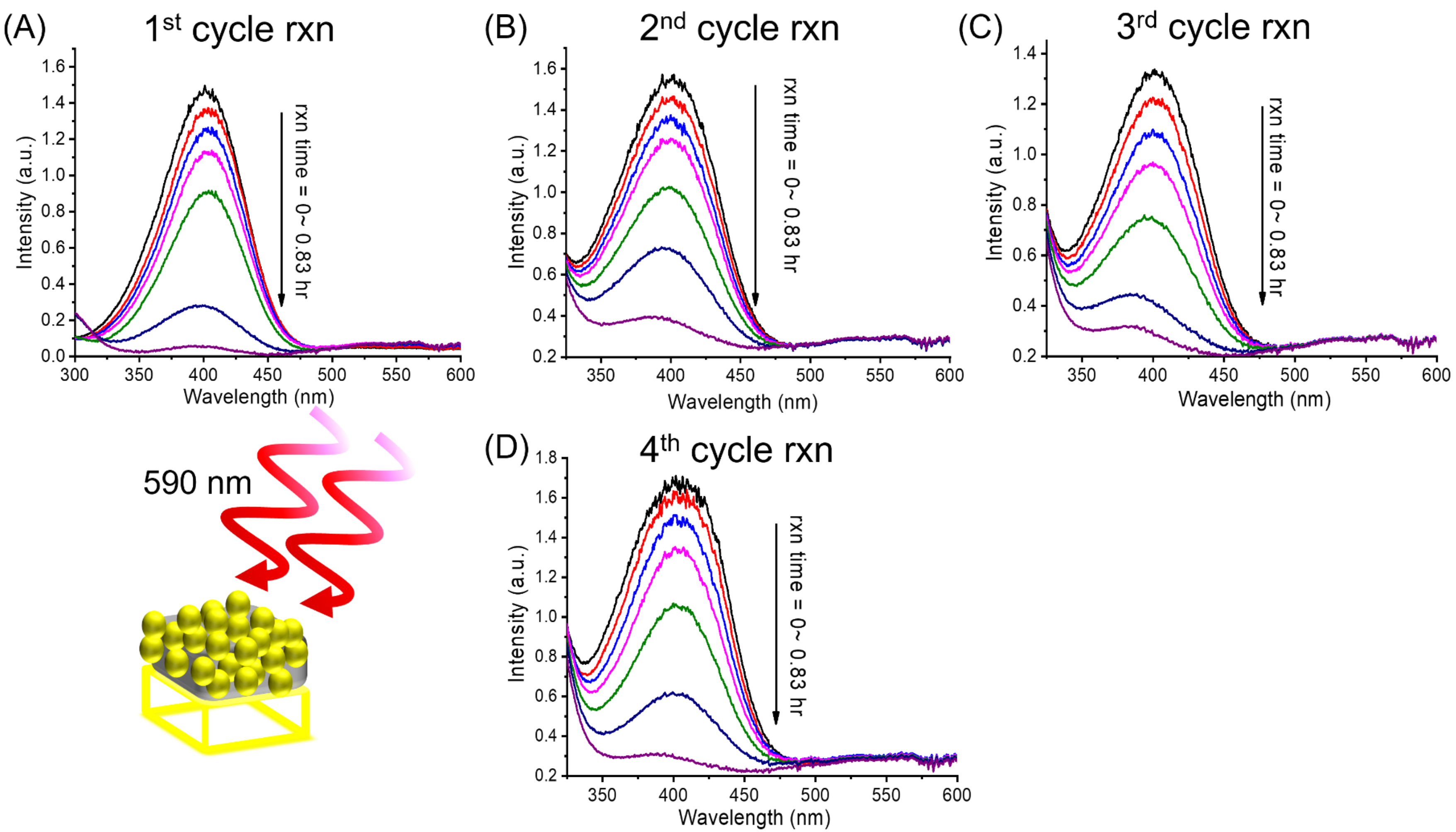

Figure S43 Au-Ag flower + Au-Ag cage nanocrystals were used as photocatalysts to carry out the reduction reaction of 4-NP under irradiation at a wavelength of 590 nm (on-plasmonic mode of this Janus-like metallic nanocrystal). The 4-NP concentration varied with reaction time during 1 to 4 reaction cycles as shown in (A) to (D), respectively.

Table S20 The reaction constant (K) of reduction of 4-NP was carried out using Au-Ag flower + Au-Ag cage nanocrystal as photocatalysts at a wavelength of 590 nm under multi-cycle reaction (on-plasmonic mode of this Janus-like metallic nanocrystal) which resulted from $\frac{I}{I_0} = y_0 + A \times e^{-(t/t_0)}$

Concentration of catalysts ($\times 10^3$mg/mL)	Reaction cycle	Fitting function : $I/I_0 = y_0 + A*\exp(-t/t_0)$, $K=1/t_0$				
		y_0	A	t_0	$K=1/t_0$	R-square
7.732	1st	-0.207	1.251	29.084	0.0336	0.984
	2nd	-0.092	1.120	30.318	0.033	0.994
	3rd	-0.173	1.181	31.304	0.0319	0.986
	4th	-0.164	1.202	34.543	0.0289	0.994

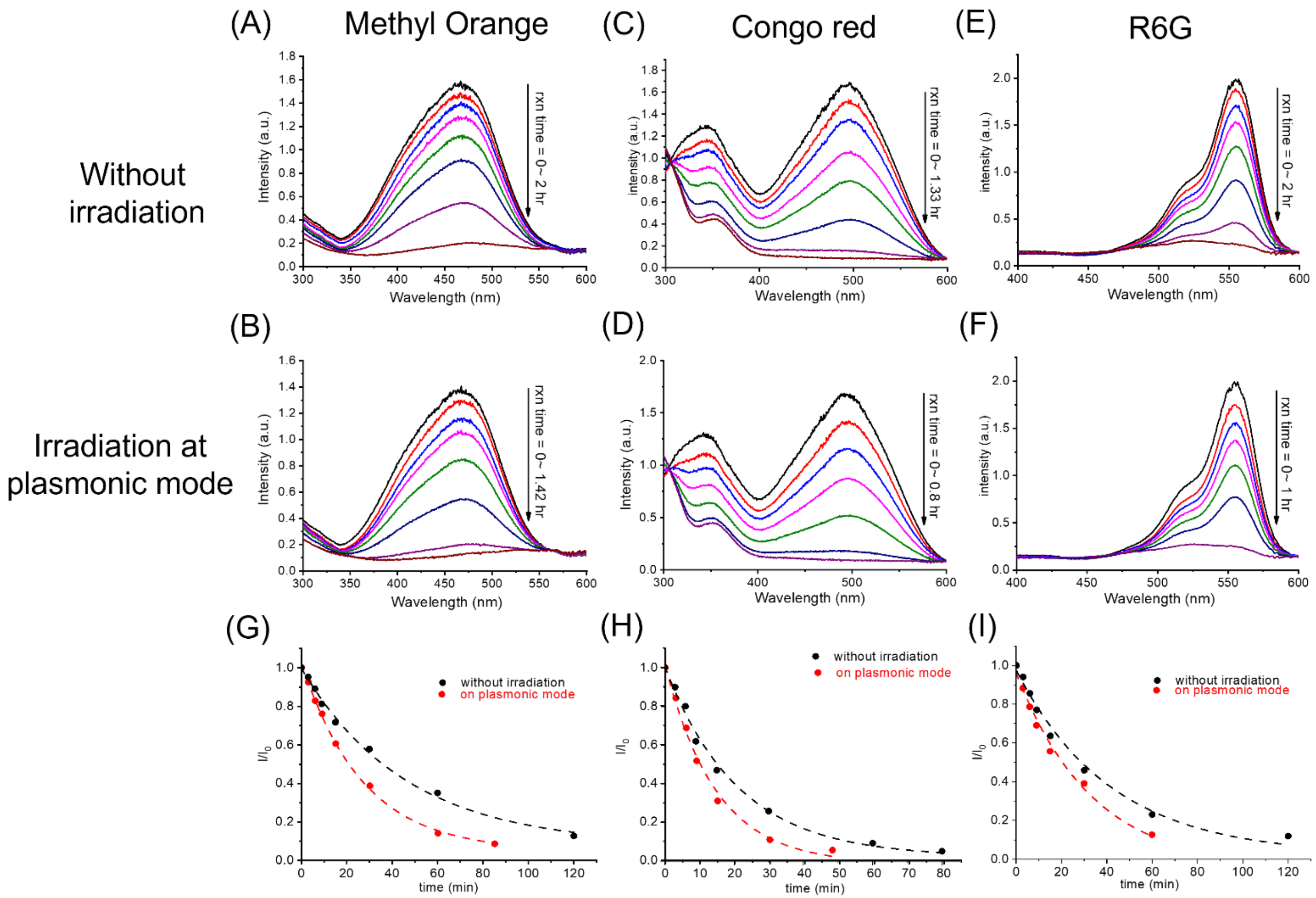

Figure S44 Au-Ag flower + Au-Ag cage nanocrystals were used as catalysts and photocatalysts (on-plasmonic mode of this Janus-like metallic nanocrystal at 590 nm) to carry out the reduction reaction of three different organic dyes. The organic dye concentrations varied with reaction time shown in (A) and (B) for methyl orange; (C) and (D) for Congo red, and (E) and (F) for R6G. Concentration change (I/I_0, I: concentration of organic dye during the reaction, I_0: initial concentration of three organic dyes) over time shown in (G) to (I). Solid dots represent experimental data. Dashed lines represent the fitted curve of the first-order reaction mechanism ($\frac{I}{I_0} = y_0 + A \times e^{-(t/t_0)}$) and B = 1/reaction constant (K).

Table S21 The reaction constant (K) of reduction of organic dyes was carried out using Au-Ag flower + Au-Ag cage nanocrystal as catalysts and photocatalysts at a wavelength of 590 nm (on-plasmonic mode of this Janus-like metallic nanocrystal) which resulted from $\frac{I}{I_0} = y_0 + A \times e^{-(t/t_0)}$.

Organic dye	irradiation condition	Concentration of catalysts ($\times 10^3$mg/mL)	Fitting function : $I/I_0 = y_0 + A*\exp(-t/t_0)$, $K=1/t_0$				
			y_0	A	t_0	$K=1/t_0$	R-square
4-NP	No irradiation	7.109	0.480	0.786	36.676	0.0273	0.990
	590 nm		-0.009	1.061	19.481	0.0513	0.972
MO	No irradiation		0.079	0.919	46.030	0.0217	0.995
	590 nm		0.049	0.971	27.031	0.0370	0.998
CR	No irradiation		0.021	0.982	21.014	0.0476	0.993
	590 nm		-0.0166	1.026	14.655	0.0682	0.993
R6G	No irradiation		0.020	0.953	41.831	0.0239	0.988
	590 nm		-0.051	1.013	33.591	0.0298	0.989

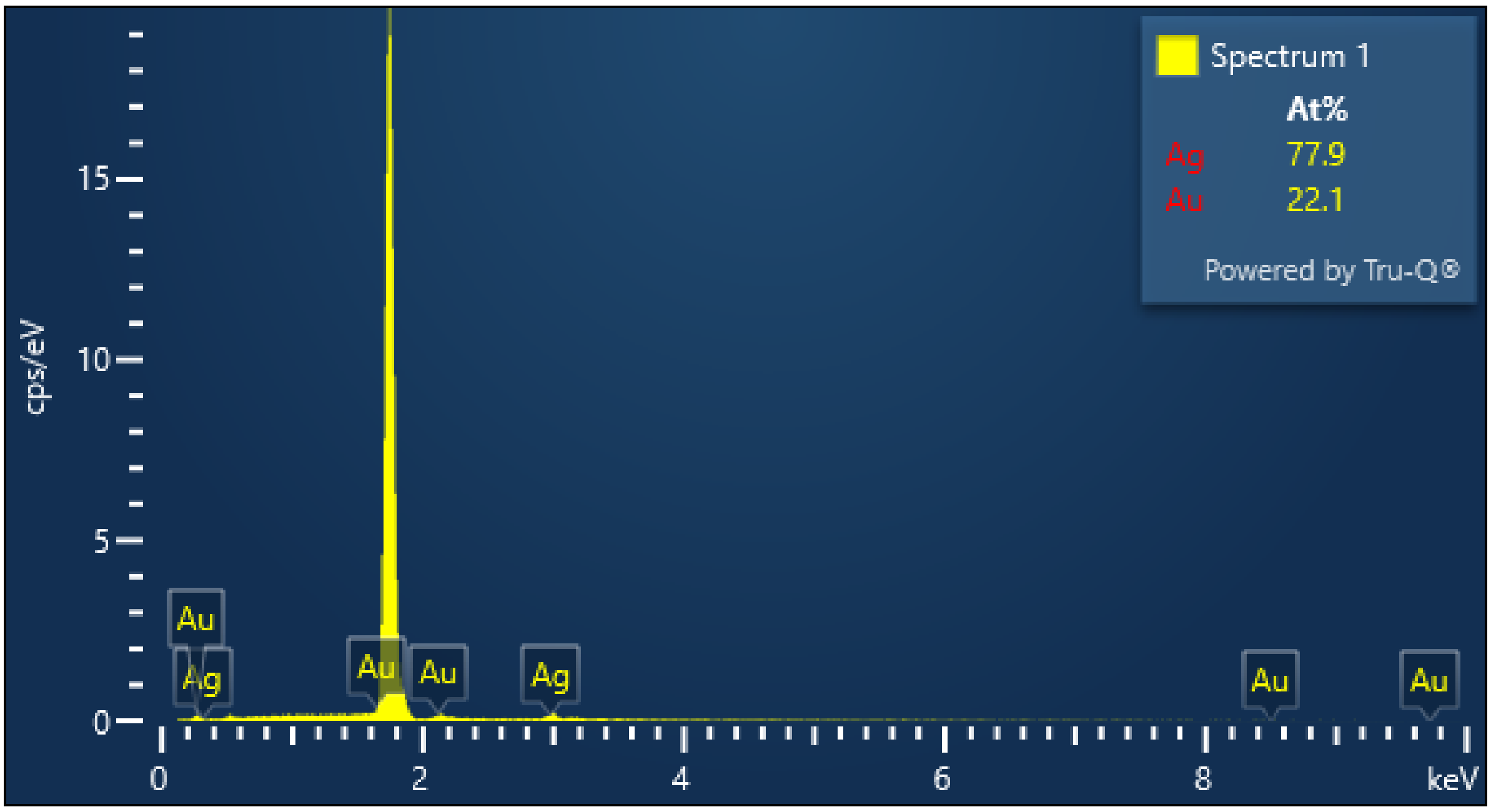

Figure S45 Janus-like Au-Ag cage + Au-Ag flower nanocrystals were used as photocatalysts in the 4-NP reduction reaction, and EDX analysis was performed after four cycles at a wavelength of 590 nm. The results showed that the composition of Janus-like nanocrystals remained almost unchanged. For EDX analysis results of Janus-like Au-Ag cage + Au-Ag flower nanocrystals before 4-NP reduction, as shown in supporting information S6 (B).
